# *Trans* effects of chromosome aneuploidies on DNA methylation patterns in human Down syndrome and mouse models

**DOI:** 10.1186/s13059-015-0827-6

**Published:** 2015-11-25

**Authors:** Maite Mendioroz, Catherine Do, Xiaoling Jiang, Chunhong Liu, Huferesh K. Darbary, Charles F. Lang, John Lin, Anna Thomas, Sayeda Abu-Amero, Philip Stanier, Alexis Temkin, Alexander Yale, Meng-Min Liu, Yang Li, Martha Salas, Kristi Kerkel, George Capone, Wayne Silverman, Y. Eugene Yu, Gudrun Moore, Jerzy Wegiel, Benjamin Tycko

**Affiliations:** Taub Institute for Research on Alzheimer’s Disease and the Aging Brain and Institute for Cancer Genetics, Columbia University Medical Center, New York, NY 10032 USA; The Children’s Guild Foundation Down Syndrome Research Program, Genetics Program and Department of Cancer Genetics, Roswell Park Cancer Institute, Buffalo, NY 14263 USA; Fetal Growth and Development Group, Clinical and Molecular Genetics Unit, UCL Institute of Child Health, London, WC1N 1EH UK; Department of Psychology, Kennedy Krieger Institute and Johns Hopkins University School of Medicine, Baltimore, MD 21205 USA; Department of Developmental Neurobiology, New York State Institute for Basic Research in Developmental Disabilities, Staten Island, NY 10314 USA; Department of Pathology and Cell Biology, Columbia University Medical Center, New York, NY 10032 USA

## Abstract

**Background:**

Trisomy 21 causes Down syndrome (DS), but the mechanisms by which the extra chromosome leads to deficient intellectual and immune function are not well understood.

**Results:**

Here, we profile CpG methylation in DS and control cerebral and cerebellar cortex of adults and cerebrum of fetuses. We purify neuronal and non-neuronal nuclei and T lymphocytes and find biologically relevant genes with DS-specific methylation (DS-DM) in each of these cell types. Some genes show brain-specific DS-DM, while others show stronger DS-DM in T cells. Both 5-methyl-cytosine and 5-hydroxy-methyl-cytosine contribute to the DS-DM. Thirty percent of genes with DS-DM in adult brain cells also show DS-DM in fetal brains, indicating early onset of these epigenetic changes, and we find early maturation of methylation patterns in DS brain and lymphocytes. Some, but not all, of the DS-DM genes show differential expression. DS-DM preferentially affected CpGs in or near specific transcription factor binding sites (TFBSs), implicating a mechanism involving altered TFBS occupancy. Methyl-seq of brain DNA from mouse models with sub-chromosomal duplications mimicking DS reveals partial but significant overlaps with human DS-DM and shows that multiple chromosome 21 genes contribute to the downstream epigenetic effects.

**Conclusions:**

These data point to novel biological mechanisms in DS and have general implications for *trans* effects of chromosomal duplications and aneuploidies on epigenetic patterning.

**Electronic supplementary material:**

The online version of this article (doi:10.1186/s13059-015-0827-6) contains supplementary material, which is available to authorized users.

## Background

It has been more than 50 years since Down syndrome (DS) was shown to result from trisomy 21 (Ts21) but we are still far from understanding how this chromosomal aneuploidy leads to the spectrum of phenotypes in this syndrome. A recent hypothesis invokes epigenetics — the extra chromosome 21 could act in *trans* to produce network perturbations within cells leading to epigenetic alterations, including changes in DNA methylation, which would propagate to daughter cells in developing tissues. To test this idea, we previously performed microarray-based DNA methylation profiling in blood leukocytes from individuals with DS and age-matched controls and found that gains and losses of DNA methylation, affecting about 100 genes, are a stereotypical (i.e., highly recurrent among cases) epigenetic response to Ts21 in these cells [1]. Within this group of genes with DS-specific differential methylation (DS-DM; distinguishing it from cell type-dependent differential methylation and developmental stage-dependent methylation) we noted examples encoding key signal transducing proteins and transcription factors (TFs) necessary for lymphocyte development and function, which likely play a role in the mild immunodeficiency and strongly increased susceptibility to autoimmune disorders in DS. However, that study did not include methylation profiling in purified T cells, leaving as an open question the full repertoire of genes affected by altered methylation in that key cell type. Also, since epigenetic patterning is a cell type-specific phenomenon, data from blood cells will not necessarily generalize to cells in other organs. In fact, Jin et al. reported a group of genes with DS-DM in placentas with Ts21 compared with control placentas that overlapped partially, but not extensively, with those that we had found with DS-DM in blood cells [2].

For understanding the deficits associated with DS, the brain is the organ of greatest interest — intellectual disability is the most consistent feature of the syndrome and Alzheimer’s disease (AD) has an accelerated onset in adults with DS [3]. Since the brain is composed of multiple cell types, steps to separate neurons from non-neuronal cells are expected to improve the yield and accuracy of molecular profiling. Here we show that a gene-specific and tissue-specific epigenetic response to Ts21, recurrent across multiple affected individuals, occurs in neurons and glial cells in DS brains, and in circulating CD3-positive T lymphocytes, and we highlight features of the affected genes and their differentially methylated sequences that point to biological pathways relevant to brain and lymphocyte development and function. Our bioinformatics enrichment analyses support a role for altered TF binding site (TFBS) occupancies in shaping the abnormal methylation patterns. Lastly, as groundwork for future studies, we apply whole genome bisulfite sequencing (WGBS) to DNA from mice engineered to carry sub-chromosomal duplications mimicking human Ts21 and show that the epigenetic signature of human DS brain cells is partly recapitulated in these mouse models.

## Results

### Gene-specific and tissue-specific alterations of CpG methylation in DS brain cells and T lymphocytes

Our overall approach for epigenetic profiling and analysis is diagramed in Figure S1a, b in Additional file 1. As the first step we profiled DNA methylation genome-wide in 14 DS and 8 control frontal cortex (FC) grey matter samples and in 13 DS and 10 control cerebellar folial grey matter samples from age-matched adult autopsy brains (Table S1 in Additional file 2) using Illumina 450K Methylation BeadChips. We additionally profiled CD3-positive T cells purified from peripheral blood of 11 adults with DS and 10 age-matched controls (Table S1 in Additional file 2). We tested for the quality of the samples and reliability of the BeadChip data by reproducibility of the fractional methylation (AVG_Beta) values, which were highly correlated in technical replicates (r > 0.99). We also assessed DNA copy number, using the normalized hybridization intensity values from the methylation arrays, which confirmed full Ts21 in all of the DS cases (examples in Figure S2 in Additional file 1).

Since the brain contains multiple cells types, we used fluorescence-activated cell sorting (FACS) of cell nuclei labeled with anti-Neu-N monoclonal antibody to obtain neuronal (Neu-N-positive) and non-neuronal (Neu-N-negative, hereafter referred to as glial but understood to include the smaller populations of vascular cells and microglia) enriched fractions (Table S1 in Additional file 2; Figure S2 in Additional file 1). Using these preparations in the 450K methylation assays we compared nine individuals with DS with 11 non-DS individuals without significant neuropathology, as well as three cases of non-DS late-onset AD (LOAD). Non-supervised correlation clustering and principal component analysis (PCA) of the 450K data showed that the methylation data for each cell and tissue type clustered separately, consistent with the expected tissue-specificity of DNA methylation patterns (Figure S2 in Additional file 1). We compared AVG_Beta (fractional methylation) values between normal glia and neurons to identify cell type-specific methylation patterns and found 73,216 probes with *p* values < 0.001 (false discovery rate (FDR) < 0.002) and a fractional change in mean methylation ≥ 0.15, 4170 of which had a change in methylation ≥ 0.5, confirming the previously shown epigenetic divergence between these two cell types [4, 5] and validating our decision to separate them.

**Fig. 1:**
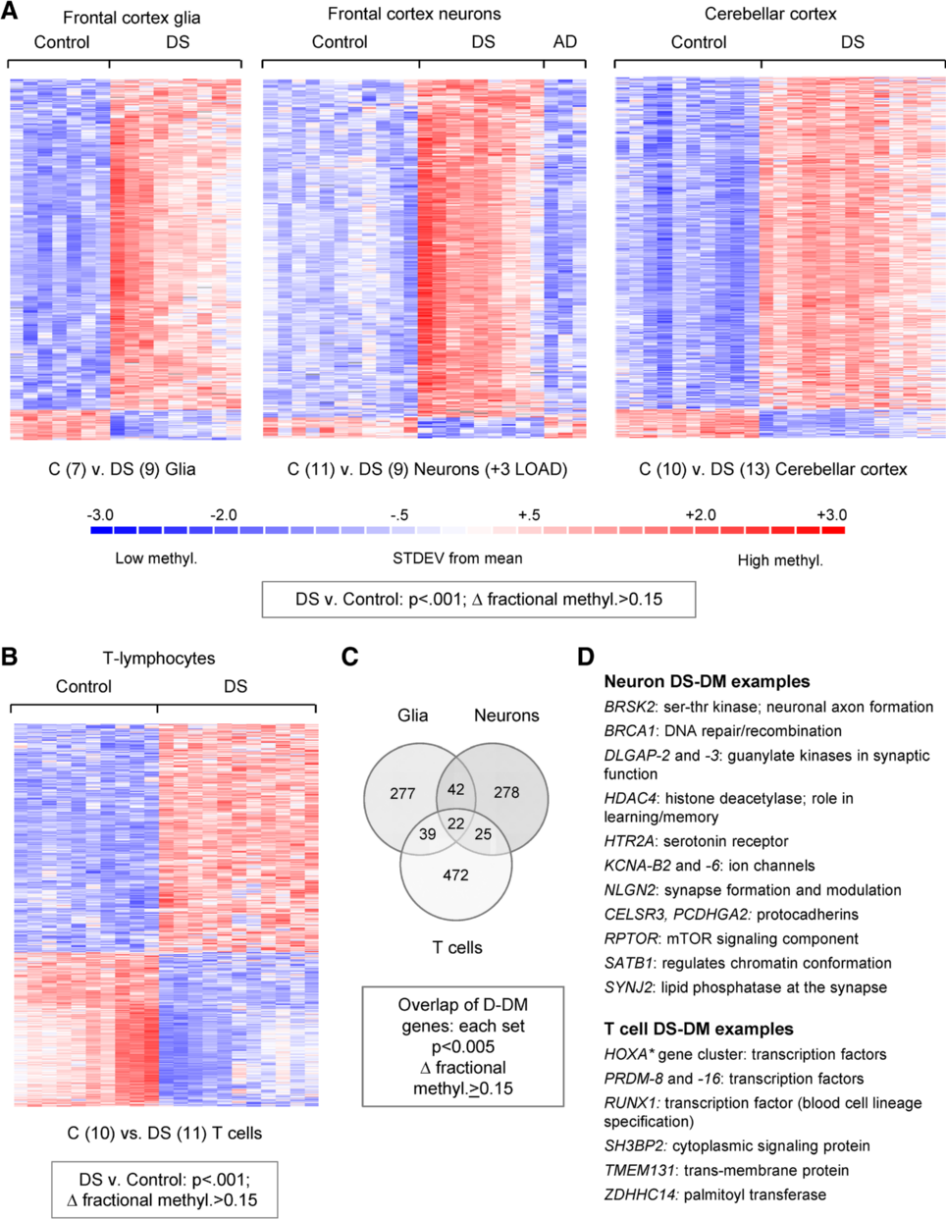
Gene-specific alterations in DNA methylation in DS frontal cortex neuronal and glial cells, cerebellar folial cortex, and T lymphocytes. **a**, **b** Supervised hierarchical clustering of the 450K methylation BeadChip data (fractional methylation values; AVG_Beta) for probes that passed ANOVA at *p* ≤ 0.001 and absolute difference in methylation ≥ 0.15 in DS versus control adult whole frontal cortex, glia, and neurons. Biological samples are on the x-axis and differentially methylated loci are on the y-axis, with relative *hyper*methylation and *hypo*methylation indicated by the color scale. For generating the heatmaps, the fractional methylation values for each CpG were renormalized using the default settings in dChip to have mean 0 and standard deviation 1. The *red color* represents a methylation level above the mean methylation of the CpG across all samples, the *white color* represents mean methylation and the *blue color* represents methylation lower than the mean. **c** Venn diagram emphasizing that many of the DS-DM genes are unique to either neurons or glia, with smaller sets of pan-cell-type DS-DM loci. **d** Partial lists of DS-DM genes, highlighting examples with relevant biological functions. The complete lists of DS-DM CpGs and genes are in Tables S3–S7 in Additional file 2. *STDEV* standard deviation

Unlike the very strong epigenome-wide differences between neurons and glia and between brain cells and T lymphocytes, *within* a given cell type or tissue type non-supervised analysis by correlation heatmap and PCA generally showed only a weak clustering of the AVG_Beta values by DS versus control status, an exception being the DS versus control cerebellar cortex samples, which were well separated by these approaches (Figure S2 in Additional file 1). Further, there were only small differences in global CpG methylation between DS and controls, as indicated by mean AVG_Beta values across all 450K probes (Table S2 in Additional file 2). However, supervised analysis of the 450K data comparing DS versus control samples showed that recurrent CpG methylation abnormalities are indeed present, both in the whole FC grey matter and in the purified neuronal and glial cell nuclei from DS cases, as well as in the DS T cells, compared with the matched controls (Fig. 1). Each tissue and cell type showed a particular DS-DM signature (Fig. 1; Tables S3–S7 in Additional file 2). With stringent unadjusted *p* < 0.001 and delta AVG_Beta > 0.15, we identified 279 genes (578 CpGs) differentially methylated in glia and 272 genes (552 CpGs) differentially methylated in neurons in DS versus controls. In the T cells we found 492 genes (1046 CpGs) with DS-DM at this stringent cutoff. In both glia and neurons our statistical cutoff proved to be robust to multiple testing (unadjusted *p* < 0.001 corresponded to a Benjamini-Hochberg FDR < 0.024 for glia and FDR < 0.028 for neurons; Tables S5 and S6 in Additional file 2; Figure S3 in Additional file 1). In addition, using multivariate analysis, the large majority of DS-DM loci (90–99.5 %) remained significant after adjustment for sex (adjusted *p* value < 0.001) (Tables S5 and S6 in Additional file 2). The findings in cerebellar grey matter and T cells were similarly robust (Tables S4 and S7 in Additional file 2). Very importantly, the reduction in cell type heterogeneity in the purified neuronal and glial nuclear preparations unmasked much larger sets of differentially methylated loci compared with unfractionated FC (Tables S3, S5, and S6 in Additional file 2). Literature and database searches revealed multiple genes with known or suspected roles in brain development and/or function among the DS-DM loci in the neural cells and multiple genes involved in hematopoietic cell differentiation among the T-cell DS-DM loci (Tables S3–S7 in Additional file 2; examples in Fig. 1d).

As shown in Fig. 1 and Tables S3–S6 in Additional file 2, the majority of loci with DS-DM are *hyper*methylated in DS versus control FC brain tissues and brain cells. In contrast, in the CD3-positive T lymphocytes we found approximately equal numbers of hyper- and hypo-methylated CpGs (Fig. 1; Table S7 in Additional file 2), a result consistent with our previous findings in whole blood leukocytes [1]. Supporting the validity of the 450K data, many of the DS-DM loci that we identified in that earlier lower resolution study of unfractionated peripheral blood leukocytes, including *SH3BP2*, *TMEM131* and *CPT1B*, were confirmed in this T-cell DS-DM gene set and, as expected, additional DS-DM loci were identified in the new high resolution data from the purified cells (Table S7 in Additional file 2). The DS-DM loci in each of the tissues and cell types (Tables S3–S7 in Additional file 2) were distributed across most of the human autosomes, with no statistically significant enrichment for genes on chromosome 21, a result that is consistent with our previous findings for loci with DS-DM in unfractionated leukocytes [1].

As highlighted by the Venn diagram in Fig. 1c, DS-DM is a cell type-specific and gene-specific phenomenon, not reflecting a global perturbation of methylation patterns. Supporting this conclusion, while the bias toward hypermethylation in DS brain cells was obvious when examining specific DS-DM genes, assessment of the mean fractional methylation (AVG_Beta) values across all probe sets (non-polymorphic autosomal CpGs queried by the 450K arrays) for DS compared with control samples revealed only a very small (~1 %), though nonetheless statistically significant, genome-wide relative hypermethylation in DS FC and cerebellum compared with controls and no difference in mean methylation in DS versus control T cells (Table S2 in Additional file 2).

We next asked which general classes of DNA sequences are affected by the DS-DM. In FC, the majority of the hypermethylated CpG sites are in CpG islands (CGIs; 73.6 %), with a significant twofold enrichment compared with random expectation based on all CpGs queried by the arrays (*p* = 2 × 10^−12^), and a more modest 1.3 enrichment in gene bodies (including 3′ and 5′ untranslated regions, 66 % versus 51.6 %, *p* = 0.047). A substantial set of promoter regions (1 kb upstream and downstream of the transcriptional start site) showed hypermethylation in DS FC, but there was no selectivity for gains of methylation in such regions compared with non-promoter CG-rich regions. In the neuron preparations we found no enrichment for DS-DM in CGIs, but a modest enrichment in gene bodies (1.4-fold, *p* = 10^−11^), and under-representation in promoter regions (1.7-fold, *p* = 8 × 10^−16^). In glia we found a 1.6-fold enrichment in CGIs (*p* = 7.4 × 10^−22^), a slight enrichment in gene bodies (1.2-fold, *p* = 0.001), and under-representation in promoter regions (1.17-fold, *p* = 0.004), and in the cerebellum, we observed no enrichment for gene bodies and modest under-representations of both CGIs and promoters (1.25-fold, *p* = 2.8 × 10^−13^, and 1.5-fold, *p* = 5.5 × 10^−46^, respectively) (Tables S3–S6 in Additional file 2). In the T cells we found a 1.2-fold enrichment in gene bodies (*p* = 3.652 × 10^−08^), no enrichment in CGIs, and a 1.3-fold under-representation in promoters (*p* = 2.547 × 10^−10^) (Table S7 in Additional file 2).

As an important technical point, although adults with DS are strongly predisposed to developing early onset AD, the DS-DM that we describe here is due to the primary effects of Ts21, not secondary changes from AD. Indeed, supervised hierarchical clustering of fractional methylation values for the DS-DM CpGs showed no evidence of a pattern similar to DS in non-DS LOAD neurons (Fig. 1a), and a direct comparison of DS neurons with non-DS LOAD neurons re-identified a large majority (87.5 %) of the DS-DM loci (FDR < 0.05 and delta AVG_Beta > 0.15) that were found in the comparison of DS with control (neuropathological normal) neurons. This conclusion is further supported by the lack of overlap with the very few genes in AD that were reported by Bakulski et al. as differentially methylated based on 27K BeadChip data [6], and by recent findings in a similar study of AD by Lashley et al. [7], as well as by our analysis showing that the DS-DM loci identified here are not accounted for by accelerated aging (see below).

### Validation and additional mapping of DS-DM in brain cells and T lymphocytes

Since the BeadChip assays query only a small percentage of the CpGs in each CG-rich region, and since single nucleotide polymorphisms (SNPs) in the probe binding sites and overlap of a subset of probes with repetitive sequences can complicate the interpretation, it is essential to validate the data using bisulfite sequencing (bis-seq), which reveals the pattern of methylation across multiple contiguous CpGs near the “index CpGs” queried by the arrays. We chose 13 genes with DS-DM for such validations in brain and three genes with DS-DM loci for validations in T cells, which we performed in the same samples that had been run on the methylation arrays, examining at least two DS and two control samples per gene region. The regions selected for validations were in or near genes relevant to brain or T-cell development or function. Importantly, we did not seek to only validate the strongest DS-DM loci in the 450K data; rather, we included examples spanning a range of high to low differences in methylation and strong to weaker statistical significance.

**Fig. 2:**
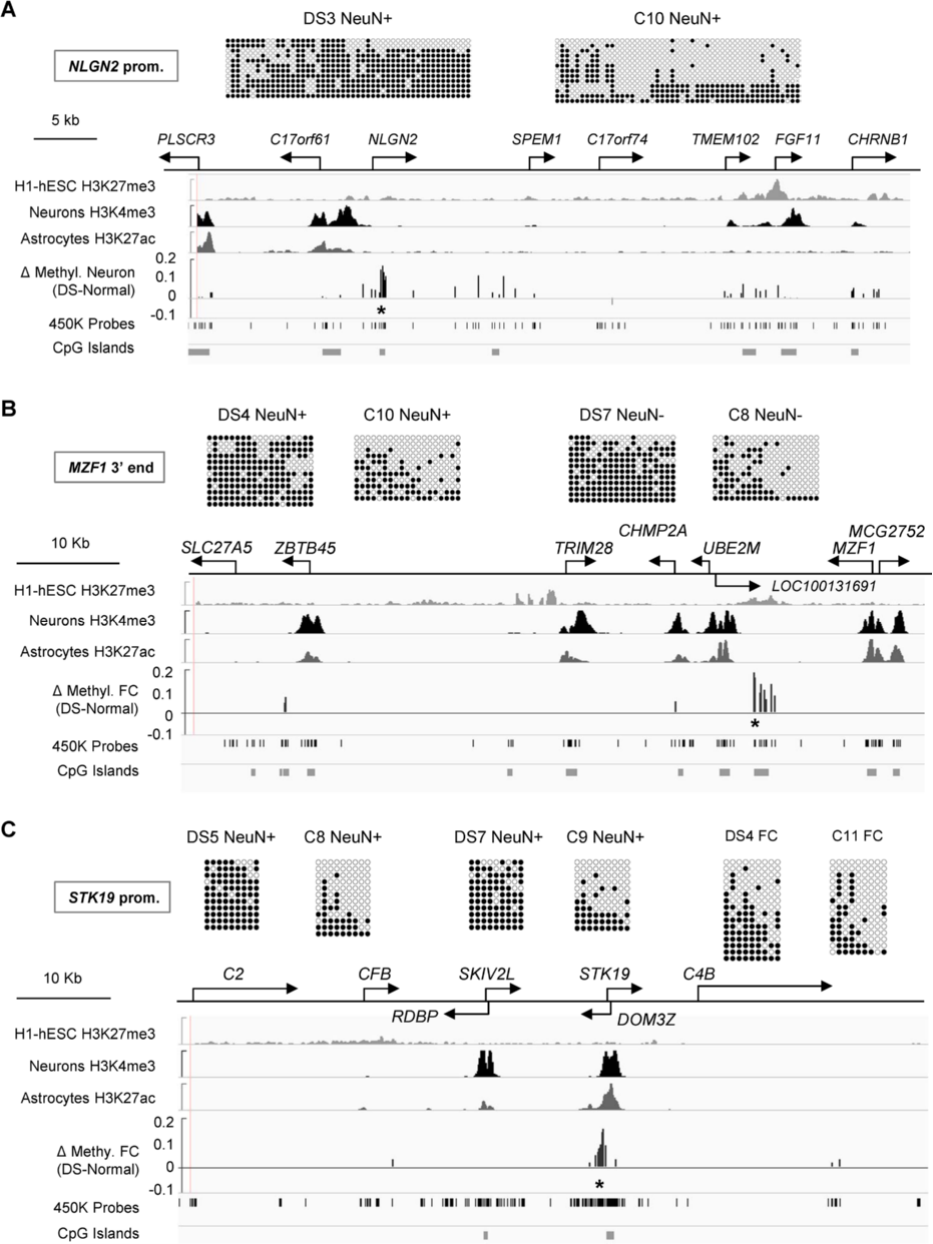
Validation and extension of the results for DS-DM in the *NLGN2*, *MZF1* and *STK19* genes by bis-seq. **a** CpG hypermethylation is localized to the promoter region of the *NLGN2* gene in DS neurons. The bis-seq shows that the DM affects multiple CpGs in the promoter region. As shown by the DM track (Δ *Methyl*; mean difference in AVG_Beta in DS compared with control samples, showing all peaks with a *p* value < 0.001) aligned to ENCODE and Zhu et al. [100] data (GSE17312 and GSM733758, respectively) covering 50 kb of this gene-rich region, strong signals for DM are localized to the downstream edge of the *NLGN2* promoter (marked by the activating H3K4me3 histone modification in H9-derived neurons) and one downstream region, with weaker signals at several other locations. The *asterisk* indicates the position of the bis-seq amplicon. **b** Validation of CpG hypermethylation at the 3′ end of the *MZF1* gene in DNA from purified neuronal and glial cell nuclei. The bis-seq amplicon (*asterisk*) spans a CGI at the 3′ end of the gene that appears to be the promoter for an antisense transcript, *LOC100131691*, which our RT-PCR results show is expressed in fetal brain but not in adult FC (data not shown). Strong DM is restricted to this region, which carries the H3K27m3 poised chromatin mark in human embryonic stem cells (*hESC*). **c** Validation of CpG hypermethylation in the promoter region of the *STK19* gene in DNA from neurons and whole FC. The bis-seq amplicon (*asterisk*) covers the promoter region, which is marked by H3K4m3 in H9-derived neuronal cells. This promoter region also gives rise to an opposite-strand transcript (*DOM3Z*). As is true for the other examples above, the strong DM is tightly localized to this single region

Bis-seq validation of DS-DM in the 5′ end of the *NLGN2* gene, encoding an adhesion protein (neuroligin-2) required for synapse formation, confirmed hypermethylation in DS neurons, and showed that the DS-DM affects multiple CpGs (Fig. 2). There was little or no hypermethylation of this gene in glia and we found an intermediate extent of DS-DM in whole FC grey matter, confirming the conclusion from the array data that hypermethylation of *NLGN2* is neuron-specific. We confirmed gains of methylation in CpGs in a CGI at the 3′ end of the *MZF1* gene in whole FC, neurons, and glia from DS cases by bis-seq (Fig. 2). We also confirmed a gain of methylation in the 5′ promoter region of *STK19*, encoding a Ser-Thr kinase, in the DS neurons and whole FC (Fig. 2). The results of bis-seq also validated hypermethylation in a glia-specific DS-DM region at the 3′ end of the *PDE11A* phosphodiesterase gene, and bis-seq of DNA from glial cells confirmed a differentially methylated region in intron 1 of *HOXA3*, encoding a homeobox TF that is essential for embryonic development [8] and neuronal specification [9] (Figure S4 in Additional file 1). Our bis-seq data also validated and extended the BeadChip findings of gains of methylation in a CGI overlapping intron 1 of the *CPT1B* gene, and showed the presence of DS-DM at multiple CpGs in a regulatory region of the *LRRC14*/*LRRC24* leucine-rich repeat gene pair (Figure S4 in Additional file 1).

While there was no enrichment for chromosome 21-linked genes in the DS-DM gene sets, a few examples were found. *C21orf56* (a.k.a. *SPATC1L*) showed hypermethylation in DS whole FC, as well as in purified neurons and glia, and bis-seq confirmed gains in methylation at the 5′ end of this gene, overlapping the promoter region (Figure S5 in Additional file 1). The *RUNX1* gene, also located on chromosome 21, encodes a TF that plays a role in neuronal specification as well as in the maturation of microglial cells and peripheral blood leukocytes. This gene appeared among the DS-DM loci both in whole FC and in purified glial cell nuclei, which by our FACS protocol would include some (NeuN-negative) microglial cell nuclei, but it did not show DS-DM in the purified neurons. By bis-seq the promoter region of *RUNX1* showed modest gains of CpG methylation in whole FC from the DS brains (Figure S6 in Additional file 1). These gains were seen in only a minority of the bis-seq clones, so these may reflect DM restricted to a minor population of cells, possibly the hematopoietic-derived microglial cells [10]. This interpretation is supported by our finding of much stronger DS-DM for this gene in T lymphocytes (Table S7 in Additional file 2; Figure S7 in Additional file 1). Bis-seq also confirmed a modest increase in CpG methylation in the *ESR1* gene, encoding estrogen receptor-alpha (Figure S6 in Additional file 1). Here too the gain of methylation was seen in a minority of the clones, suggesting that it may be restricted to a sub-population of NeuN-negative cells.

**Fig. 3:**
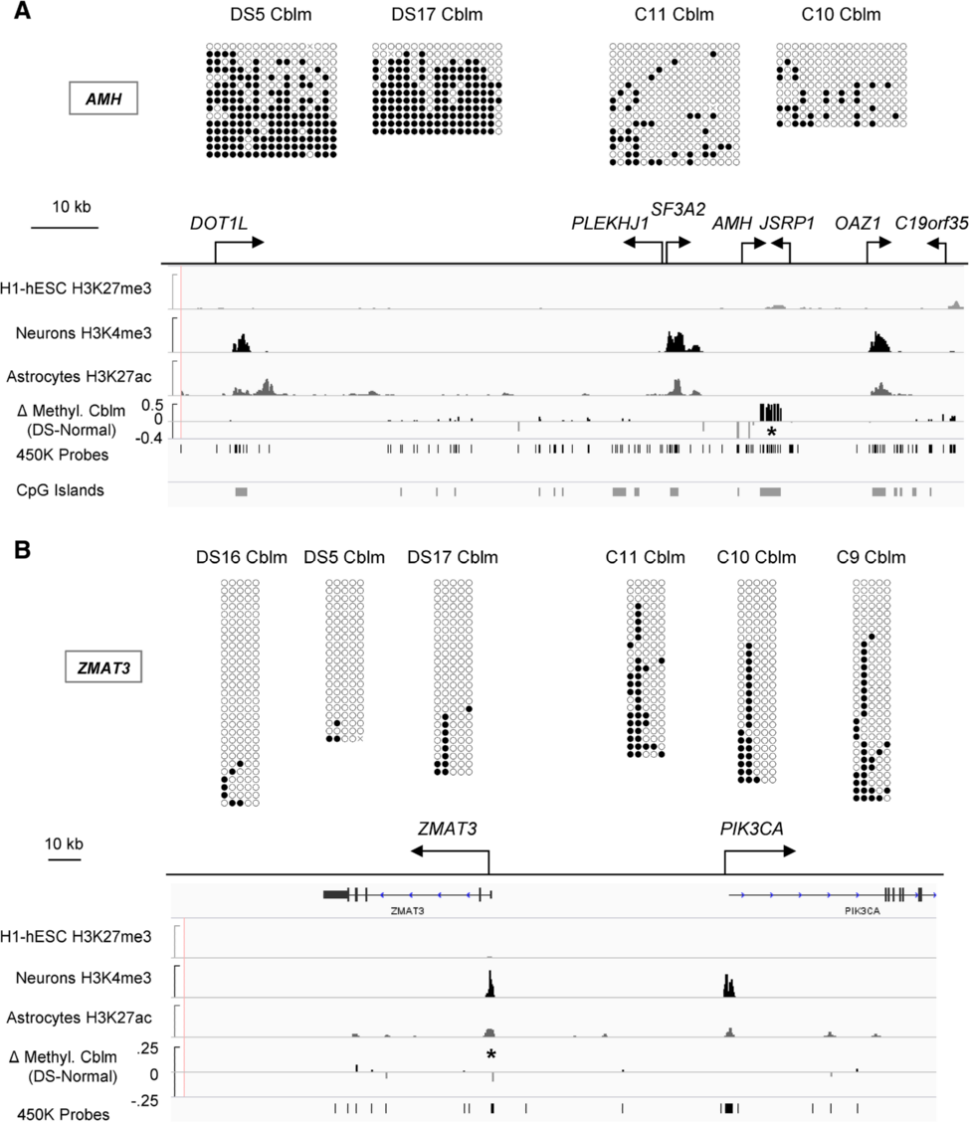
Validation and extension of the results for DS-DM in the *AMH* and *ZMAT3* genes by bis-seq. **a** CpG *hyper*methylation is localized to a large intragenic CGI of the *AMH* gene in DS whole cerebellar cortex. The array data and the results of bis-seq both show that the DM affects multiple CpGs. As shown by the DM track (Δ *Methyl*; mean difference in AVG_Beta in DS compared with control samples, showing all peaks with a *p* value < 0.001) aligned to ENCODE and Zhu et al. [100] data (GSE17312 and GSM733758, respectively), strong signals for gain of methylation are localized to this region (marked by a peak of H3K27me3 in h1-ESC) and there is loss of methylation at two positions upstream of the CGI. The *asterisk* indicates the position of the bis-seq amplicon. **b** CpG *hypo*methylation localized to the *ZMAT3* promoter region in DS cerebellar cortex is confirmed by bis-seq. One of the two affected CpGs is in a binding site for the GABPA TF (Figure S9 in Additional file 1). The *asterisk* indicates the position of the bis-seq amplicon. *hESC* human embryonic stem cell

Next we used bis-seq to validate the DS-DM in three genes in the cerebellar cortex. *AMH*, showing strong DS-DM in this tissue and also in the FC, encodes anti-Mullerian hormone. Bis-seq confirmed a strong gain of methylation localized to its CGI (Fig. 3). Another gene with DS-DM in cerebellum, *ZMAT3*, is a p53 target that is expressed in brain cells. Our bis-seq data confirmed DS-DM in an upstream regulatory region of this gene, but unlike many of the other DS-DM loci, *ZMAT3* showed *hypo*methylation in the DS samples. Interestingly, the DS-DM in this gene was limited to two contiguous CpGs (Fig. 3), one of which is in a binding motif for GABP-alpha (a.k.a. NRF2), a TF that is encoded by a gene on chromosome 21 and shows methylation-sensitive DNA binding [11]. Bis-seq also confirmed an intragenic gain of methylation in the hedgehog signaling pathway gene *GLI2* in the DS cerebellar cortex samples (Figure S8 in Additional file 1). Another GLI-family gene, *GLI4*, also appeared in the DM gene set in cerebellum, as well as in FC glia (Tables S4 and S5 in Additional file 2). Lastly, the three DS-DM loci that we selected for bis-seq in DS versus control T cells were *RUNX1*, encoding a cell fate TF in hematopoietic lineages [10, 12, 13]; *ZDHHC14*, encoding a protein palmitoyl transferase that may impact protein kinase signaling [14]; and *LRNN3*, encoding a leucine-rich protein that is also involved in cell signaling and is expressed in T cells [15]. Bis-seq confirmed the array data, and indicated involvement of multiple contiguous CpGs in the DS-DM for each of these genes (Figure S7 in Additional file 1). Comprehensive maps of the 450K methylation data for several of the above featured genes in brain and T cells, showing the locations of the DS-DM CpGs with respect to high-, intermediate- and low-methylated CGIs, are in Figures S8–S10 in Additional file 1.

### One group of DS-DM loci reflects additive contributions of 5mC plus 5hmC while another group has differences mostly in 5hmC

Since standard bis-seq does not distinguish between 5mC and its hydroxylated derivative 5hmC, we used a modified bis-seq protocol (BS/OXBS; see "Materials and methods") to determine the relative contributions of 5mC and 5hmC to the DS-DM in four genes: *STK19* and *MZF1* in the cerebrum and *ZMAT3* and *GLI2* in the cerebellum. As shown in Figures S11 and S12 in Additional file 1, both types of modified base contributed to the DS-DM. Of these four loci the strongest component of 5hmC was seen in the *ZMAT3* upstream region in the cerebellum (Figrues S11 and S12 in Additional file 1). These results fit with the fact that among human organs the brain has one of the highest levels of 5hmC, with cerebellum having even greater 5hmC content than cerebrum [5, 16].

To extend this analysis we used BS/OXBS as the first step in probe preparation for 450K BeadChips. We analyzed the resulting data in two ways: first examining the BS/OXBS data specifically for the DS-DM CpGs that we had identified in the larger series of DS cases and controls using the standard Illumina 450K protocol, and second, using probe sets obtained by applying t-test and absolute difference criteria directly to the BS/OXBS data from this smaller group of cases and controls. The results from the first approach are summarized in Figure S12 in Additional file 1, which highlights groups of CpGs in which both 5mC and 5hmC contribute to the net DS-DM to differing extents. Strikingly, for nearly all of these CpGs (>90 %) the direction of the difference in fractional methylation is seen to be the same for 5mC and 5hmC (Figure S12 in Additional file 1; Table S8a–c in Additional file 2). This result supports previous suggestions that these two modifications act coordinately as stable chromatin marks in brain cells [17, 18]. Our second approach, analysis of the data from the BS/OXBS experiment not restricted to the prior DS-DM probe sets, revealed three groups of differentially methylated sequences: one essentially the same as that discussed above in which 5mC and 5hmC contribute additively to the DS-DM, and two other groups, in which the sole or major contribution to the DM is from one of these two marks and not the other (Table S8b, c in Additional file 2). Interestingly, the “5hmC-only” DS-DM loci (uncorrected *p* value < 0.05 and difference in fractional methylation > 0.15; 959 DS-DM CpGs) showed frequent *hypo*methylation in DS compared with controls; a trend opposite to that seen with the 5mC + 5hmC and 5mC-only DS-DM regions, which are more frequently *hyper*methylated in DS. Many of the “5hmC-only” DS-DM CpGs are in genes that play known or suspected roles in brain development and function, examples being *PTPRN2*, *SLIT1*, *EFNA2*, *CBFA2T3*, and the neurotransmitter receptor genes *GABRB3* and *GABRA5* (Table S8a, b in Additional file 2).

### Altered DNA methylation in DS brains begins early in development and preferentially affects developmentally regulated genes

Since intellectual disability in DS is due to abnormal neurodevelopment, we next asked whether the DS-DM in DS brain cells might have its onset at the fetal stage. We used 450K BeadChips to compare eight mid-gestation (13–18 weeks) DS fetal whole cerebrum brain samples with six gestational age-matched control samples. After applying t-test and percentage change criteria as in our adult brain analyses, we found a set of DS-DM loci in the DS versus control fetal brains, again showing predominantly hypermethylation in the DS cases (Figure S13 in Additional file 1; Table S9 in Additional file 2). Using a cutoff of *p* < 0.005 and delta AVG_Beta > 0.15 for DS-DM in each gene set, 18 of 65 genes (28 %) that showed DS-DM in both neurons and glia at the adult stage already showed DS-DM at the fetal stage and 21 of 56 genes (38 %) with DS-DM in unfractionated adult FC already showed DS-DM at the fetal stage. Thus, a substantial component of the epigenetic response to Ts21 in DS brain cells has its onset early in brain development. This epigenetic response to Ts21 in neurogenic precursor cells affects multiple genes with roles in brain development. Examples include *AMIGO3*, encoding a transmembrane protein that is essential for correct axon tract development [19], *CELSR3* and *PCDHGA2*, encoding a protocadherin involved in neuronal connectivity [20–23], *CYTH2*, coding for cytohesin-2, with a role in neurite extension [24], and *GLI4* (Figure S13 in Additional file 1), which is expressed in the brain [25] and, by analogy to the other GLI family members, is predicted to control cell differentiation.

To test statistically whether the altered methylation in fetal and adult DS brains preferentially affects developmentally regulated genes, we utilized data from an array-based study of mRNA expression comparing normal fetal and adult human brains [26]. Expression data were available for 15,298 of the genes that were queried by the 450K arrays, including 236 of the genes with DS-DM in glia and 202 in neurons (gene list cutoffs at *p* < 0.001). Differential expression in this dataset was assessed by t-tests across technical replicates, requiring *p* < 0.05; expression was higher in fetal compared with adult brain for 3769 genes, higher in adult brain for 4341 genes, and similar in adult and fetal for 7188 genes. As shown in Table S10 in Additional file 2, enrichment for genes with higher expression in fetal brain was observed among the DM genes in neurons (fold enrichment = 1.3; *p* = 0.002). In contrast, we found no enrichment for genes with stage-specific mRNA expression in the glia DS-DM gene set. To follow up this finding we asked whether the genes with DS-DM in DS versus normal brain cells might be enriched in examples with brain-specific expression or brain-specific repression. Expression data across various human tissues were downloaded from the Allen Brain Atlas database (http://www.brain-map.org/) and bioGPS. When we classified these genes based on their expression across the multiple tissues we found a modest but significant enrichment in genes with brain-specific expression in neurons (36 % of the DS-DM genes versus 28 % of the BeadChip gene content, *p* = 3.7 × 10^−5^; Figure S14 in Additional file 1). No such enrichment was observed in a parallel analysis of the glia DS-DM gene set.

### DNA methylation patterns show early maturation in DS

Since some forms of intellectual disability have been associated with differences in brain maturation, we next investigated the gestational age dependence of epigenetic changes in DS compared with control fetal brains. We found 5657 CpGs with significant methylation changes across control fetal brain development (univariate analysis, methylation changes > 0.1 per 10 weeks, R squared ≥ 0.8 and uncorrected *p* value < 0.05), including 2147 with progressive hypermethylation and 3510 with progressive hypomethylation. We performed multivariate linear regressions for each of these gestational age-dependent CpGs, with disease status, developmental stage, and the interaction term between disease status and developmental stage as explanatory covariates. In this model, the interaction term reflects the longitudinal effect of the disease (across age or developmental stage), while the disease status term reflects the difference at baseline (Table S11a in Additional file 2). The results suggested an early maturation of the methylation patterns in the DS fetal brains compared with the control fetal brains, as summarized by the graph of the mean fractional methylation of the 54 CpGs with gestational age effects in controls (univariate analysis) and for which age and DS versus control status explained more than 80 % of the variance (adjusted age *p* value < 0.05 and adjusted R squared form multivariate analysis ≥ 0.8) (Figures S13 and S15 in Additional file 1). We observed a significant interaction between DS versus control status and gestational age, which reflects a differential maturation between DS and controls (differential effect in gestational age dependent *hypo*methylation CpGs = 0.2 per 10 weeks, *p* = 2.6 × 10^−5^, and differential effect in gestational age dependent *hyper*methylation CpGs = 0.13 per 10 weeks, *p* = 7 × 10^−4^; Figures S13 and S15 in Additional file 1). In both probe sets, the differential effect is explained by a smaller and negligible gestational age effect in DS compared with controls (−0.05 versus −0.27 per 10 weeks and 0.09 versus 0.22, respectively). When we tested for a more complex differential aging effect by looking for interactions between DS versus control status and age, without requiring age effects in the normal samples, we identified 30 additional CpGs with a significant though mild differential aging effect (adjusted R squared > 0.8, adjusted age effect *p* < 0.05, age effect > 0.1 per 10 weeks, *p* value of the interaction term < 0.05 and difference of the age effect between DS and normal > 0.1 per 10 weeks; Table S11a in Additional file 2). These data confirmed early maturation of CpG methylation patterns in DS. When we performed linear regression of the methylation average across the 1 % of CpGs with the most significant changes in methylation in the fetal compared with adult brains we found no evidence of hypermaturation of methylation patterns in the adult DS brains. Thus, while our data suggest *early* maturation in DS fetal brains, the endpoint is not *hyper*maturation.

Importantly, we found that early maturation of methylation patterns in DS is not restricted to brain cells; in T cells, where our sample set is larger than for the fetal brains, we found that 168 CpGs show a significant differential age effect (adjusted R squared > 0.8, adjusted age effect *p* value < 0.05, and *p* value of the interaction term <0.05; Table S11d in Additional file 2). When the methylation of these CpGs is graphed versus age, examination of the slopes and Y intercepts of the DS and control lines leads to conclusions that parallel those in the fetal brains. While we did not study T cells from children the simplest interpretation of the slopes and Y intercepts of the adult T-cell data is that there is early maturation of methylation patterns in DS T cells, without subsequent accelerated aging of these patterns (Figure S16 in Additional file 1).

### DS-DM in brain cells and T lymphocytes is not accounted for by age effects

We next asked whether any of the DS-DM loci identified from our case–control analysis (151 CpGs defined by *p* < 0.001; delta AVG_Beta > 0.15) were gestational age-dependent, and we found only two DS-DM CpGs overlapping with the age-dependent set (Table S11a in Additional file 2; Figure S13 in Additional file 1). Thus, the strong DS-DM CpGs that we report here in fetal brains are due to Ts21 status per se and are not secondary to differential brain maturation rates. Likewise, we asked whether age effects might account for a component of the strong DS-DM in the adult DS brains and T cells. Although 39 CpGs in the total 450K BeadChip content showed an age effect on their methylation levels in control adult unfractionated FC after univariate linear regression (R squared ≥ 0.8, *p* value ≤ 0.05), these showed very small fractional methylation changes over time (average = 0.004 per 10 years) and graphs of the mean values for these loci showed no evidence of accelerated methylation aging in the adult DS brains (Figure S16 in Additional file 1). Consistent with this fact, we found no overlap of the strong DS-DM CpGs from adult FC (sets defined by *p* < 0.001; delta AVG_Beta > 0.15) with these age-dependent CpGs (Table S11b in Additional file 2; Figure S16 in Additional file 1). These results were confirmed in the purified neuron samples, with only two CpGs showing age dependent methylation, and no differential aging effects between DS and controls for the DS-DM CpGs (Figure S17 in Additional file 1). Similar negative results were obtained when assessing the 348 age-dependent CpGs and the 2719 DS-DM CpGs in cerebellum (Table S11c in Additional file 2). In the T cells, 1022 CpGs showed a significant age effect in control samples after univariate analysis, with once again only small methylation changes per 10 years (average = 0.02 per 10 years; Table S11d in Additional file 2). We found no overlap between the set of CpGs with strong DS-DM (1046 CpGs; set defined by *p* < 0.001; delta AVG_Beta > 0.15) and this age-dependent CpG set (Figure S16 in Additional file 1; Table S11d in Additional file 2).

To be even more complete, using multivariate linear regressions, we looked for CpGs with a significant differential aging effect between age and DS versus control status, regardless of whether they were identified as age-dependent in the control samples. In FC, cerebellum and T cells, we identified 3, 46, and 168 CpGs, respectively, with a differential age effect between DS and controls (adjusted R squared > 0.8, adjusted age effect *p* value < 0.05, and *p* value of the interaction term < 0.05) with most of the CpGs showing aging effects in control samples but not in DS (Table S11b–d in Additional file 2). Once again, among the 2719 strong (*p* < 0.001; ΔAVG_Beta > 0.15) DS-DM CpGs in cerebellar cortex, only six showed a difference in the age-dependence of methylation in DS versus controls, while in T lymphocytes among the 1046 CpGs with strong DS-DM (*p* < 0.001; ΔAVG_beta > 0.15), only 24 showed a difference in the age-dependence of methylation in DS versus controls (Table S11b–d in Additional file 2). In FC, we found no overlap of DS-DM CpGs with the small set of CpGs that showed age-dependent methylation.

### Some but not all of the DS-DM genes have altered mRNA expression in DS brains

While the main emphasis of this study is DNA methylation, gene expression is obviously also of interest. We carried out quantitative PCR (Q-PCR) for mRNAs of eight genes with DS-DM, asking whether the DS-DM was associated with differences in expression in DScompared with control brains. The mRNA expression showed high intra-group variability in the DS and control samples, likely due to factors including different agonal conditions among the autopsy brains, but we nonetheless found significant inter-group differences in expression of some, though not all, of these genes. *STK19* showed robust mRNA expression in the adult brain samples, and the DNA hypermethylation in DS, affecting the promoter region of this gene, was associated with decreased mRNA expression (Figure S18 in Additional file 1). In addition, we found that expression of *STK19* mRNA in normal human astrocytes was increased by exposure of these cells in culture to the demethylating drug 5aza-deoxycytidine (5aza-dC; Figure S18 in Additional file 1). In contrast to *STK19*, Q-PCR for *NLGN2* mRNA revealed increased mRNA expression in the DS brains (Figure S18 in Additional file 1). The DS-DM in this gene is also localized to the promoter, but there is some prior evidence that promoter methylation may normally activate transcription of *NLGN2*: MeCP2, a methylated CpG-binding protein that can either activate or repress genes and associates with the transcriptional activator CREB1 [27], can bind one of the *Nlgn2* promoters in mice, and activate transcription in reporter assays [28]. We also analyzed expression of an interesting DS-DM gene from the fetal brain data, *GLI4*. The hypermethylation of this gene was found both in the adult DS glial cells and in the fetal DS whole FC samples and the DM was localized to the 3′ region, not the promoter. Q-PCR revealed that *GLI4* mRNA is over-expressed on average in DS fetal brains (Figure S18 in Additional file 1), suggesting that the downstream element may have a negative regulatory function. While these examples gave positive results, for two other genes with strong and recurrent DS-DM that we assayed by Q-PCR, namely *CPT1B* and *MZF1*, we found a wide range of expression in both DS and controls, with no difference in mean expression between the two groups (data not shown). For *CPT1B*, this negative result might be accounted for by the fact that the DS-DM region, an intragenic CGI, has promoter activity only in mesenchymal cells, as suggested by ENCODE data.

We also used Q-PCR to assess expression of three interesting genes, *EFNA3*, *DUSP1* and *CYBA*, that showed DS-DM in cerebellar cortex. Of these genes, *EFNA3*, encoding the neural signaling receptor Ephrin-A3, showed a clear difference in mRNA levels between DS and control cerebellar cortex samples, with higher mRNA expression and promoter *hypo*methylation (as well as relative *hyper*methylation in the gene body) in DS (Figure S18 in Additional file 1). However, despite having DS-DM in regulatory sequences, the *DUSP1* and *CYBA* genes did not show a significant difference in mRNA expression in DS versus control cerebellar cortex (data not shown). In summary, some but not all of the brain DS-DM genes (four of the eight genes tested) showed differential expression between DS and controls in the samples available for our analysis. Possible explanations for lack of correlations between expression and methylation at some loci include variable acute changes in expression due to different agonal states prior to death and fundamental biological factors such as a role for the DS-DM at earlier stages of brain development that were not available for our analysis. Nonetheless, each of the genes for which a correlation between methylation and expression was found (*GLI4*, *EFNA3*, *STK19* and *NLGN2*) have known or predicted roles in brain development and/or neural function.

### Enrichment analyses link DS-DM to brain and T-cell development and indicate a mechanism involving altered TFBS occupancies, including RUNX1 sites in T cells

To gain mechanistic insights, we used bioinformatic enrichment analysis, comparing the sets of DS-DM loci with the entire 450K gene and CpG content, to test for over-representation in the DS-DM loci of: (i) genes and CpGs with cell type-specific methylation, (ii) CpGs with developmental stage-specific methylation, and (iii) CpGs in regions with specific histone modifications. Details are in the "Materials and methods", and the lists of *p* values are in Figures S19 and S20 in Additional file 1 and Tables S12 and S13 in Additional file 2. We found that the sets of loci with DS-DM in adult DS versus control FC neurons and glia, and cerebellar cortex, are all enriched in genes whose methylation is dynamically regulated during cell type specification in brain development, and that methylation differences in DS compared with control brain cells preferentially affect genes whose CpG methylation increases between the fetal and adult stages. We also observed, by overlapping the DS-DM CpG sets with ENCODE tracks and by performing gene set enrichment analysis (GSEA; http://software.broadinstitute.org/gsea/index.jsp), that a group of genes that are normally repressed by the polycomb repressive complex 2 (PRC2) in embryonic stem cells and then activated (derepressed) during normal glial differentiation are preferentially subject to DS-DM (specifically *hyper*methylation) in DS glia and in the whole brain samples (that contain numerous glial cells), with a weaker enrichment for this type of mark in the purified neuron and T-cell DS-DM sets (Table S12 in Additional file 2).

As these findings suggested a mechanism in which perturbed transcriptional networks during cell differentiation might underlie the DS-DM, we next asked whether altered methylation occurred more often near tissue-specific TFBSs. We used two complementary approaches (Figure S21 in Additional file 1). First, we looked for de novo predicted motifs overlapping the DS-DM CpGs, using the HOMER tool. This approach can identify known TFBS motifs and also novel motifs, which the program predicts based on the input data. Using our data from brain cells, alignment of the positions of the HOMER-predicted motifs to ENCODE data showed only sparse overlap with ChIP-Seq-validated TFBSs, leaving the biological meaning of many of the de novo predicted motifs uncertain. Therefore, to test whether these sequences might be elements that bind nuclear factors that do not have a known consensus binding sequence, we identified the strongest enriched ChIP-Seq peaks at the genomic positions of the HOMER de novo motifs. Consistent with our GSEA and poised chromatin enrichment analysis, two of the HOMER-predicted motifs were enriched within EZH2, and to a lesser extent within KDM5B peaks in ENCODE (*p* = 4.2 × 10^−14^ and *p* = 6.1 × 10^−3^, respectively). EZH2 belongs to PRC2 and KDM5B is a H3K4 demethylase that has also been associated with PRC2 [29]. In humans, specific sequence motifs that recruit PRC2 have not been identified, but accumulating evidence supports a role for *cis*-regulatory sequences in the recruitment of polycomb complexes [30–32]. In our T-cell data the set of *hypo*methylated DS-DM CpGs analyzed by HOMER showed strong enrichment in de novo motifs aligning with RUNX1 and AP1 (JUN/FOS) motifs (Tables 1 and 2; Table S13a in Additional file 2). Confirming the biological relevance of this result, the HOMER-predicted motif instances were also strongly enriched within bona fide RUNX1 and FOS ChIP-Seq peaks (Tables 1 and 2; Table S13b in Additional file 2).

**Table 1:** TFBS enrichment analysis points to altered TFBS occupancy as a mechanism underlying some examples of tissue specific and tissue-invariant DS-DM

Cell type	Best match	HOMER alignment score	Percentage of DS-DM CpGs (n = 419)	Percentage of background CpGs (n = 437,650)	*P* value	Enriched TF peaks among HOMER motif instances in ENCODE ChipSeq data (*p* < 0.05 and OR ≥ 1.5)
T cell hypomethylated	RUNX	0.79	25.3	11.0	1.00E-15	RUNX
T cell hypomethylated	BATF::JUN (FOS)	0.948	21.0	7.9	1.00E-16	FOS/JUN

Top ranked TFBSs from HOMER de novo motif searches, showing a strong enrichment among T-cell hypomethylated loci in DNA sequences with a high alignment to RUNX and JUN/FOS consensus motifs, validated by a finding of enrichment in RUNX and JUN/FOS binding peaks in ChIPSeq data from ENCODE. The full de novo motif search results for all DS-DM sets are in Table S14a in Additional file 2. *OR* odds ratio

**Table 2:** TFBS enrichment analysis points to altered TFBS occupancy as a mechanism underlying some examples of tissue-specific and tissue-invariant DS-DM

Loci with DS-DM shared across multiple tissues	Encode motifs	Odds ratio (enrichment)	*P* value	FDR	Count in DS-DM CpGs	Count in background	Percentage of DS-DM CpGs	Percentage of background CpGs
54 DS_DM CpGs shared by all brain tissues and cell types: hypermethylated set (diff. > 0.15, *p* > 0.005)	CTCF_ext	8.03	1.09E-09	6.65E-08	10	6989	14.29	2.04
	MEF2	22.33	1.75E-09	6.65E-08	4	933	5.71	0.27
	CTCF	2.34	0.0015	0.039	19	47,093	27.14	13.72
	USF	2.66	0.035	0.67	5	9661	7.14	2.81
47 DS-DM CpGs shared by all tissues and cell types (including T cells): hypermethylated set (diff. > 0.15, *p* < 0.005)	CTCF_ext	9.09	1.57E-10	1.20E-08	10	6989	15.87	2.04
	MEF2	24.98	5.10E-10	1.94E-08	4	933	6.35	0.27
	CTCF	2.32	0.0030	0.075	17	47,093	26.98	13.72
	USF	2.98	0.019	0.37	5	9661	7.94	2.81

TFBS enrichment among loci with DS-DM shared across multiple tissues, showing a strong enrichment in CTCF binding motif instances (as identified in ENCODE) in 200-bp windows containing the DS-DM CpGs. For the full TFBS enrichment results in all DS-DM sets see Table S14b in Additional file 2

Second, we tested for enrichment in validated TF motif instances in 200-bp windows around each DS-DM CpG, using data in ENCODE TF tracks. By this approach the enrichment in RUNX1 and AP1 motifs was again the strongest result for the T cell *hypo*methylated DS-DM CpGs (Tables 1 and 2; Table S13b in Additional file 2). RUNX1 is a generally repressive TF that is encoded on chromosome 21 and over-expressed in DS [33]. Since prior studies have shown that TFBS occupancy can lead to active or passive demethylation of DNA [34–36], RUNX1 overexpression and increased RUNX1 binding site occupancy might account for a substantial component of the locus-specific hypomethylation observed in DS T cells, as well as contributing to the immune abnormalities in DS. The observed gains of methylation in a portion of the promoter of the RUNX1 gene itself might therefore reflect an ineffective or partially effective attempt at autoregulation. Likewise, c-Fos has clear roles in T-cell function [37], and Jun-Fos has been suggested to bind CpG-methylated AP-1 sites with increased affinity, mediating transcriptional activation [38]. These observations suggest that hypomethylation observed in T cells at AP1 sites might be paradoxically repressive and might also contribute to altered immune function in DS. In the T-cell hypomethylated DS-DM set we also found enrichment in TFBSs for NFKB1, PU1, POU2F2 (OCT2), and MEF2 (FDR < 0.05; Table S13b in Additional file 2), which are involved in development and function of T lymphocytes [39–42]. With regard to T-cell and brain *hyper*methylated CpGs, the enrichment for validated TF binding sites was more modest and accounted for fewer DS-DM CpGs (Table S13b in Additional file 2). Nonetheless, among the TFs with enriched motifs, SOX2 and MEF2 family members are involved in neuronal function and cell differentiation [43–45].

### Differentially methylated regions showing tissue-invariant DS-DM are enriched in CTCF and MEF2 sites

While the majority of DS-DM loci are cell type-specific, there is some overlap between the DS-DM gene sets for T lymphocytes and brain cells. The lists of these “overlap genes”, including examples such as *BRCA1*, *CPT1B*, and *STK19* that have very strong DS-DM, are in Table S14 in Additional file 2. The differentially methylated regions for this group of tissue-invariant DS-DM genes are enriched in CTCF and MEF2 binding motifs (Tables 1 and 2; Table S13b in Additional file 2). CTCF mediates transcriptional insulation and plays a role in brain and lymphocyte development [46–49]. It binds preferentially to unmethylated sequences and is implicated, along with other DNA binding proteins, in the maintenance and formation of low methylated regions, suggesting that reduced CTCF occupancy might account, at least in part, for these tissue-invariant hypermethylated DS-DM loci [34, 50–52]. As the number of tissue-invariant DS-DM CpGs is small, this finding cannot reflect a genome-wide deficit in CTCF or MEF2; rather, it seems likely that the local sequence context of each CTCF or MEF2 site plays a role in producing these DS-DM loci, probably through combinatorial interactions with other DNA binding proteins.

### The epigenetic signature of human DS brain cells is partly recapitulated in brains from Dp(10)1Yey and Dp(16)1Yey mice

Although the above analyses point to altered TFBS occupancy and subsequent altered CpG methylation patterns as a mechanism underlying DS-DM, manipulative experiments to test this and other mechanisms will require animal models. Such models have been constructed in mice by chromosome engineering [53]. Two of these lines, Dp(10)1Yey and Dp(16)1Yey, contain duplications of the regions of mouse chromosomes 10 and 16 that have conserved synteny with human chromosome 21. Using genomic DNAs from newborn whole cerebral hemispheres from mice with each of the murine sub-chromosomal duplications and from a littermate wild-type (wt) mouse, we carried out WGBS to ≥28-fold mean depth. To confirm trisomy in the regions spanning chr10:76207226–78464948 in Dp(10)1Yey and chr16:75540514–97962621 in Dp(16)1Yey in the sequenced DNA samples, we computed the ratio of the coverage between each of the two partial trisomy mice and their wt littermate, after normalization by the total number of reads mapping to these chromosomes. As shown in Figure S22 in Additional file 1, this procedure revealed the expected gains of DNA copy number mapping precisely to the engineered duplicated regions. In contrast, the ratios of CpG methylation showed no obvious differences across the duplicated genomic regions, suggesting the absence of dosage compensation-like effects (Figure S22 in Additional file 1). This result was confirmed when comparing methylation between the duplicated and not duplicated genomic regions of chromosome 10 and 16 in each mouse (Figure S23 in Additional file 1). Examining all chromosomes, Dp(10) mouse cerebrum shows a mild global hypermethylation compared with the control mouse cerebrum (∆fractional methylation = +0.0017), while Dp(16) shows a mild global hypomethylation (∆fractional methylation = −0.01) (Figure S24 in Additional file 1).

Next, we searched for strong gene-specific differences in CpG methylation patterns between the Dp(10)1Yey, Dp(16)1Yey and wt brains, asking whether alterations in these patterns might partly mimic the findings in human DS brains. Paralleling the human findings, ... hypermethylated CpGs in Dp(10) and Dp(16) compared with control mouse were slightly enriched in CGIs, CGI shores and gene bodies but under-represented in promoter regions. Hypomethylated CpGs in Dp(10) and Dp(16) were modestly enriched in CGI shores and gene bodies but under-represented in CGIs and promoter regions (Figure S24 in Additional file 1). We next compared the mouse WGBS data with DS-DM in our human array-based data, only considering CpGs in genomic windows corresponding to the regions covered by the Illumina 450K BeadChips. Of 299,267 such CpGs that could be mapped to the mouse genome, 25,333 and 25,960 were found in 1-kb windows with at least one DM CpG, including 55 % and 61 % CpGs with gains of methylation in Dp(16)1Yey and Dp(10)1Yey brains, respectively, compared with the wt control brain. When considering only methylation changes with the same direction in human and mouse, these windows encompassed 53 % of all human DS-DM genes identified in neurons or glia (absolute methylation changes > 0.15 and *p* value < 0.005), which reflected a 1.9-fold enrichment of Dp(10)1Yey and Dp(16)1Yey DM genes in human DS-DM genes (*p* = 2.5 × 10^−28^ and 1.5 × 10^−24^, respectively; Table S15 in Additional file 2). For human DS-DM genes present in both neurons and glia, the enrichment in mouse DM genes was even stronger (2.5-fold enrichment, *p* = 2 × 10^−6^ for dp10 and 2.3-fold enrichment, *p* = 4 × 10^−5^ for dp16; Table S15 in Additional file 2) with 63 % of these DM genes concordantly affected in either Dp(10)1Yey or Dp(16)1Yey.

**Fig. 4:**
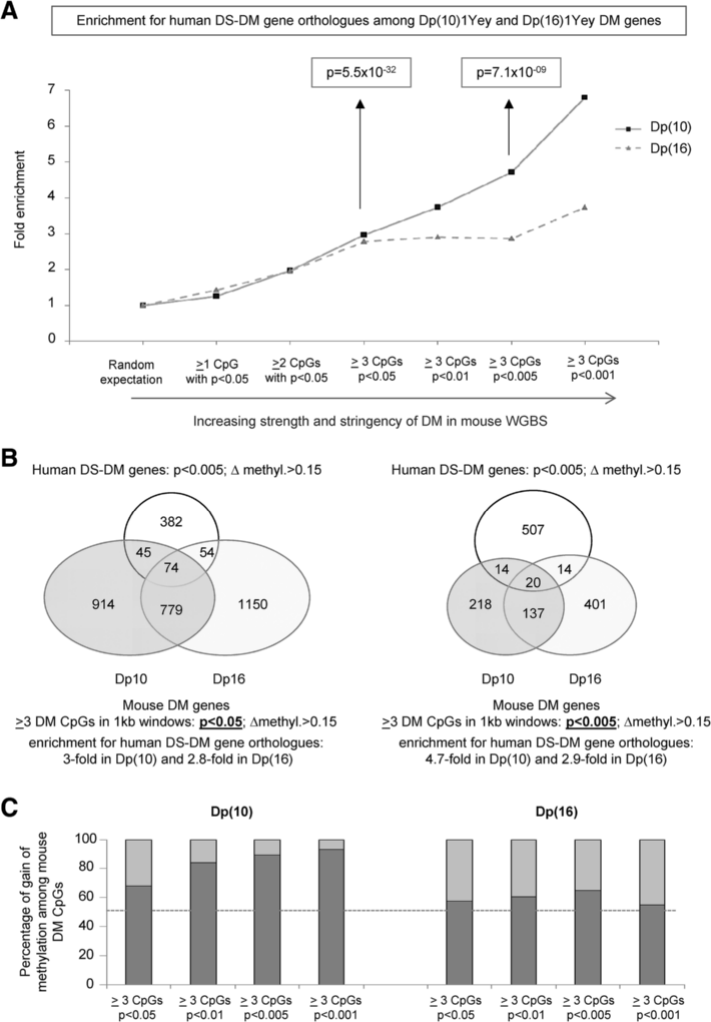
Parallels between DS-DM in humans and DM in chromosome-engineered mouse models of DS. **a** In the mouse WGBS data each 1-kb genomic window corresponding to all evaluable regions queried by the human 450K arrays was evaluated for DM using stringent criteria employing Fisher exact tests at the individual CpG level (see "Materials and methods"). Using these sets of DM loci, we asked whether the human DS-DM genes identified in our comparisons between DS and control human brain cells (neurons and/or glia) were statistically enriched among the mouse DM genes. Although the number of DM genes decreases with increasing stringency, there is a progressive relative enrichment for human DS-DM gene orthologues in the mouse DM gene sets as the stringency requirements for DM in the mouse data are increased. The *p* values for enrichment using the combined Dp(10)1Yey and Dp(16)1Yey data at the indicated stringency levels are from hypergeometric tests. Highly significant enrichment for human DS-DM genes was also seen separately in both the Dp(10) and Dp(16) DM gene sets (respectively, *p* = 1.5 × 10^−27^ and 2.5 × 10^−27^ at 0.05 stringency level and *p* = 7.1 × 10^−9^ and *p* = 3.2 × 10^−5^ at the 0.005 stringency level). **b** The Venn diagram on the *left* shows that a substantial group of human DS-DM genes (31 %) show DM at orthologous genomic locations in the mouse models, using a Fisher exact test *p* value of 0.05 as the criterion for DM of individual CpGs in the mouse data, and that there is only partial overlap between the DM gene sets found in Dp(16) and Dp(10). The Venn diagram on the right shows the genes that overlap when a more stringent *p*-value is applied to the WGBS data. **c** Stacked bar graphs showing the relative frequencies of gains (*dark grey*) and losses (*light grey*) of methylation in the two mouse models, compared with wt, for CpGs corresponding to the regions covered by the human 450K arrays. Paralleling the situation in human brains, both mouse models show preferential gains of methylation (above the *dashed line*), with this effect being stronger in Dp(10) than in Dp(16)

Importantly, as we increased the stringency for calling DM in the mouse data, either by ranking each gene for the strength of DM using a composite confidence score based on the geometric mean of the median of differential methylation (DM) across all significant DM CpGs, and the number of DM CpGs in the window (data not shown), or by requiring progressively more stringent Fisher exact test *p* values for DM at each CpG (Fig. 4; Figure S25 in Additional file 1) we observed a progressive and highly significant enrichment in human DS-DM genes among the mouse DM genes. Again, while the DM gene sets in Dp(10)1Yey and Dp(16)1Yey were only partly overlapping, a significant enrichment for human DS-DM genes was found in both sets (Fig. 4; Figure S25 in Additional file 1), suggesting that genes in both of the duplicated regions participate in bringing about the net downstream epigenetic response. A second parallel between the human and mouse data is in Fig. 4c, which shows using the stringent approach that the brains from both mouse models have more gains than losses of methylation in the genomic windows corresponding to the human 450K arrays, with this bias toward gains being strongest in Dp(10)1Yey.

**Fig. 5:**
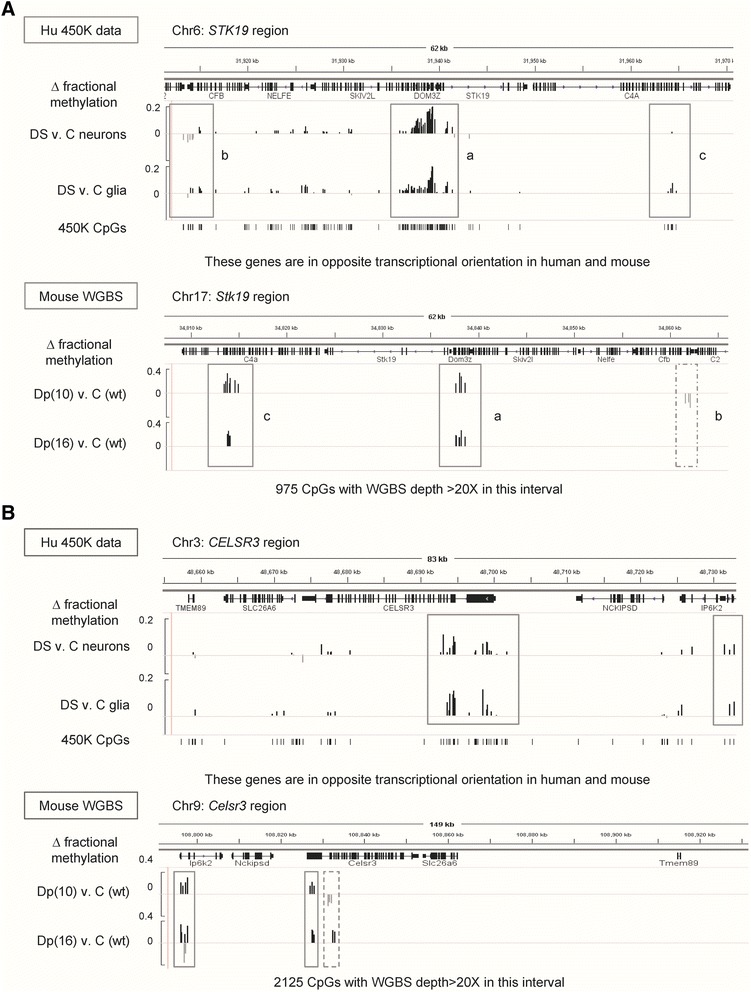
Gene maps showing parallels between DS-DM in humans and DM in the chromosome-engineered mouse models. **a** Maps of the *STK19*/*Stk19* genes and their flanking genes in human and mouse. A cluster of CpGs between *STK19* and *DOM3Z* is concordantly hypermethylated in human brain cells and in both Dp(10)1Yey and Dp(16)1Yey (*solid rectangles*). At other locations some differences in methylation patterns are seen between Dp(10)1Yey and Dp(16)1Yey (*dashed rectangle*). For the human data, the values for all CpGs with a significant difference in methylation between DS and controls (*C*; uncorrected *p* < 0.05) are shown. For the mouse data, DM CpGs were defined as CpGs with > 15 % difference in methylation and with at least two other DM CpGs with the same direction of methylation change, within 1-kb windows. Only CpGs in 1-kb genomic windows at positions orthologous to those queried by human 450K arrays are considered. The mouse maps span 975 CpGs with WGBS depth > 20× in this chromosome region; of these, 22 showed DM by the above criteria. The (*a*) and (*b*) clusters of DM CpGs remain significant after applying additional Wilcoxon *p* value criteria (*p* < 0.05) across the cluster. **b** Maps of the *CELSR3/ Celsr3* genes and their flanking genes in human and mouse. For the human data, the values for all CpGs with a significant difference in methylation between DS and controls (uncorrected *p* < 0.05) are shown. A cluster of CpGs in the promoter region of *CELSR3* is concordantly hypermethylated in human brain cells and both Dp(10)1Yey and Dp(16)1Yey, as is a cluster in the *Ip6k2* gene (*solid rectangles*). Additionally, in the first exon of *Celsr3* concordant hypermethylation is observed for Dp(16)1Yey, while hypomethylation is present in Dp(10)1Yey (*dashed rectangle*). The mouse maps span 2125 CpGs with WGBS coverage > 20× in this chromosome region; of these, 34 showed DM. Each of the boxed clusters of DM CpGs remains significant after applying additional Wilcoxon *p* value criteria (*p* < 0.05)

Given that the profiling methods utilized were different (WGBS for mouse and 450K BeadChips for human), and that neither of the two partial trisomy models recapitulate the entire human chromosome 21, these results in Dp(10)1Yey, Dp(16)1Yey and human DS are impressively concordant. Moreover, additional parallels were seen by “zooming in” on specific DS-DM loci and comparing the maps of DM between the mouse models and the human brain cells and tissues. As shown by the two examples in Fig. 5 and the five additional examples in Figures S26–S28 in Additional file 1, some of these loci (*STK19*, *MZF1*, *FAM83H*, *LRRC24*, *PCDHGA2*) showed methylation changes paralleling the human data in both Dp(10)1Yey and Dp(16)1Yey brains while other loci ( *CPT1B*, *CELSR3*) showed changes at some positions within the gene that parallel the human data in only one of the two mouse lines. Comprehensive maps of the WGBS data for several of the above featured genes, showing the locations of the DM CpGs with respect to high-, intermediate- and low-methylated CGIs, are in Figures S29–S31 in Additional file 1.

## Discussion

The etiology of DS, namely the extra copy of chromosome 21, is well established, but the pathogenesis of the major phenotypes in this syndrome is still not fully understood. Findings in mouse models have implicated increased dosage of several chromosome 21-linked genes, including *DYRK1A*, *RCAN1*, and *SYNJ1*, in the neurocognitive phenotype (reviewed in [54, 55]), but mechanisms beyond simple gene dosage likely also come into play. Candidates include gene–gene interactions leading to perturbations of transcriptional networks, and epigenetic changes including altered DNA methylation that might contribute to, or alternatively partly compensate for, these network perturbations.

We previously showed that Ts21 leads to changes in CpG methylation in a set of about 100 genes in peripheral blood cells of adults with DS [1]. More recently a similar phenomenon of epigenetic alterations, affecting a different set of genes with a few overlaps, was found in placentas from DS [2]. Here we have taken this approach in a series of brains from adults (cerebrum and cerebellum) and fetuses (cerebrum) with and without Ts21, supplemented by data from DS and control T lymphocytes. Our results show that there is indeed a recurrent epigenetic response to Ts21 both in neurons and in non-neuronal (mostly glial) brain cells, with predominantly gains of CpG methylation in discrete sets of genes on various chromosomes. Our approach of separating neuronal from non-neuronal cell nuclei turned out to be crucial for identifying neuron-specific DS-DM loci, and different anatomical regions of the brain showed only partly overlapping sets of DS-DM genes. Based on our data from the fetal brains, many of the epigenetic changes are initiated early in brain development and affect developmentally regulated genes.

Among the genes with DS-DM in DS neural cells is a high percentage with roles in brain development or function, while many of the DS-DM genes in T cells have important roles in lymphocytes. Members of the neuroligin protein family are essential for synapse formation, with *NLGN2* being expressed at GABAergic synapses in the hippocampus. In mice this gene is implicated in behavior [55], and it and other neuroligin genes are implicated in human psychiatric disorders and intellectual disability [56–59]. *PCDHGA2* encodes a protocadherin family member, with a possible role in dendrite development [23, 60] and *CELSR3* encodes a protocadherin-related protein necessary for correct neuronal connectivity [20–22]. Additional genes with DS-DM in brain cells include *BRCA1*, which has been shown to be genetically essential for brain development [61, 62], and *SYNJ2*, encoding a lipid phosphatase that modulates vesicle trafficking and is associated with cognitive abilities in humans [63]. The *HTR2A* gene codes for a serotonin receptor and is associated with behavioral traits, schizophrenia and depression [64], *KCNAB3* encodes a subunit of a voltage-sensitive potassium channel, and *SPNS1* codes for the Spinster-1 protein implicated in apoptosis in the *Drosophila* central nervous system [65, 66]. Among the genes with DS-DM in fetal DS brains are several of the above, plus *AMIGO3*, encoding a membrane protein essential for correct axon tract development [19], *BRSK2*, encoding the SAD1B kinase that plays a role in polarization of cortical neurons [67, 68], *CYTH2*, coding for an Arf6 guanine-nucleotide exchange factor, cytohesin-2, with a role in neurite extension [24], and *GLI4*, which is known to be expressed in the brain based on microarray data [25] and, by analogy to the other GLI family members, is predicted to control cell differentiation.

The *STK19* gene encodes a widely expressed nuclear serine/threonine kinase involved in cell signaling and regulation of cell proliferation [69]. The *MZF1* gene encodes a zinc finger TF (myeloid zinc finger 1) that regulates granulopoiesis [70]. However, *MZF1* is also expressed in the brain and there is some evidence suggesting a role in AD [71–73]. Membrane proteins encoded by genes in the leucine-rich repeat family, represented in the DS-DM genes by *LRRC24* and *LRRC14*, have roles in synaptic function, and both are expressed in the brain [74]. Regarding the *AMH* gene, with DS-DM in cerebral and cerebellar cortex, circulating anti-Mullerian hormone levels correlate to performance on social aptitude tests among individuals with autism spectrum disorder [75], and sex-dependent cognitive phenotypes have been demonstrated in DS [76]. *AMH* may also regulate the number of Purkinje cells in the cerebellum [77]. The *GLI2* gene, encoding a TF in the sonic hedgehog pathway, is likewise biologically relevant, as Shh is vital for specification of the cerebellum early in development, and post-natal treatment with an Shh agonist ameliorated the aberrant cerebellar morphology observed is Ts65dn mice [78]. Lastly, three members of the ephrin-A gene family, *EFNA2*, *EFNA3* and *EFNA5*, which code for neural signaling receptors, showed strong DS-DM in DS versus control cerebellar cortex.

DNA methylation patterns change rapidly in fetal development [79] and can also change as mature tissues age [80]. Moreover, DS has been associated with some aspects of premature aging in adults [81, 82]. Our data here point to early maturation of CpG methylation patterns in DS brains, as well as in T lymphocytes, without hypermaturation of these patterns and without accelerated methylation aging. Our conclusions in this regard differ from Horvath et al. [83], who used 450K BeadChips to compare CpG methylation patterns in unfractionated cerebral cortex samples from four DS versus 17 control brains and concluded that DS is associated with accelerated epigenetic aging. A discussion of possible reasons for this difference is in Additional file 3. The *early* maturation that we have described here differs from that seen in autism and other forms of intellectual disability, where brain *hyper*maturation has been raised as a hypothesis [84–87], but it suggests that future studies might productively search for differences in the rate of maturation of neuronal axons, dendrites and synapses in DS brains.

**Fig. 6:**
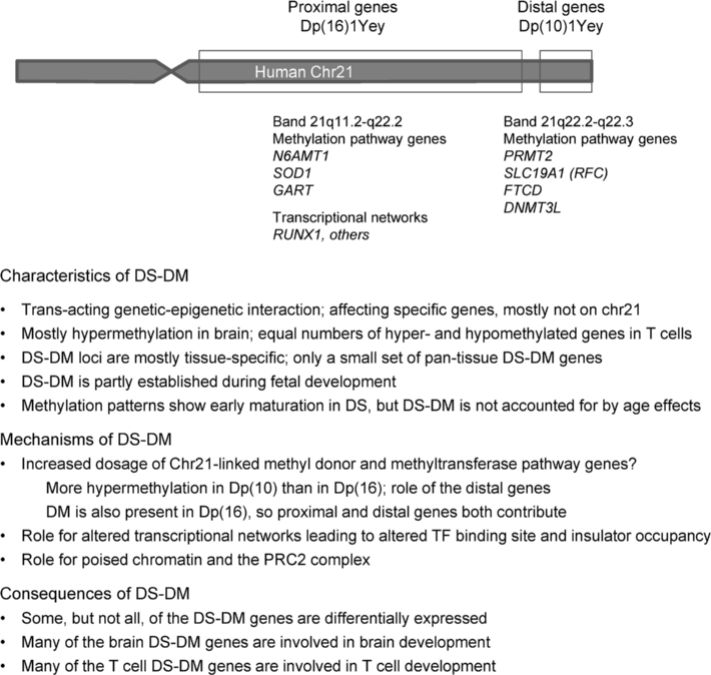
Summary of the main findings in this study. The known methylation pathway genes on chromosome 21 are taken from Blehaut et al. [106]. The small gap between the Dp(16) and Dp(10) regions represents the small part of human chromosome 21 that shows conserved synteny to mouse chromosome 17

In a broad perspective, our data begin to shed light on mechanisms leading to epigenetic changes downstream of chromosomal aneuploidies or duplications. In terms of timing, we have shown that the DS-DM begins early, already being partly established in fetal DS brains at mid-gestation. With regard to the *cis*- or *trans*-acting signals that might account for the observed patterns of DS-DM, while a genome-wide analysis of patterns of histone modifications in cells with Ts21 demonstrated low-level perturbations over large sub-chromosomal domains [88], the altered CpG methylation that we have described here is localized to specific regulatory sequences. Consistent with this finding of highly localized epigenetic changes, our results both in T cells and in brain cells suggest a role for altered transcriptional networks and altered TFBS occupancy downstream of Ts21 in shaping the patterns of DS-DM. In fetal brains the DS-DM occurs frequently in sequences that contain binding motifs for TFs that are essential for brain development, while in T cells the DS-DM loci show enrichment for TF binding sites involved in blood cell differentiation. Intriguingly, the highest enriched TFBS for T-cell DS-DM, the RUNX1 motif, is a binding site for the RUNX1 TF that is encoded on chromosome 21 and known to be over-expressed in DS. This situation supports a working hypothesis in which Ts21 leads to altered transcriptional networks that produce altered TFBS occupancies, which in turn lead to DS-DM (Fig. 6). Lastly, the DS-DM in glial cells occurs preferentially in promoters and enhancer regions that start development in a poised or “bivalent” chromatin state, as indicated by polycomb repressive marks overlapping with activating marks in embryonic stem cells, suggesting that the mechanism for gains of methylation in DS glia may have parallels with that in human gliomas [89, 90].

In addition to the altered TFBS occupancy model, a competing but not mutually exclusive hypothesis is one in which altered dosage of chromosome 21-linked epigenetic regulator genes, such as those encoding the DNA methyltransferase binding partner DNMT3L and folate pathway enzymes such as cystathionine beta-synthase (CBS) and the folinic acid transporter (SLC19A1), might play a role [91]. Over-expression of these genes might underlie the early maturation of methylation patterns that we have observed, and the trend toward hypermethylation in brain tissues. In this regard, a key finding in our study is that the epigenetic signature of human DS brain cells is partly but significantly recapitulated in brains from Dp(10)1Yey and Dp(16)1Yey mice. Of 175 conserved genes on human chromosome 21, a total of 41 are duplicated in Dp(10)1Yey and 115 are duplicated in Dp(16)1Yey. We have found here that the patterns of DM are not identical in these two mouse lines, but that both models show statistically significant similarities with human DS-DM, and that both show a trend toward *hyper*methylation of the DM genes, which is stronger in Dp(10) than in Dp(16). *Dnmt3l* and some of the Chr21-linked methyl donor pathway genes map to the Dp(10) duplicated region. However, since numerous DM loci were also detected in the Dp(16) brain, we conclude that multiple genes on chromosome 21, mapping both to the Dp(10) and to the Dp(16) syntenic regions, play a role in the phenomenon (Fig. 6). The Dp(10)1Yey and Dp(16)1Yey lines are elegant models in that they have precisely defined sub-chromosomal duplications, and in future work it should be possible to use combinations of these duplications with single gene knockouts to dissect the importance of specific chromosome 21-linked genes in producing the downstream epigenetic response. Other models, including mice with free chromosomal aneuploidies, not internal duplications, will also need to be examined.

Last but not least, although DS-DM is a consistent (recurrent) phenomenon in tissues of people with DS, there is intra-group variability among the DS cases (see the heat maps in Fig. 1). Given the well-known case-to-case variability in the severity of every DS-associated clinical phenotype, this intra-group variability in DS-DM is intriguing. Clinical correlations in large case–control series and experiments in the genetically manipulable mouse models will be needed to answer whether DS-DM contributes to, or alternatively partly ameliorates, the adverse effects of Ts21. Since we have shown here that the epigenetic signature of human DS brain cells is partly recapitulated in mouse models, such studies should now be feasible.

## Conclusions

These data from extensively validated high resolution profiling of differential CpG methylation in DS versus control brain cells and tissues and T lymphocytes include tissue-specificity of the DS-DM, onset of a component of DS-DM at the fetal stage, a contribution from 5hmC, and altered mRNA expression (DS-DE) of a subset of the DS-DM genes. Enrichment analysis shows that CpGs in specific classes of transcription factor binding sites, and CTCF sites, are preferentially affected, implicating altered binding site occupancy as a mechanism shaping the tissue-specific patterns of differential methylation. Whole genome bisulfite sequencing of brain DNA from mouse models of DS reveals alterations in CpG methylation patterns that partially but significantly parallel those in human DS brains and implicate multiple causal genes. These findings point to novel biological mechanisms in DS and have general implications for trans effects of chromosomal duplications and aneuploidies on epigenetic patterning.

## Materials and methods

### Adult and fetal brain tissues, isolation of neuronal and glial cell nuclei, and isolation of peripheral blood T cells

The tissue samples used in this study are listed in Table S1 in Additional file 2. Cryopreserved FC grey matter (Brodmann area BA9 or superior portion of BA10) from autopsies of 15 adult DS cases (seven females and eight males) and 12 controls (eight females and four males), as well as 13 DS cerebellar cortex samples (five females and eight males) and ten normal cerebellar cortex (five females and five males) from the same autopsy brains, were collected in the brain bank of the New York State Institute for Basic Research in Developmental Disabilities (NYSIBR). In addition, we utilized BA9 samples from three LOAD cases (three males) from the New York Brain Bank at Columbia University. From these brains, the samples used for each type of molecular analysis are in Table S1 in Additional file 2. For the FC samples, the median age was slightly higher in the control group (64 years in non-AD controls versus 57 years in the DS cohort), but the mean ages of the two groups were not significantly different (*p* = 0.15). The cerebellum cases and controls were likewise well matched for age (Table S1 in Additional file 2). Fetal brain whole cerebrum from DS cases (four females and four males) and controls (three females and three males) from mid-gestation terminations of pregnancy cryopreserved in the Biobank at the Institute of Child Health, University College of London were well matched for gestational age (median 15 weeks for DS and 14 weeks for controls; Table S1 in Additional file 2). In addition, there was no statistical difference in sex distribution in DS and control groups (Chi^2^*p* value > 0.5).

To obtain neuronal and glial cell nuclei from the adult DS and control brain tissues, 0.25 to 0.5 g of frozen tissue was homogenized on ice for 1 min. The tissue homogenate was then layered over a 60 % sucrose cushion and centrifuged at 28,500 rpm for 2.5 h at 4 °C, as described [92]. The pellet consisting of total cell nuclei was recovered and incubated with anti-NeuN-Alexa 488 conjugated antibody (Millipore MAB377X) for 1 h at 4 °C prior to FACS on a BD FACSAria cell sorter. Both neuronal enriched NeuN-positive and glial enriched NeuN-negative samples were recovered. Enrichment of NeuN-positive nuclei was confirmed (Fig. 1b) by using an aliquot post-FACS for cytospin preparations, which were visualized by immunofluorescence, with nuclei counterstained by TO-PRO-3 (Invitrogen). High molecular weight DNA was prepared from flow sorted nuclei by standard SDS/proteinase-K lysis followed by precipitation in 80 % isopropanol with glycogen as a carrier. CD3-positive T lymphocytes were isolated from peripheral blood of 11 DS adults and ten age-matched control adults, by antibody-based negative selection (RosetteSep T cell kit, Sigma). The male/female ratio was close to 50:50 and was the same for DS cases and controls (Table S1 in Additional file 2). Ts21 was confirmed by DNA copy number analysis (Figure S2 in Additional file 1) for all samples.

### DNA methylation profiling (5mC and 5hmC) on Illumina BeadChips

The amount and integrity of genomic DNA was assessed by gel electrophoresis and by PicoGreen® double-stranded DNA quantification assays (Life Technologies). DNA (500 ng) was bisulfite converted and analyzed according to the manufacturer’s instructions for Illumina 450K BeadChips, with all assays performed at the Roswell Park Cancer Institute Genomics Shared Resource. The BeadChip-based methylation assays entail bisulfite conversion of the genomic DNA followed by primer extensions to query the percentage methylation at each 485,000 (450 K) CpG dinucleotides, covering sequences in and around promoter-associated and non-promoter-associated CGIs, as well as many non-island promoter regions, associated with 99 % of RefSeq genes. Data were processed using Genome Studio software, which calculates the percentage methylation (AVG_Beta) at each CpG queried by the array, after background correction and normalization to internal controls. All probes mapping on the X or Y chromosome were removed along with those probes querying CpGs that overlapped common SNPs (with minor allele frequency ≥ 1 % in dbSNP build 138). Poorly performing probes with missing values (AVG_Beta detection *p* value > 0.05) in more than one sample per subgroup were filtered out. We corrected for batch effect in the filtered probe sets using the ComBat R package [93, 94], separately by tissue and cell type, including disease status in the adjustment model as an explanatory covariate. Batch effects were small overall and the batch correction had a modest effect on the DM gene lists; in comparing the lists of DM genes (FDR < 0.05 and absolute difference in mean AVG_Beta > 0.15) obtained with and without batch correction, ≥90 % of the genes detected as showing DM without batch correction were also detected after batch correction. The batch correction did, however, increase the number of genes and CpGs passing our statistical threshold by from 15–20 % in the several comparisons. To assess DNA copy number we used the intensity values from the 450K arrays, after normalization and model-based expression in dChip [95].

We further analyzed 12 DNA samples on the 450K BeadChips using an adaptation of the Illumina probe preparation protocol, in which the use of a modified bisulfite conversion procedure (BS/OXBS; Cambridge Epigenetics, CEGX), starting with 4 μg of genomic DNA, as the first step allows the relative contributions of 5mC and 5hmC to net methylation to be determined. This kit has not yet been fully optimized for compatibility with the Illumina arrays, leading to weaker signals. Nonetheless, when we filtered the resulting data by requiring that the probes pass a detection *p* value ≤ 0.005 in Genome Studio, and removing probes on the X and Y chromosomes and probes querying polymorphic CpGs, 380K useful probes remained.

### Standard bis-seq for 5mC and modified bis-seq for 5mC and 5hmC

Genomic DNA (500 ng) was bisulfite-converted using the EpiTect Bisulfite Kit (Qiagen). Sequences spanning the DM CpGs were amplified by PCR, using primers designed in MethPrimer [96]. Due to constraints in primer design, in one case the PCR amplicon used to validate an array finding was immediately adjacent to the DM probe (Illumina ID cg25468618); all other DM CpGs from the BeadChip data were included within the bis-seq amplicons. To evaluate the relative contributions of 5mC and 5hmC to DM at several loci, we used the TrueMethyl^TM^6 kit (CEGX), according to the instructions of the manufacturer, starting with 2 μg of DNA. This chemical conversion-based approach uses bis-seq of multiple clones to separately score 5mC-only and 5mC + 5hmC, so that the percentage contribution of 5hmC to net methylation at each CpG can be inferred from the difference. The PCR products were cloned using the TopoTA Cloning System (Invitrogen). For standard bis-seq at least ten independent clones were sequenced for a given amplicon and DNA sample, and for modified bis-seq we sequenced at least 14 clones for each amplicon and DNA sample under the 5mC-only and 5mC + 5hmC conditions. Primer sequences, amplicon characteristics and corresponding unconverted genomic sequences are in Table S16 in Additional file 2.

### Expression analysis of mRNA by Q-PCR

RNA was isolated using TRIZOL reagent (Invitrogen). In contrast to the intact genomic DNA, based on BioAnalyzer profiles the brain RNA samples showed moderate to severe degradation, with RNA integrity number (RIN) values ranging from 6 to 3. Total RNA was reverse transcribed with SuperScript® III First-Strand Synthesis Reverse Transcriptase (Life Sciences), with priming using a mixture of oligo-d(T) and random hexamers. Q-PCR was performed in triplicate in 96-well optical plates and repeated twice within independent cDNA sets. Each reaction contained 1× Power SYBR Green PCR master mix (Applied Biosystems) and 0.2 μM of each specific primer pair, which were designed using online Real Time PCR tool (IDT). Q-PCR was performed using a 7500 Fast Real-Time PCR System (Applied Biosystems), or a StepOnePlus instrument (BioRad), with an initial denaturation for 10 min at 95 °C, primer annealing at 50 °C for 2 min, followed by 40 cycles of 15 s at 95 °C and 1 min at 60 °C. The relative expression of target genes was calculated by the delta-CT method as described [97], with normalization using the *GAPDH* housekeeping gene, and these results were checked for consistency using a second housekeeping gene, *HPRT*. Despite the low RIN values, the average Ct values were <30 for each of the assayed genes using tenfold dilutions of the SuperScript-generated cDNA preparations, and the GAPDH and HPRT housekeeping gene controls showed Ct values <22 and <29 respectively, for all samples. Q-PCR primer sequences are in Table S17 in Additional file 2.

### PCA, identification of DS-DM loci, and tests for association of DS-DM with histone marks and DNA sequence motifs

Our strategy for bioinformatics is outlined in Figure S1a, b in Additional file 1. After filtering and batch effect correction, unsupervised hierarchical clustering, correlation heatmap, and PCA were performed on AVG_Beta (fractional methylation) values. Loci with recurrent DM were identified applying an absolute difference and Student’s t-test *p* value criteria (absolute methylation difference ≥ 0.15, unadjusted *p* value ≤ 0.001 corresponding to a FDR < 0.008, 0.041, 0.023, 0.055, and 0.031 for adult cerebellum, FC, glia, neurons, and T cells, respectively, and FDR < 0.27 for fetal FC; Tables S3–S7 in Additional file 2). Supervised hierarchical clustering using dChip [95], and subsequent enrichment analyses, were carried out using these thresholds. Multivariate linear regression was used to control for sex effects (Tables S3–S7 in Additional file 2). To ask whether the DM CpG sites were preferentially associated with specific types of histone modifications, we used data from ENCODE, as curated on the UCSC Genome Browser (http://www.genome.ucsc.edu/) [98, 99] and data from Zhu et al. [100] (GSM733758, GSE17312). Probes from the 450K arrays which overlapped with H3K4me1, H3K4me3, H3K27me3 and H3K27ac peaks in human embryonic stem cells (H1-hESC), human astrocytes (NH-A), human umbilical vein endothelial cells (HUVEC), and human neurons derived from H9-hESC were identified by their genomic positions. We categorized peaks as marking active promoters (H3K4me3 peaks alone), poised or “bivalent” promoters (overlapping H3K4me3 and H3K27me3 peaks), active enhancers (overlapping H3K4me1 and H3K27ac peaks), intermediate enhancers (H3K4me1 peaks alone), and poised enhancers (overlapping H3K4me1 and H3K27me3 peaks) as described by Zentner et al. [101]. Enrichment of each regulatory element in DM loci compared with non-DM loci was assessed using hypergeometric tests. All analyses were performed using STATA v.12 software (StataCorp, LP). To determine whether the DM CpG sites were preferentially associated with specific TFBS motifs, we used the Hypergeometric Optimization of Motif Enrichment (HOMER) motif discovery tool (http://biowhat.ucsd.edu/homer/ngs/index.html) and performed de novo motif searches with the default settings: background selection matched on percentage GC content, percentage GC content normalization, oligo auto-normalization, regions for motif finding set as 200-bp windows centered on CpG sites. The background was calculated in 200-bp windows centered on all CpGs queried by the 450K arrays, excluding CpGs on chromosome X or Y and mapping common SNPs. Motif enrichment (target versus background) was calculated using the cumulative binomial distribution. Best match to JASPAR TF motifs was kept only if the alignment score was >0.6. When the same DS-DM loci accounted for several de novo motifs, only the strongest motif was kept. De novo motif instances were then overlapped with TF ChIP seq peaks (data from the ENCODE project) and logistic regression was used to determine the strongest enriched TF peak for each de novo motif. To assess enrichment for known TF motifs among DS-DM, known TF motifs (ENCODE) within a 200-bp window centered on each CpG were identified and logistic regression was carried out.

### GSEA and gene-annotation enrichment analysis

GSEA was carried out using the Broad Institute GseaPreRank tool [102]. DM genes were ranked by t-test *p* values; for genes covered by multiple probes, the lowest t-test was used. The GSEA procedure on ranked gene lists [103] was used to compute normalized enrichment score and FDR. We tested all sets from C2 (Curated) and C5 (Gene Ontology) collections of the Molecular Signature Database (MsigDB, v.4.0), excluding gene sets smaller than 15 and larger than 1000. The significance level of FDR q-values was set at 0.05. Gene annotation enrichment was assessed by overlapping the DM gene list with the Uniprot tissue annotation and Gene Ontology (GOTERM_BP_FAT, GOTERM_CC_FAT, GOTERM_MF_FAT) database using the Database for Annotation, Visualization and Integrated Discovery (DAVID v.6.7; http://david.abcc.ncifcrf.gov) functional annotation tool [104]. Fisher exact tests were corrected for multiple tests using the Benjamini-Hochberg method [105].

### Tests for rate of maturation of methylation patterns and rate of methylation aging

Using univariate analysis, we first identified two sets of loci with developmental stage or age-dependent methylation in control brains and T cells, i.e., CpGs with significant differences in methylation within the fetal stage of development or within adult aging, respectively. A significant effect was defined as age *p* value < 0.05, methylation changes > 0.1 per 10 weeks, and adjusted R squared > 0.8 in fetal tissues. In adult tissues, methylation changes were more subtle and we defined significance as age *p* value < 0.05, and adjusted R squared > 0.8. Using these panels of CpGs as an indicator of epigenetic maturation and aging in brain and T cells, we tested for an effect of DS status. After averaging fractional methylation across these age-dependent CpGs, we carried out multivariate linear regressions including fractional methylation as response variable and disease status, age, and the interaction term between disease status and age as explanatory covariates. We also performed multivariate linear regressions on each CpG, modeling the same covariates. In these models, the disease status term reflects the difference at baseline while the interaction term reflects the longitudinal effect of the disease across age or developmental stage. To assess differential aging effects between DS and controls, CpGs with a differential aging effect were defined for fetal data by adjusted R squared > 0.8, age *p* value < 0.05, interaction *p* value < 0.05, and differential aging effect > 0.1 per 10 weeks, and for adult data by adjusted R squared > 0.8, age *p* value < 0.05, interaction *p* value < 0.05. Graphing and linear regression estimating the overall differential aging effects was carried out by averaging the fractional methylation across these CpGs.

### Tests for correlations of DS-DM with tissue-specific mRNA expression

Expression data from human FC (superior frontal gyrus) were downloaded from the Allen Brain Atlas and averaged over six normal brains. A Z score (−μ/sd) was calculated for each probe, and expression at the gene level was calculated by the median of the probes mapping to the same gene. To compare gene expression between brain and other tissues, data from BioGPS [25] were used after Z score transformation to allow comparison between arrays. We classified expression into four patterns: (i) genes with brain-specific expression (Z-score ≥ 0.5 in the brain only or in less than two additional tissues), (ii) genes with brain-specific repression (Z-score ≤ −0.5 in the brain only or in less than two additional tissues), (iii) genes with other tissue-specific expression or repression (Z-score ≥ 0.5 or ≤ −0.5 in less than three tissues, and not in brain), and (iv) genes with a multi-tissue or pan-tissue expression pattern (Z-score ≥ 0.5 or ≤ −0.5 in three or more tissues). The distribution of these patterns within the hypermethylated gene sets from our 450K data was compared with the expected distribution using a Fischer exact test. The expected distribution was estimated from a set of 15,213 genes for which expression data could be obtained from both Allen Brain Atlas and BioGPS. For analyzing enrichment of the DS-DM genes with regard to developmental stage-specific expression, data generated using the ABI Human Genome array from three technical replicates of a normal fetal brain and three technical replicates of a normal adult brain were downloaded from the National Center for Biotechnology Information (NCBI; http://www.ncbi.nlm.nih.gov) Gene Expression Omnibus (GEO) database (dataset GDS3011_GSE7905). For genes queried by several probes, the averaged values were used. Expression was considered higher in fetal or adult brain when *p* ≤ 0.05 using the t-test. The proportion of genes with higher expression in fetal brain in our glial and neuron DM gene sets was compared with the expected values using the hypergeometric test. The expected distribution was estimated from a set of 14,496 genes for which both expression and methylation data were available.

### 5aza-dC treatment of human astrocytes

Low-passage normal human astrocytes (Clonetics-Lonza) were purchased and cultured as per the manufacturer’s instructions. Cells were treated with varying concentrations of 5aza-dC, added at day 0 and day 3, for four days prior to harvesting for RNA isolation.

### WGBS for assessment of differential CpG methylation patterns in chromosome-engineered mouse models of DS

Genomic DNA (500 ng) from two chromosome-engineered lines that are models of DS, namely B6;129S7-Dp(16Lipi-Zbtb21)1Yey (abbreviated as Dp(16)1Yey) and B6;129S7-Dp(10Prmt2-Pdxk)2Yey (abbreviated as Dp(10)1Yey), after backcrossing to B6 for four generations, as well as a normal (wt) littermate, was bisulfite-converted using the EpiTect Bisulfite Kit (Qiagen) followed by Nextgen sequencing (Illumina HiSEQ; 100-bp paired-end sequencing). After trimming of adaptor sequences and filtering out low quality score (<30) reads using TrimGalore, Methyl-Seq reads were aligned against the mouse genome (Mm10) using Bismark v.0.8.2. Duplicated reads were removed using Samtools (Samtools-0.1.19), and methylation calling was carried out using Bismark methyl extractor. After filtering, there were ≥1005 million mapable reads from autosomes in each of the three samples, corresponding to a median coverage ≥28×. CpGs with less than 20× coverage were filtered out as suboptimal for assessing DM. To confirm trisomy in the regions spanning chr10:76207226–78464948 in Dp(10)1Yey and chr16:75540514–97962621 in Dp(16)1Yey, we computed the ratio of the coverage between trisomic and wt littermate mice, after normalization by the total number of reads mapping to chromosomes 10 and 16, respectively.

Methylation differences between Dp(10)1Yey and wt mice, as well as Dp(16)1Yey and wt mice were calculated at the CpG level. For comparing CpG methylation patterns between human and mouse, we first defined genomic windows of 1 kb around all positions in the mouse genome that matched that of the human CpGs from the 450K arrays. For this purpose, Illumina queried CpGs coordinates were converted to mouse genome (mm10) using the LiftOver tool. Next, at low stringency we defined DM in the mouse models as at least one CpG with a >15 % difference in fractional methylation compared with wt, and at least two other DM CpGs with the same direction of methylation change in the same 1-kb window. As a higher stringency definition of DM in a given window we additionally required at least one CpG in the window to show a statistically significant difference in methylation, compared with that CpG in wt, by Fisher’s exact test. This statistical approach is sensitive not only to the magnitude and consistency of the differences in methylation in a given window, but also to the depth of sequencing (number of reads), and hence the confidence, at each CpG position. To assess the enrichment for human DS-DM gene orthologues in the mouse data, the percentage of overlapping human and mouse genes with same direction of methylation changes was calculated for increasingly stringent sets of mouse DM genes. Significance of the enrichment was determined using hypergeometric tests.

### Data availability

The human and mouse methylation data sets are available at NCBI’s GEO database (human 450K methylation [GEO:GSE74486], mouse WGBS [GEO:GSE74505], super-series dataset for human and mouse [GEO:GSE74519]).

### Ethics

The human study was approved by the Institutional Review Board of John Hopkins School of Medicine (Protocol #NA_00039482). All blood samples were taken in accordance with the Helsinki Declaration, with informed consent or assent, as specified in the above Institutional Review Board protocol. The mouse study was approved by the Institutional Animal Care and Use Committee of Roswell Park Cancer Institute (Protocol #928M).

## Additional files

Additional file 1:
**Supplemental figures.** (ZIP 1688 kb) Additional file 2:
**Supplemental tables.** (ZIP 4474 kb) Additional file 3:
**Supplemental bioinformatics.** (PDF 155 kb)

## Abbreviations

5aza-dC: 5aza-deoxycytidine
AD: Alzheimer’s disease
bis-seq: bisulfite sequencing
bp: base pair
CGI: CpG island
DM: differential methylation
DS: Down syndrome
DS-DM: Down syndrome-specific differential methylation
FACS: fluorescence-activated cell sorting
FC: frontal cortex
FDR: false discovery rate
GEO: Gene Expression Omnibus
GSEA: gene set enrichment analysis
LOAD: late-onset Alzheimer’s disease
NCBI: National Center for Biotechnology Information
PCA: principal component analysis
PRC: polycomb repressive complex
Q-PCR: quantitative PCR
RIN: RNA integrity number
SNP: single nucleotide polymorphism
TF: transcription factor
TFBS: transcription factor binding site
Ts21: trisomy 21
WGBS: whole genome bisulfite sequencing
wt: wild type
