# Erratum to: Trans effects of chromosome aneuploidies on DNA methylation patterns in human Down syndrome and mouse models

**DOI:** 10.1186/s13059-016-0949-5

**Published:** 2016-06-09

**Authors:** Maite Mendioroz, Catherine Do, Xiaoling Jiang, Chunhong Liu, Huferesh K. Darbary, Charles F. Lang, John Lin, Anna Thomas, Sayeda Abu-Amero, Philip Stanier, Alexis Temkin, Alexander Yale, Meng-Min Liu, Yang Li, Martha Salas, Kristi Kerkel, George Capone, Wayne Silverman, Y. Eugene Yu, Gudrun Moore, Jerzy Wegiel, Benjamin Tycko

**Affiliations:** Taub Institute for Research on Alzheimer’s Disease and the Aging Brain and Institute for Cancer Genetics, Columbia University Medical Center, New York, NY 10032 USA; The Children’s Guild Foundation Down Syndrome Research Program, Genetics Program and Department of Cancer Genetics, Roswell Park Cancer Institute, Buffalo, NY 14263 USA; Fetal Growth and Development Group, Clinical and Molecular Genetics Unit, UCL Institute of Child Health, London, WC1N 1EH UK; Department of Psychology, Kennedy Kreiger Institute, John Hopkins University School of Medicine, Baltimore, MD 21205 USA; Department of Developmental Neurobiology, New York State Institute for Basic Research in Developmental Disabilities, Staten Island, NY 10314 USA; Department of Pathology and Cell Biology, Columbia University Medical Center, New York, NY 10032 USA

After the publication of this work [1] an error was noted in the ethics statement where part of the text was missing.

This has now been updated in the original version of this article.
