# Supplementary material for: Trans effects of chromosome aneuploidies on DNA methylation patterns in human Down syndrome and mouse models
Source: Genome Biol. 2015 Nov 25;16:263. doi: 10.1186/s13059-015-0827-6 (PMC4659173; doi:10.1186/s13059-015-0827-6)

Suppl. Fig. S1A

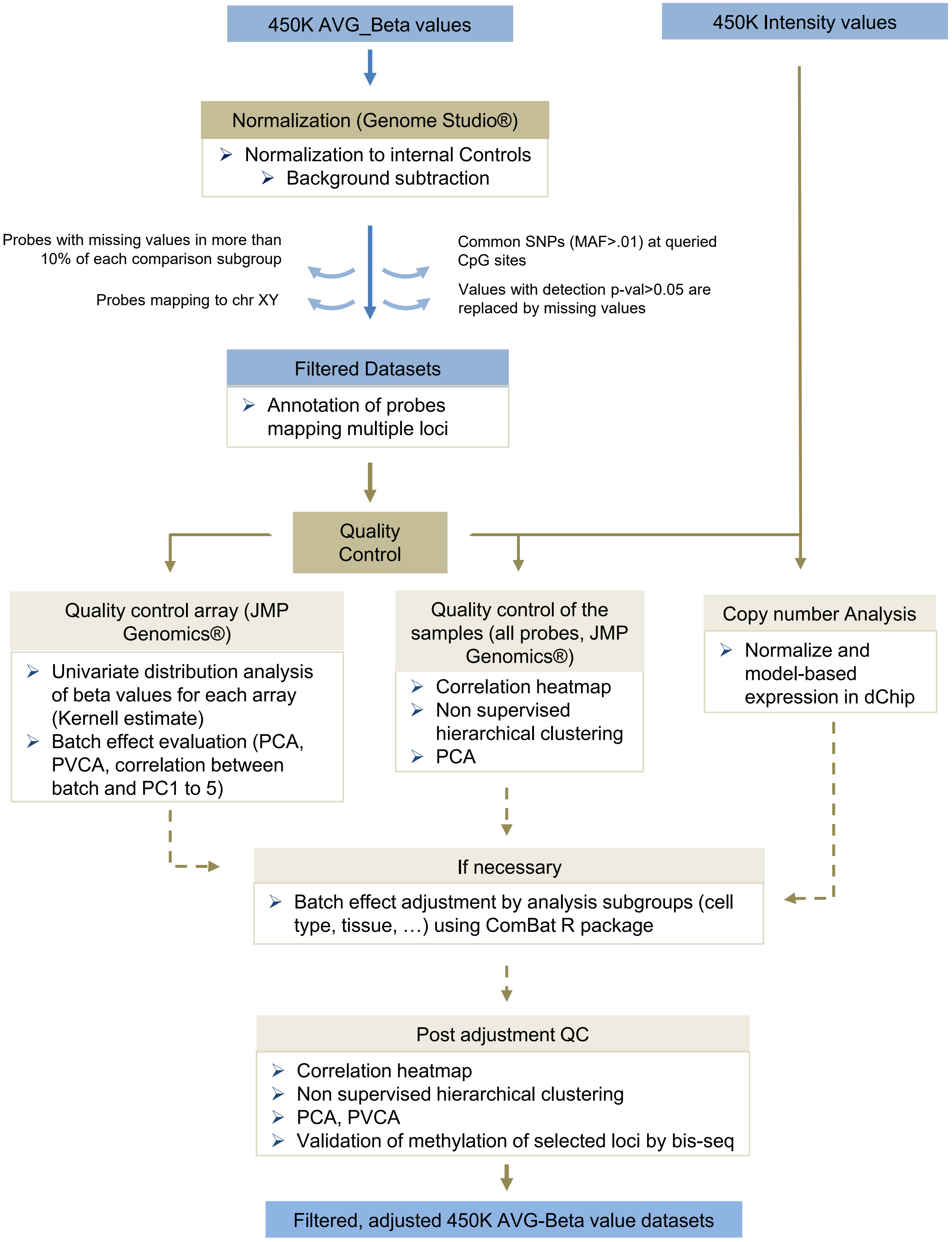

PCA: Principal Component Analysis; PVCA Principal Variance Component Analysis

Suppl. Fig. S1B

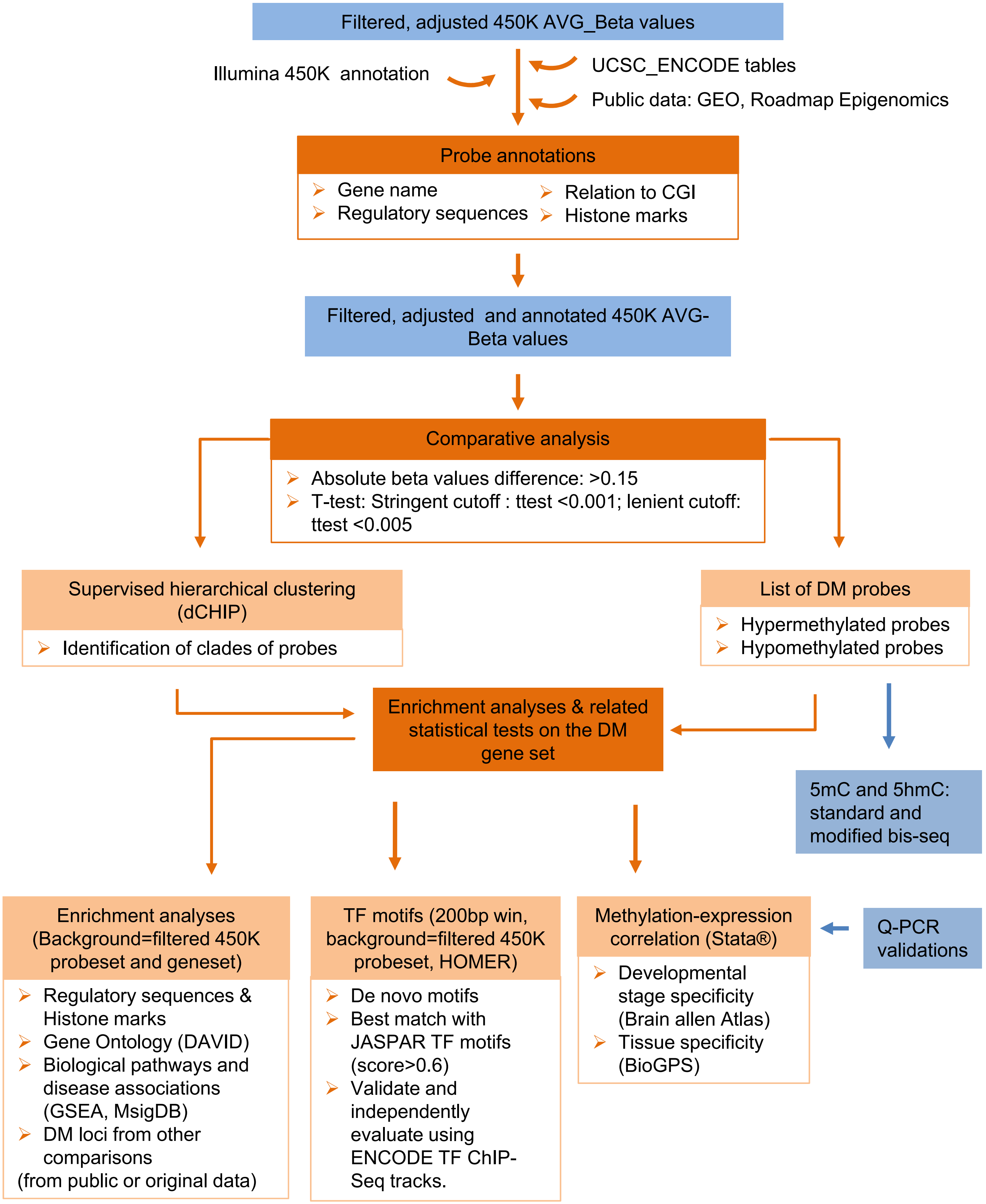

Suppl. Fig. S2

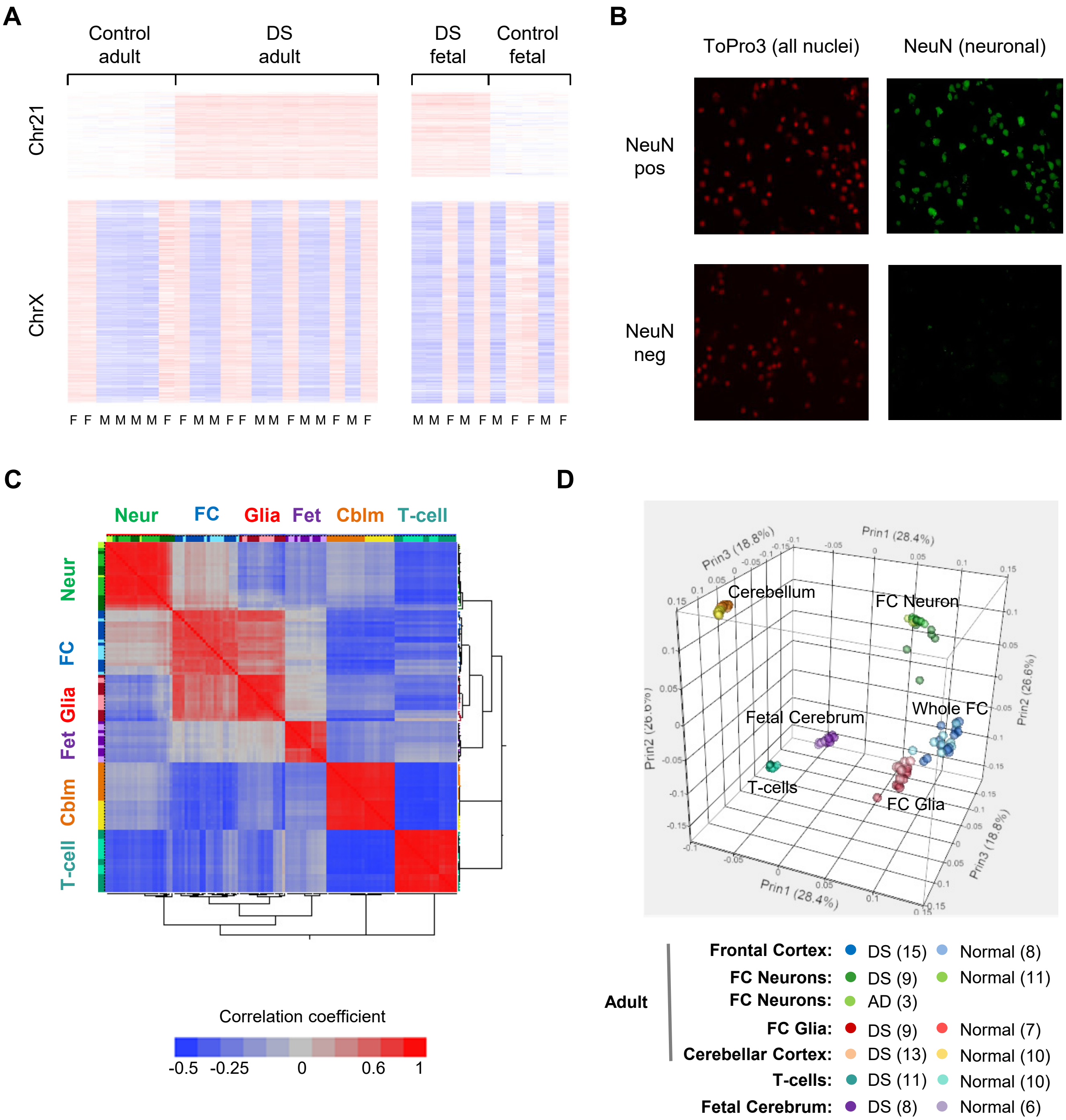
A
Control adult
DS adult
DS fetal
Control fetal
Chr21
ChrX
F F M M M M F F M M F F M M F M M F M F
M M F M F M F F M F
B
ToPro3 (all nuclei)
NeuN (neuronal)
NeuN pos
NeuN neg
C
Neur
FC
Glia
Fet
Cblm
T-cell
Correlation coefficient
-0.5
-0.25
0
0.6
1
D
Cerebellum
FC Neuron
Fetal Cerebrum
Whole FC
T-cells
FC Glia
Prin1 (28.4%)
Prin2 (26.6%)
Prin3 (18.8%)
Adult
Frontal Cortex: DS (15) Normal (8)
FC Neurons: DS (9) Normal (11)
FC Neurons: AD (3)
FC Glia: DS (9) Normal (7)
Cerebellar Cortex: DS (13) Normal (10)
T-cells: DS (11) Normal (10)
Fetal Cerebrum: DS (8) Normal (6)

Suppl. Fig. S3

A

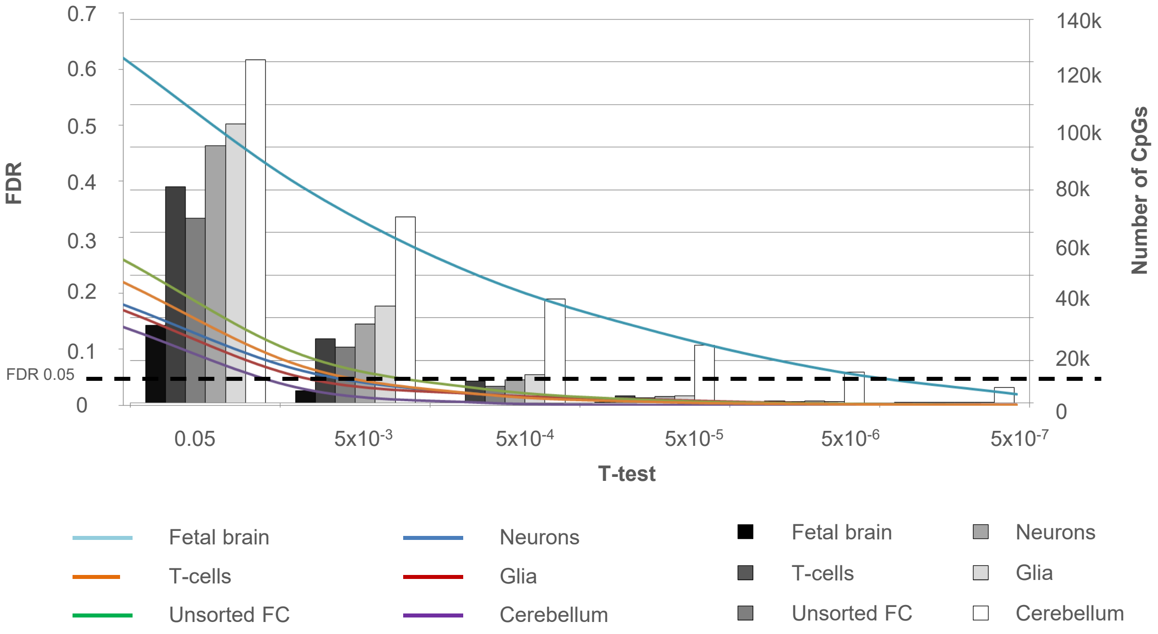

B

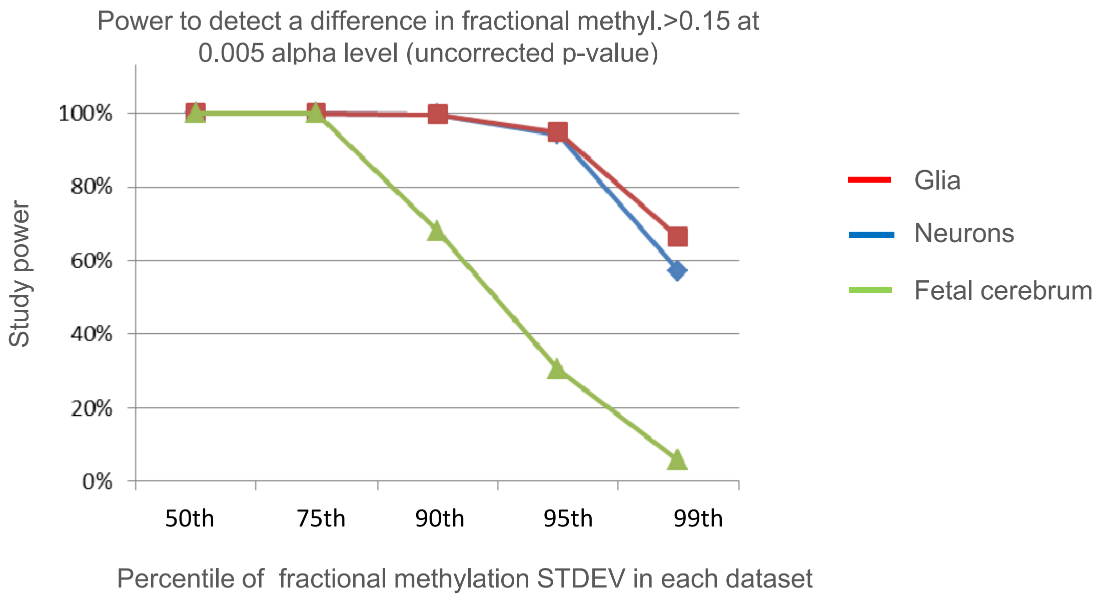

Suppl. Fig. S4

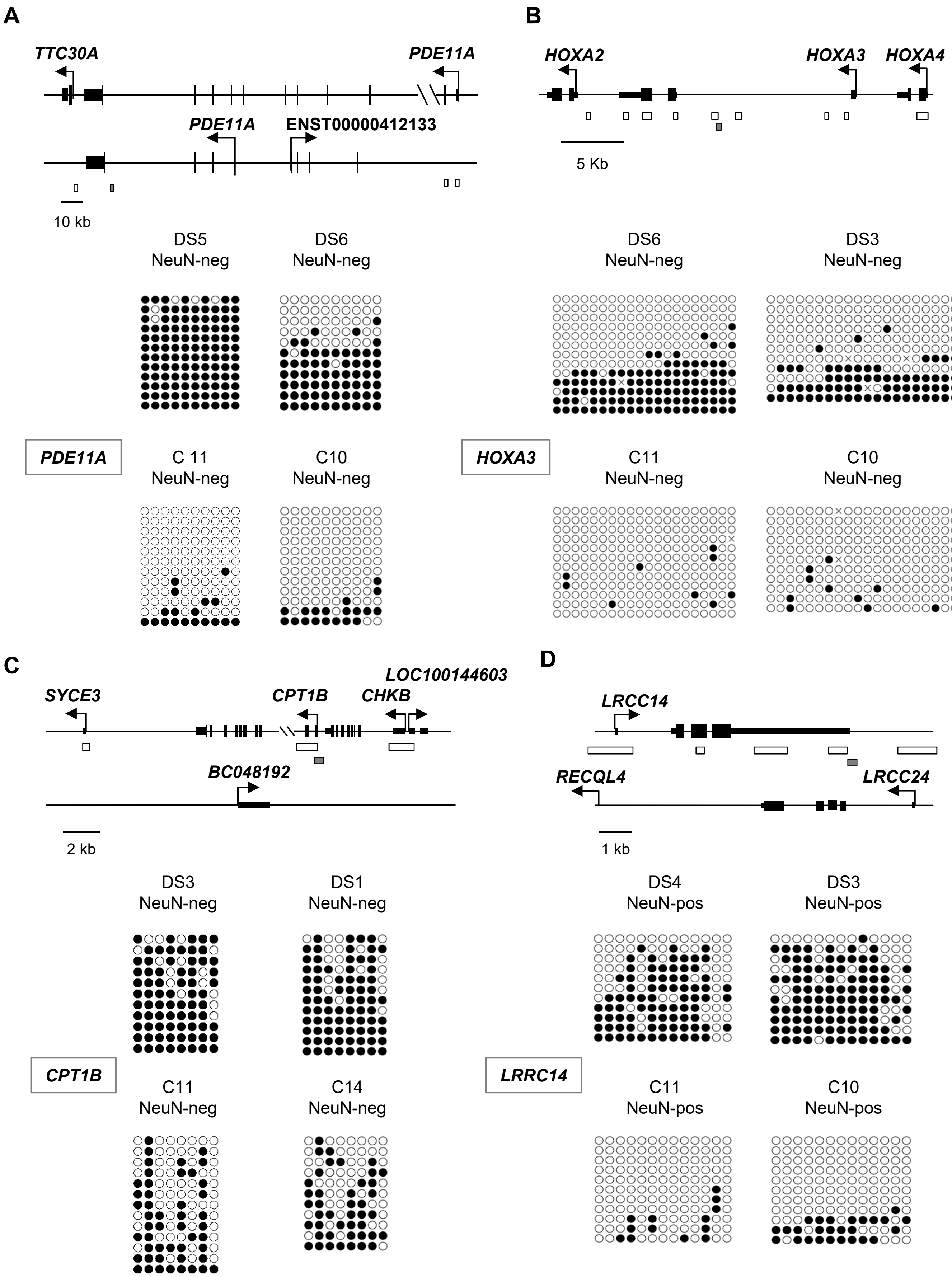
A
TTC30A
PDE11A
PDE11A
ENST00000412133
10 kb
DS5
NeuN-neg
DS6
NeuN-neg
PDE11A
C 11
NeuN-neg
C10
NeuN-neg
B
HOXA2
HOXA3
HOXA4
5 Kb
DS6
NeuN-neg
DS3
NeuN-neg
HOXA3
C11
NeuN-neg
C10
NeuN-neg
C
SYCE3
CPT1B
CHKB
LOC100144603
BC048192
2 kb
DS3
NeuN-neg
DS1
NeuN-neg
CPT1B
C11
NeuN-neg
C14
NeuN-neg
D
LRCC14
RECQL4
LRCC24
1 kb
DS4
NeuN-pos
DS3
NeuN-pos
LRRC14
C11
NeuN-pos
C10
NeuN-pos

Suppl. Fig. S5

A

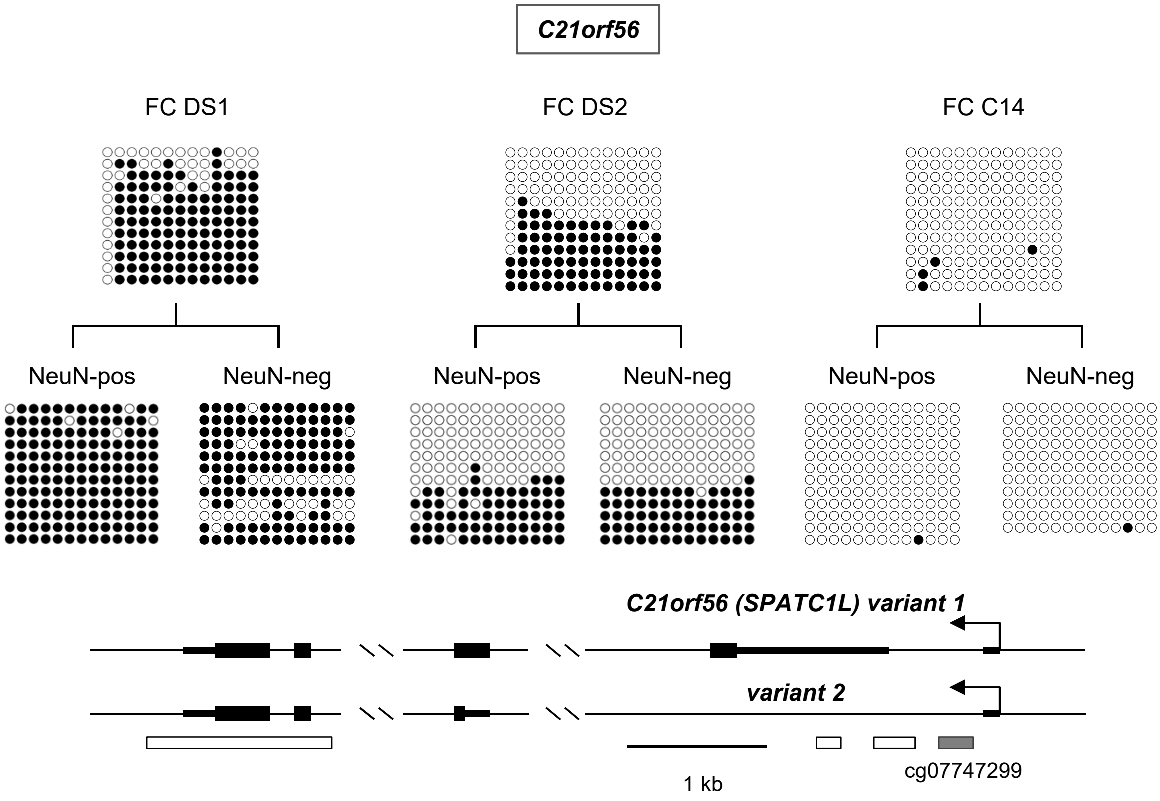

B

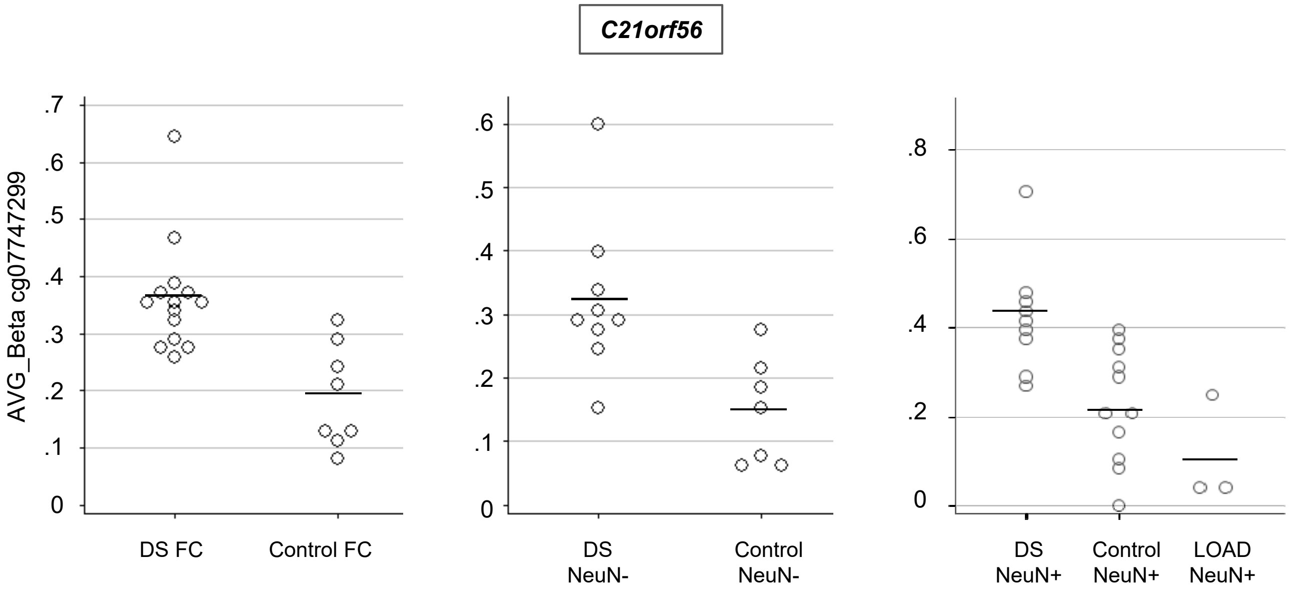

Suppl. Fig. S6

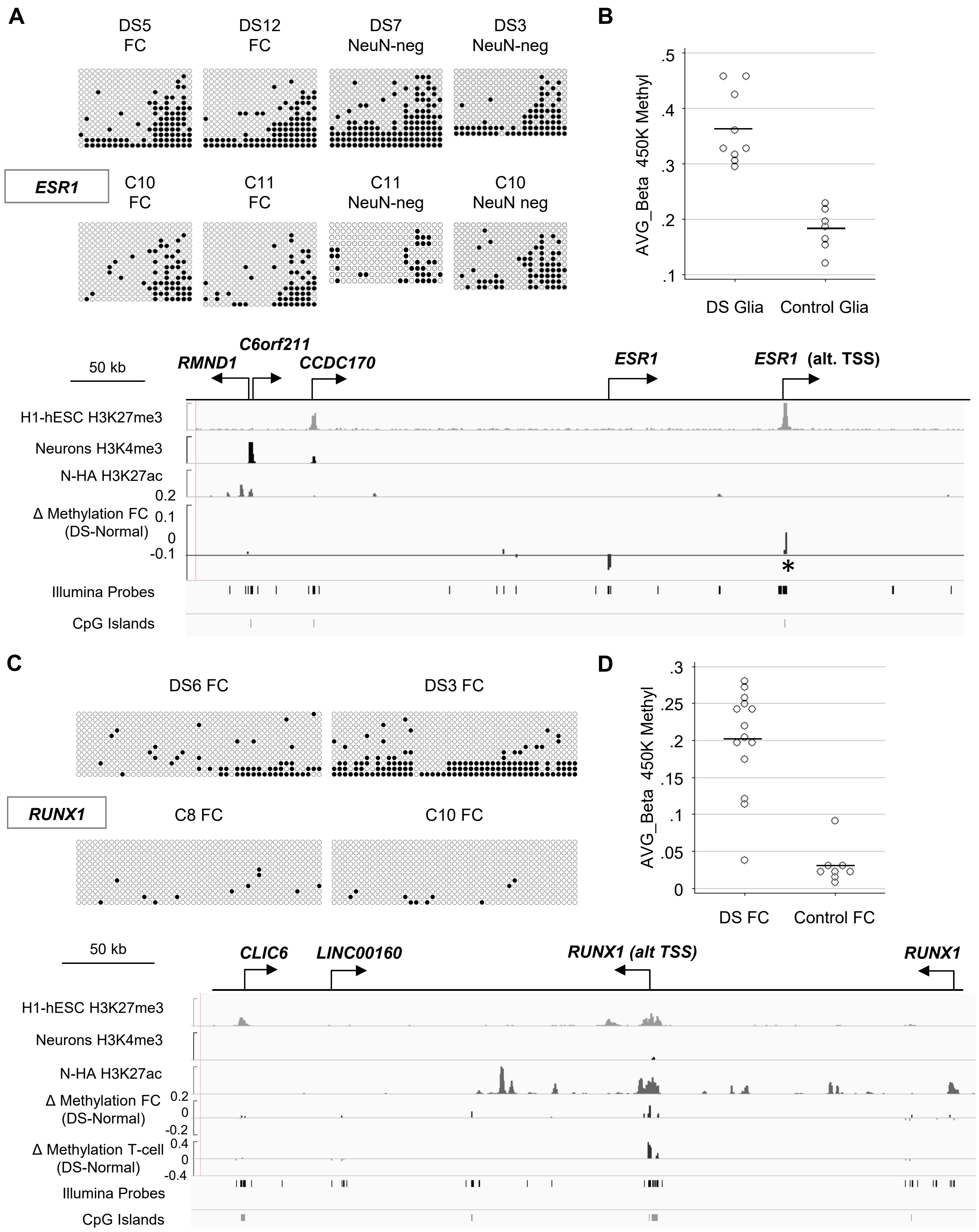

A
DS5 FC
DS12 FC
DS7 NeuN-neg
DS3 NeuN-neg
ESR1
C10 FC
C11 FC
C11 NeuN-neg
C10 NeuN neg
B
AVG_Beta 450K Methyl
.5
.4
.3
.2
.1
DS Glia
Control Glia
50 kb
RMND1
C6orf211
CCDC170
ESR1
ESR1 (alt. TSS)
H1-hESC H3K27me3
Neurons H3K4me3
N-HA H3K27ac
Δ Methylation FC (DS-Normal)
0.2
0.1
0
-0.1
*
Illumina Probes
CpG Islands
C
DS6 FC
DS3 FC
RUNX1
C8 FC
C10 FC
D
AVG_Beta 450K Methyl
.3
.25
.2
.15
.1
.05
0
DS FC
Control FC
50 kb
CLIC6
LINC00160
RUNX1 (alt TSS)
RUNX1
H1-hESC H3K27me3
Neurons H3K4me3
N-HA H3K27ac
Δ Methylation FC (DS-Normal)
0.2
0
-0.2
Δ Methylation T-cell (DS-Normal)
0.4
0
-0.4
Illumina Probes
CpG Islands

Suppl. Fig. S7

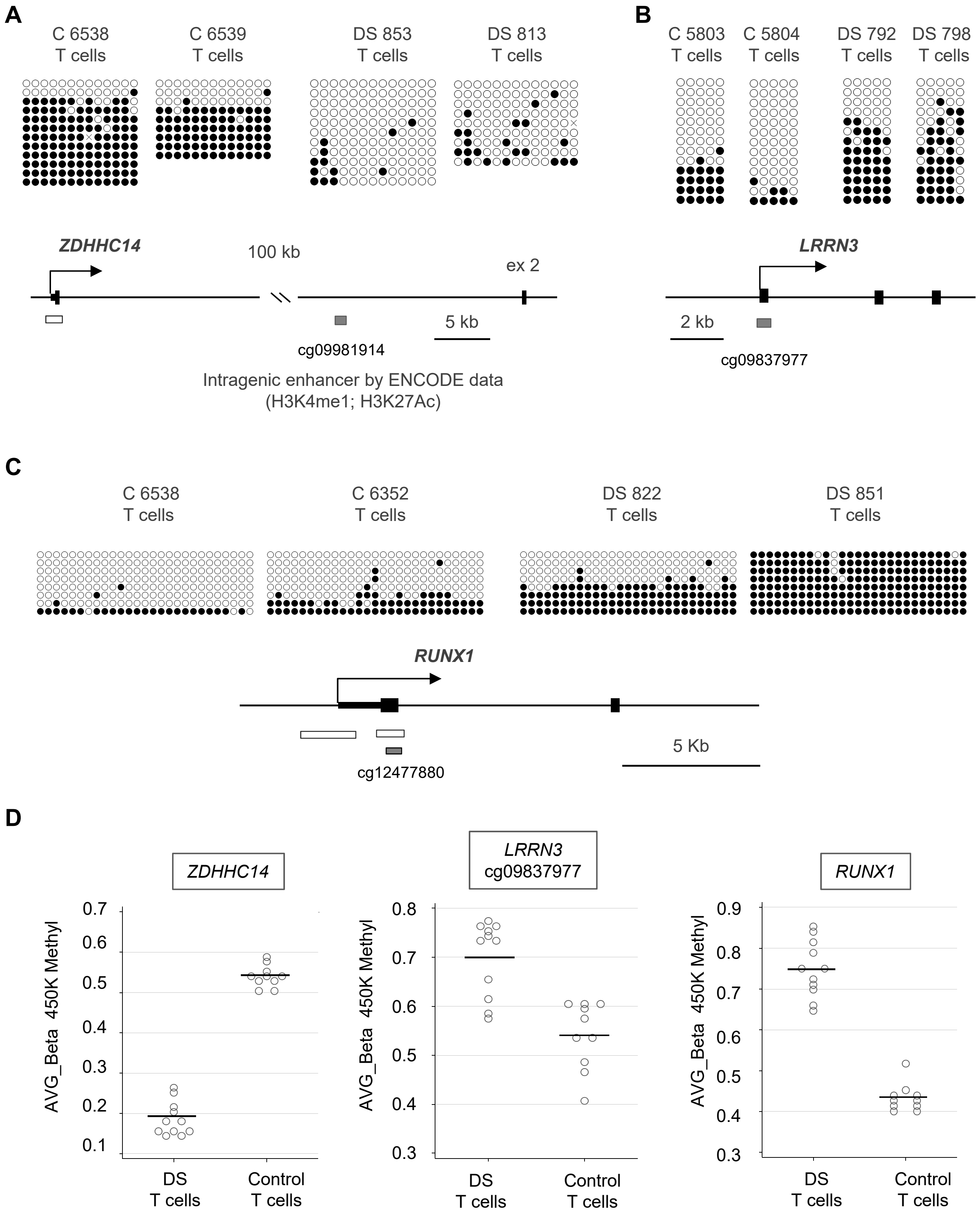
A
C 6538
T cells
C 6539
T cells
DS 853
T cells
DS 813
T cells
ZDHHC14
100 kb
ex 2
5 kb
cg09981914
Intragenic enhancer by ENCODE data
(H3K4me1; H3K27Ac)
B
C 5803
T cells
C 5804
T cells
DS 792
T cells
DS 798
T cells
LRRN3
2 kb
cg09837977
C
C 6538
T cells
C 6352
T cells
DS 822
T cells
DS 851
T cells
RUNX1
5 Kb
cg12477880
D
ZDHHC14
LRRN3
cg09837977
RUNX1
AVG_Beta 450K Methyl
0.1
0.2
0.3
0.4
0.5
0.6
0.7
0.8
0.9
DS
T cells
Control
T cells

Suppl. Fig. S8

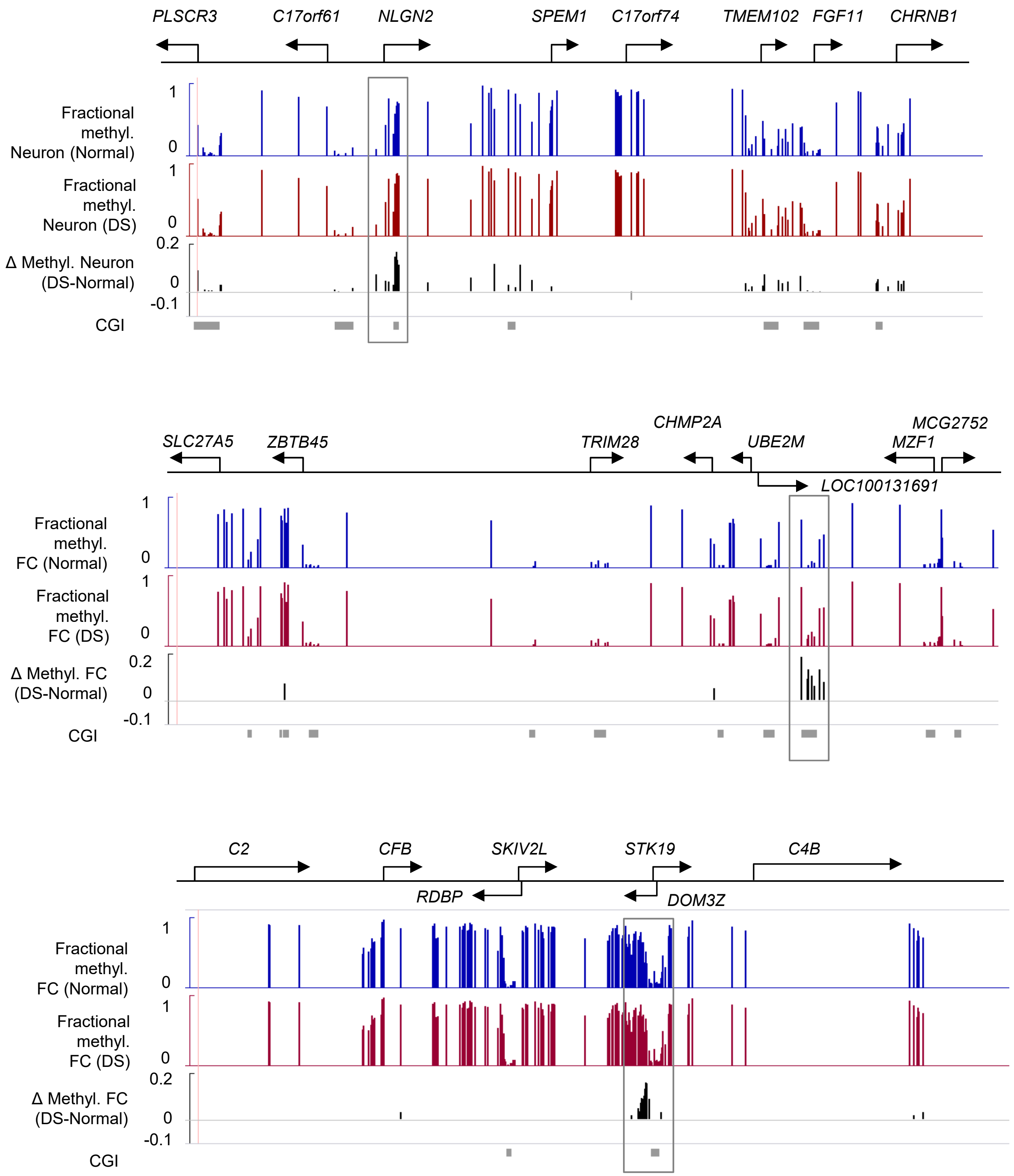
PLSCR3
C17orf61
NLGN2
SPEM1
C17orf74
TMEM102
FGF11
CHRNB1
Fractional methyl. Neuron (Normal)
Fractional methyl. Neuron (DS)
Δ Methyl. Neuron (DS-Normal)
CGI
1
0
0.2
-0.1
SLC27A5
ZBTB45
TRIM28
CHMP2A
UBE2M
MCG2752
MZF1
LOC100131691
Fractional methyl. FC (Normal)
Fractional methyl. FC (DS)
Δ Methyl. FC (DS-Normal)
C2
CFB
SKIV2L
STK19
C4B
RDBP
DOM3Z

Suppl. Fig. S9

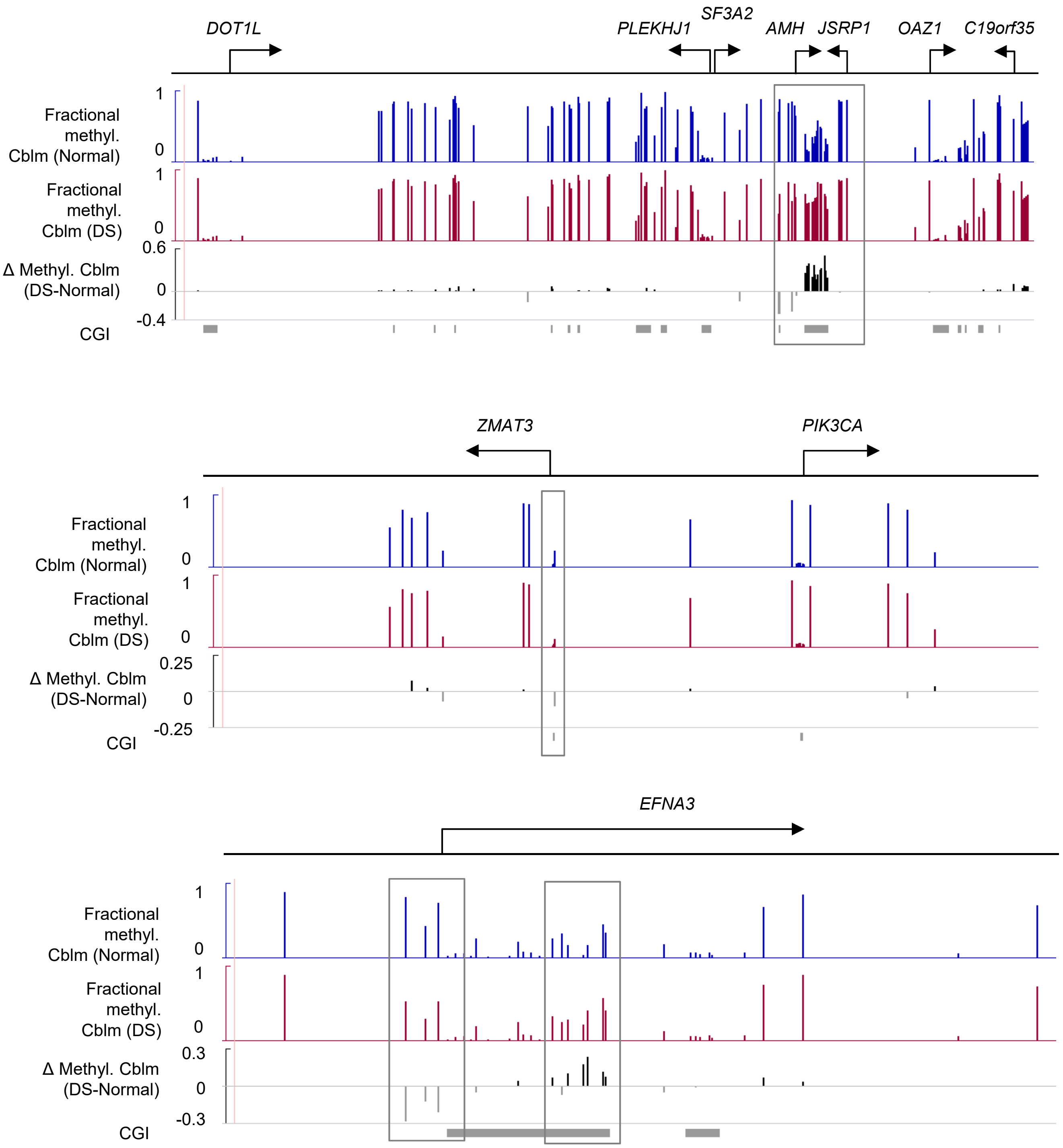
DOT1L
PLEKHJ1
SF3A2
AMH
JSRP1
OAZ1
C19orf35
Fractional methyl. Cblm (Normal)
Fractional methyl. Cblm (DS)
Δ Methyl. Cblm (DS-Normal)
CGI
1
0
0.6
-0.4
ZMAT3
PIK3CA
0.25
-0.25
EFNA3
0.3
-0.3

Suppl. Fig. S10

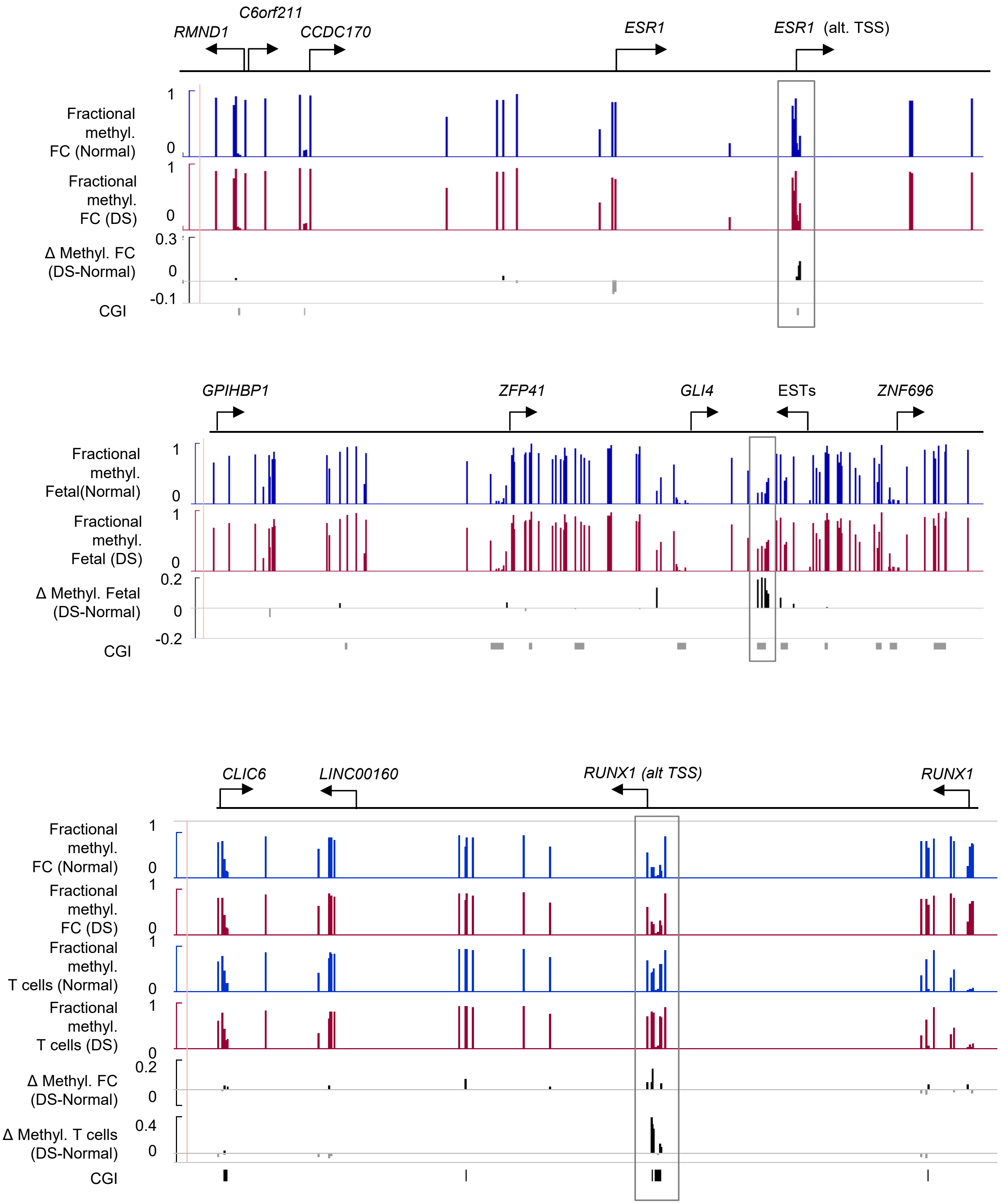

Suppl. Fig. S11

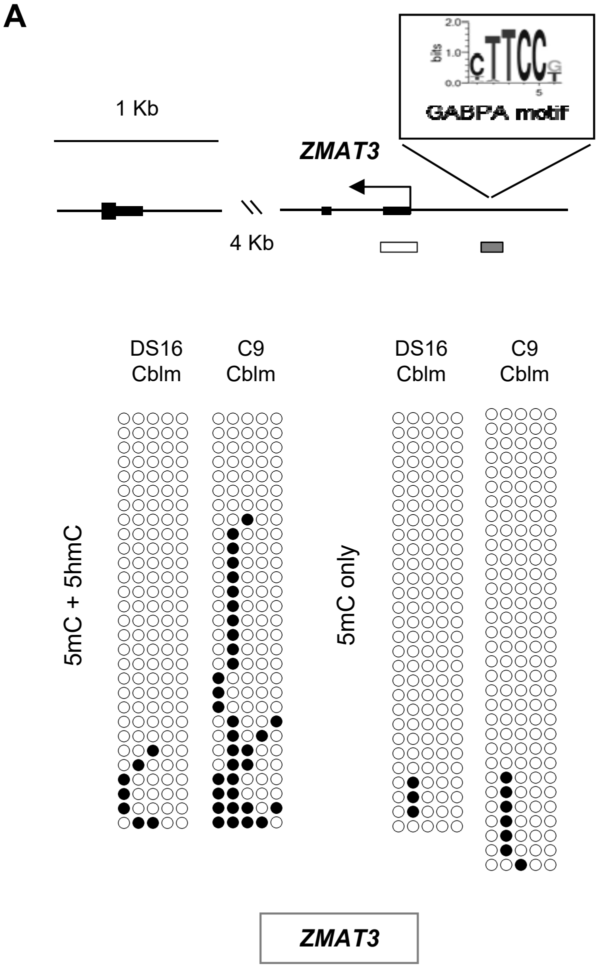

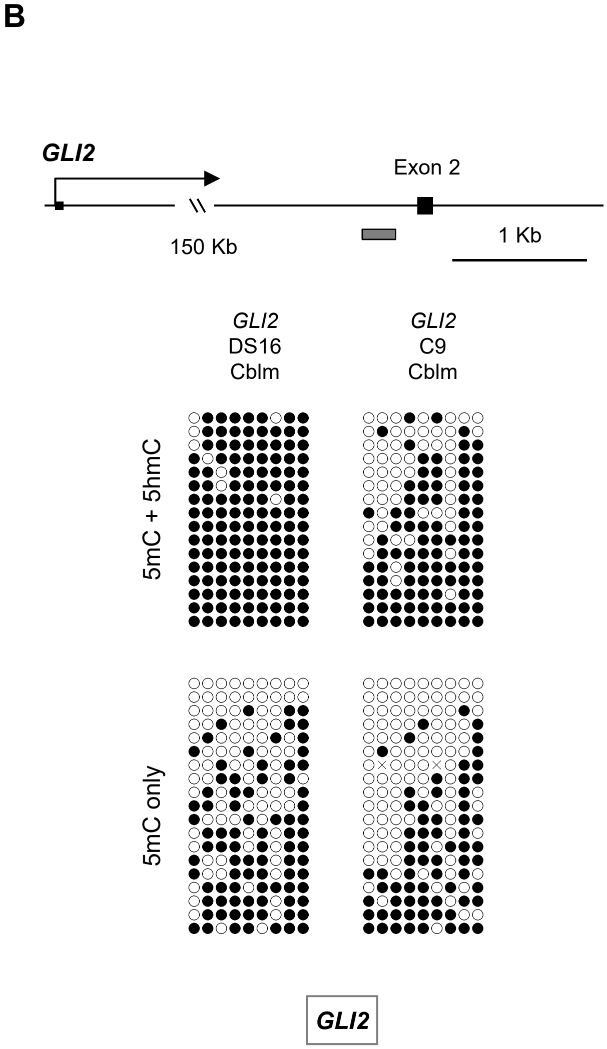

Suppl. Fig. S12

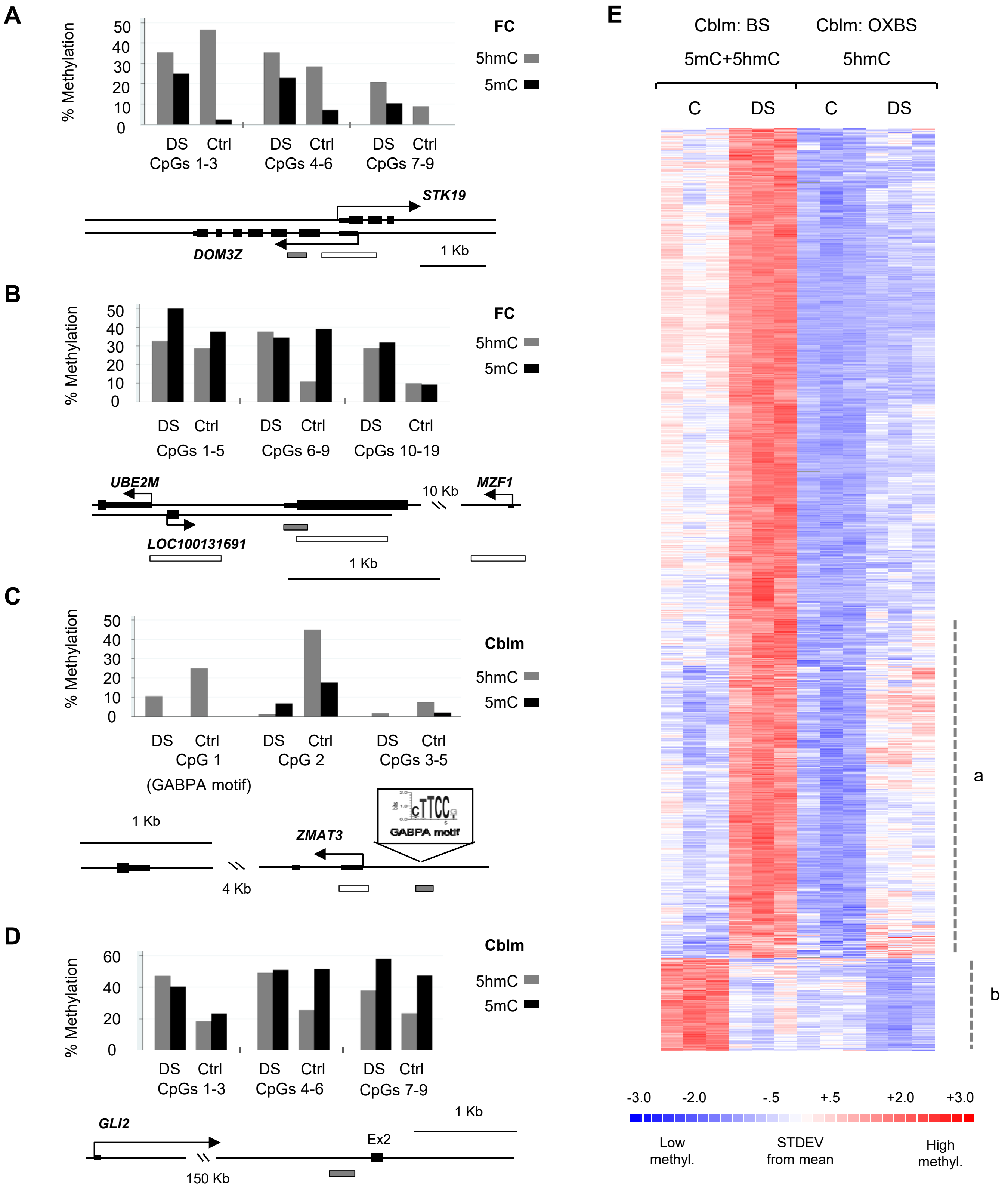

Suppl. Fig. S13

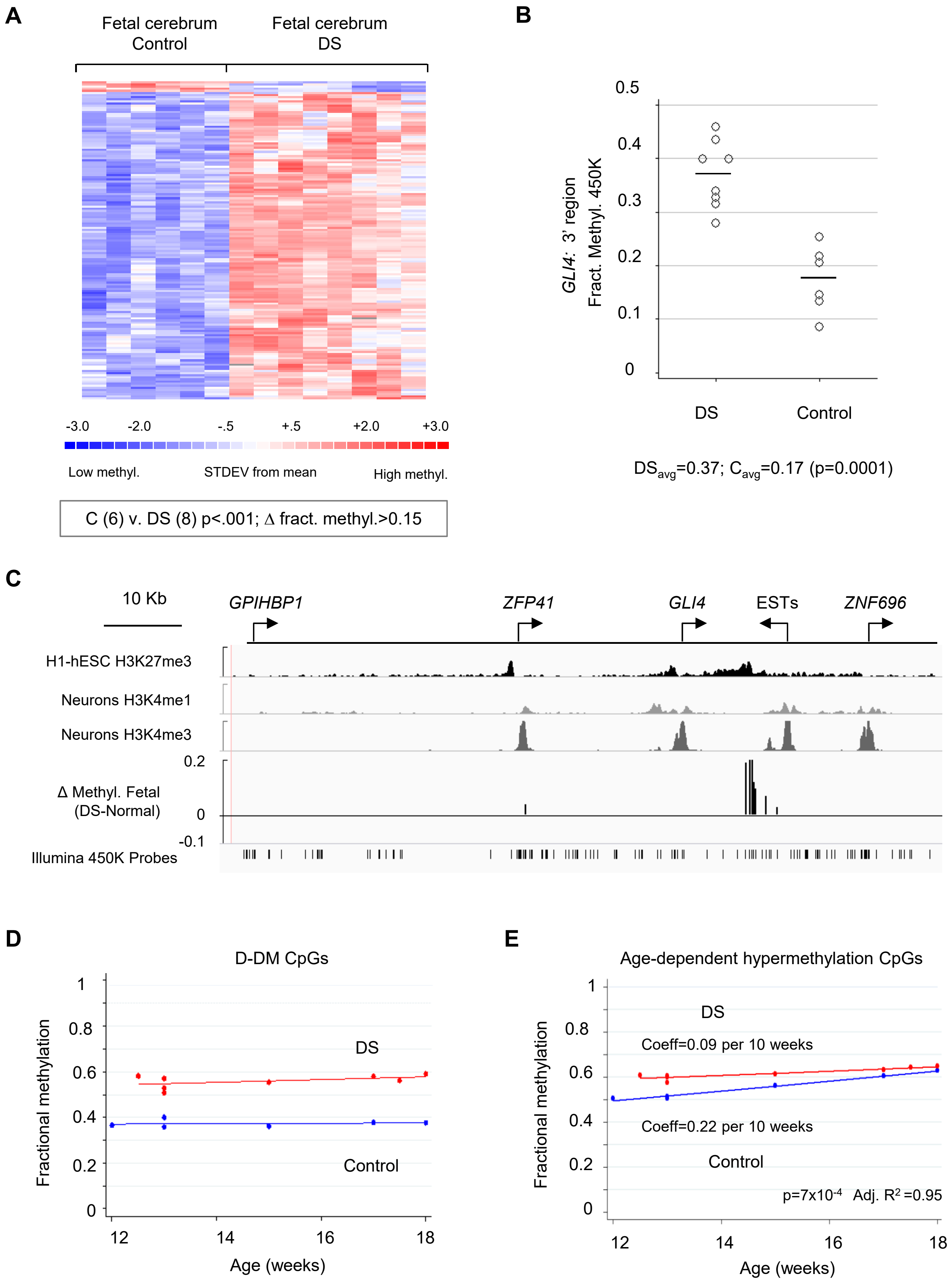

Suppl. Fig. S14

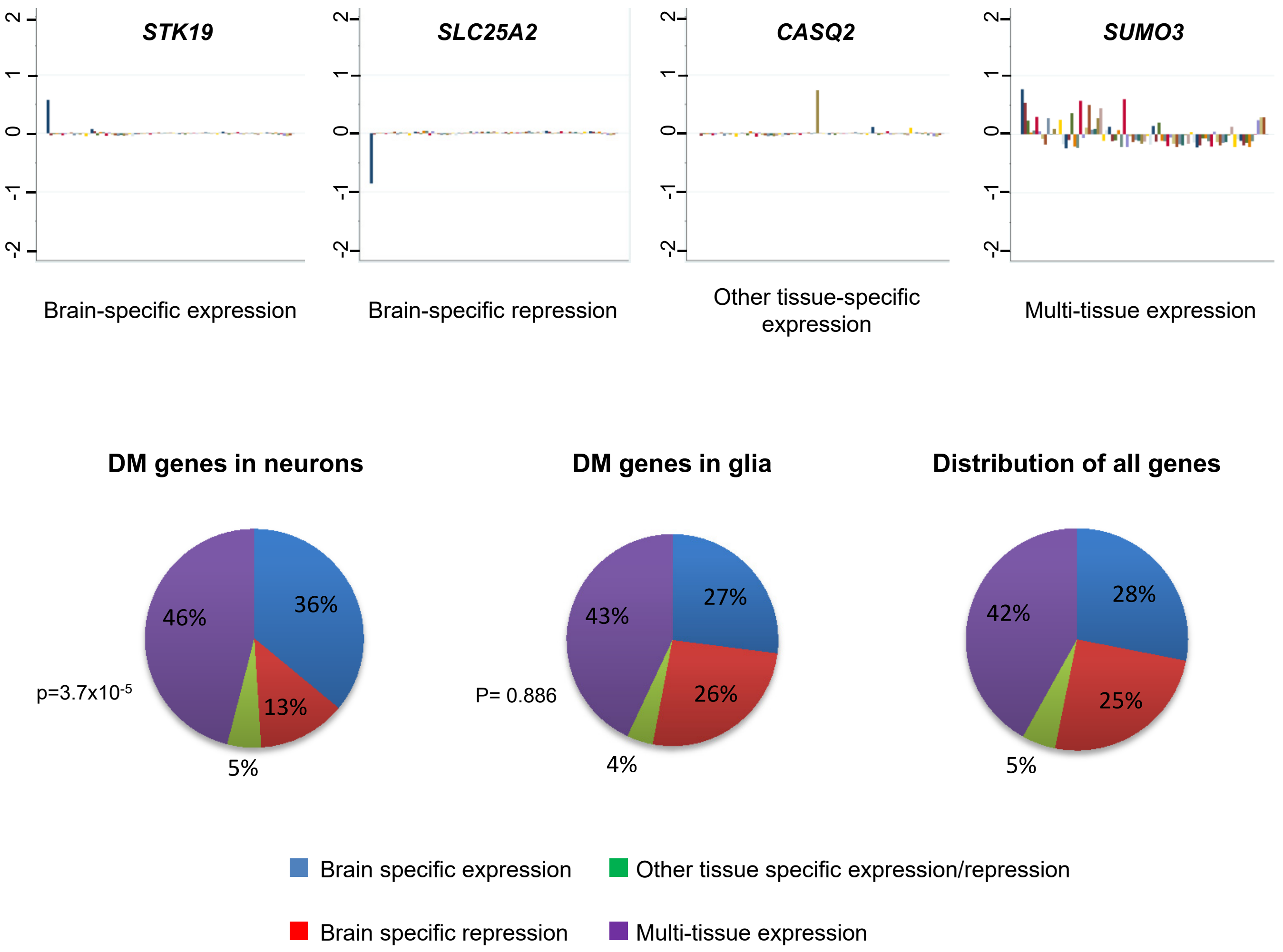

Suppl. Fig. S15

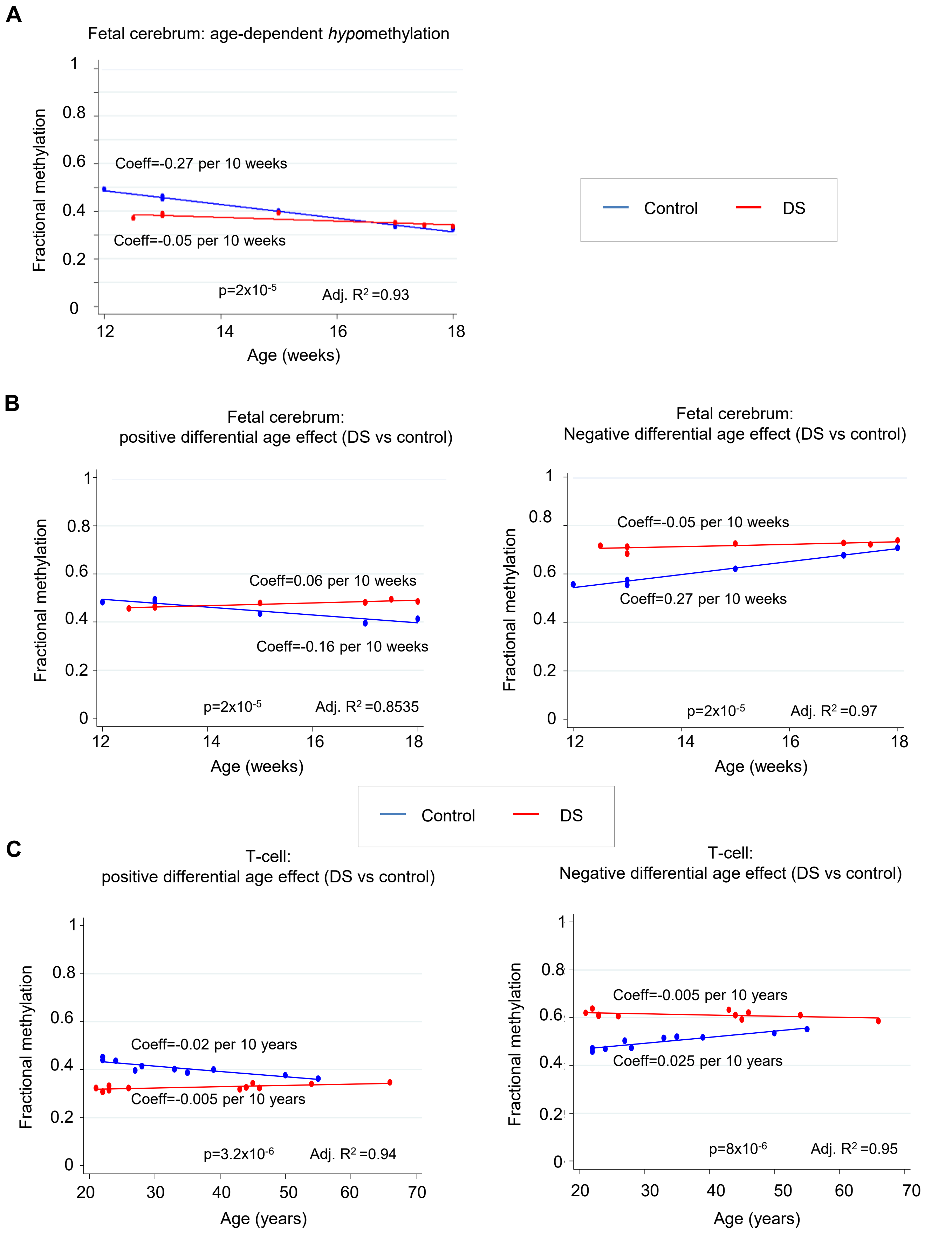

Suppl. Fig. S16

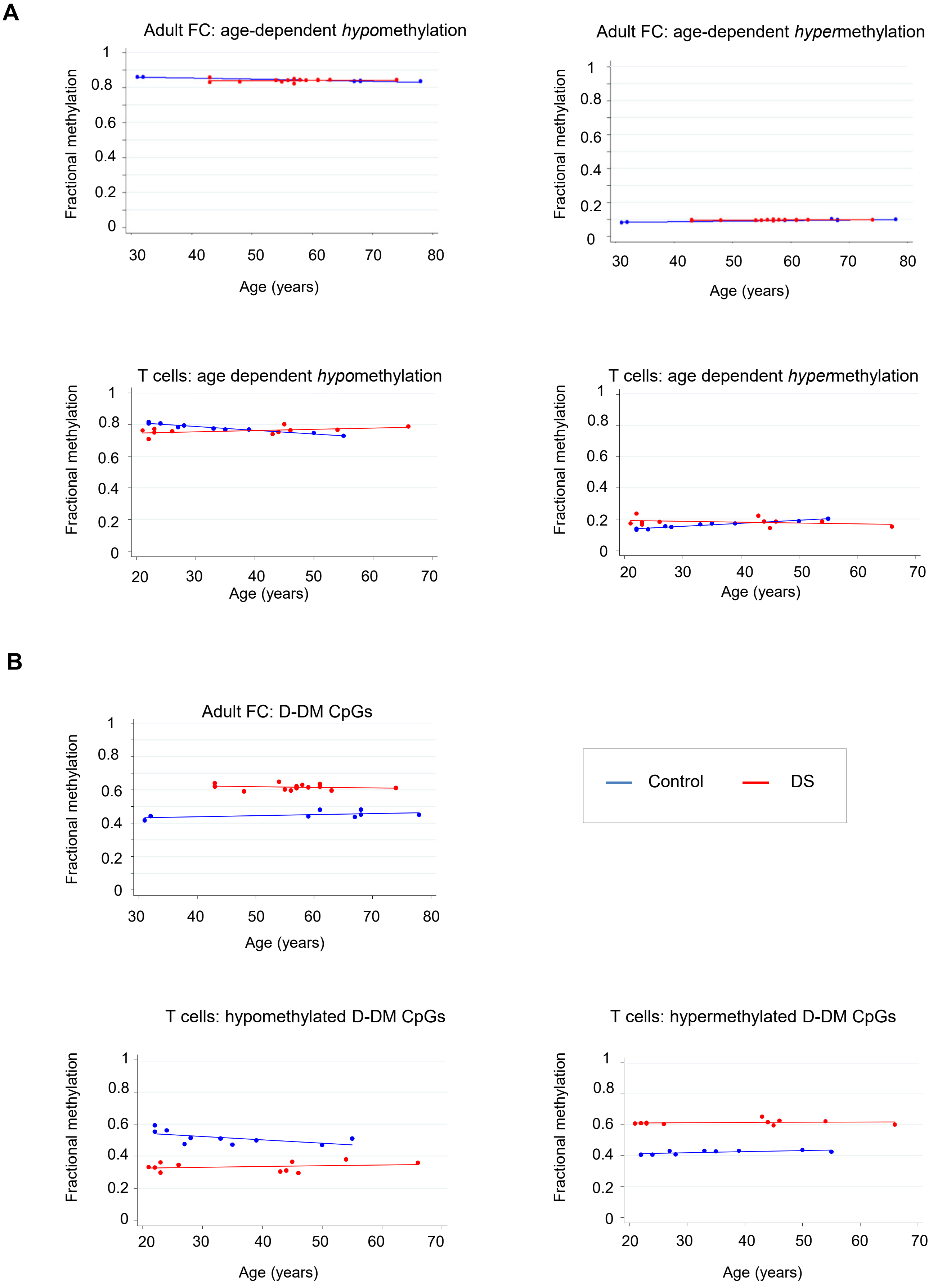

Suppl. Fig. S17

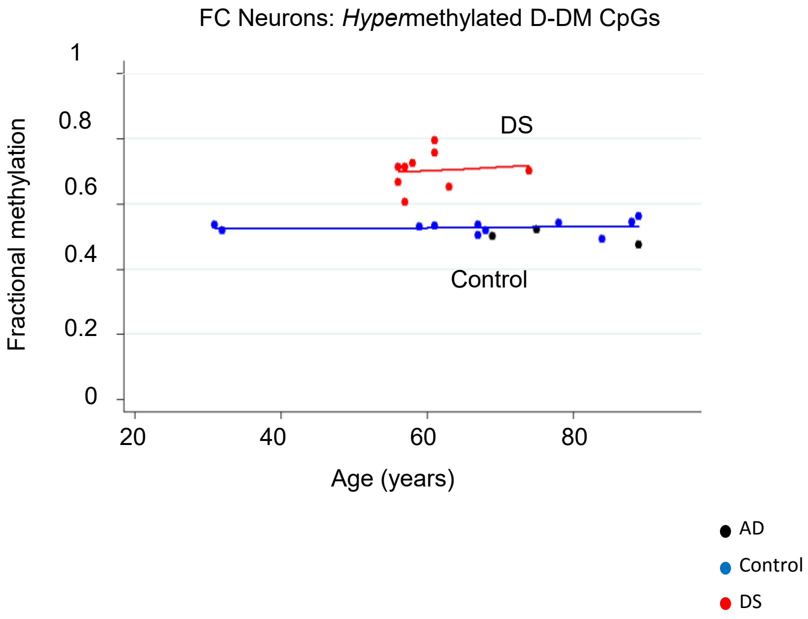

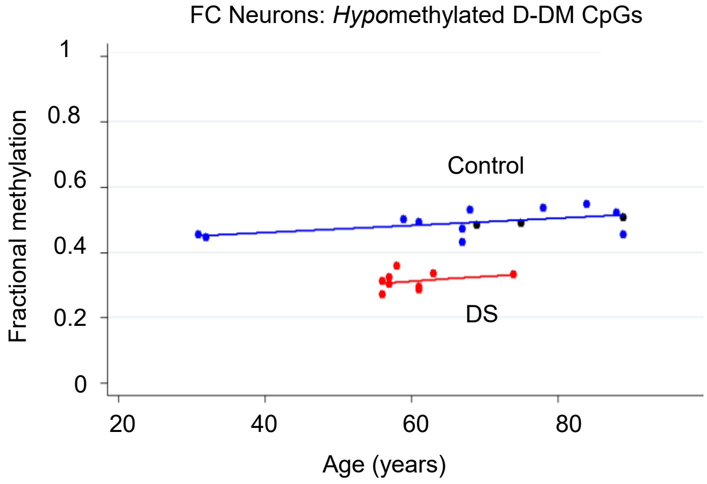

Suppl. Fig. S18

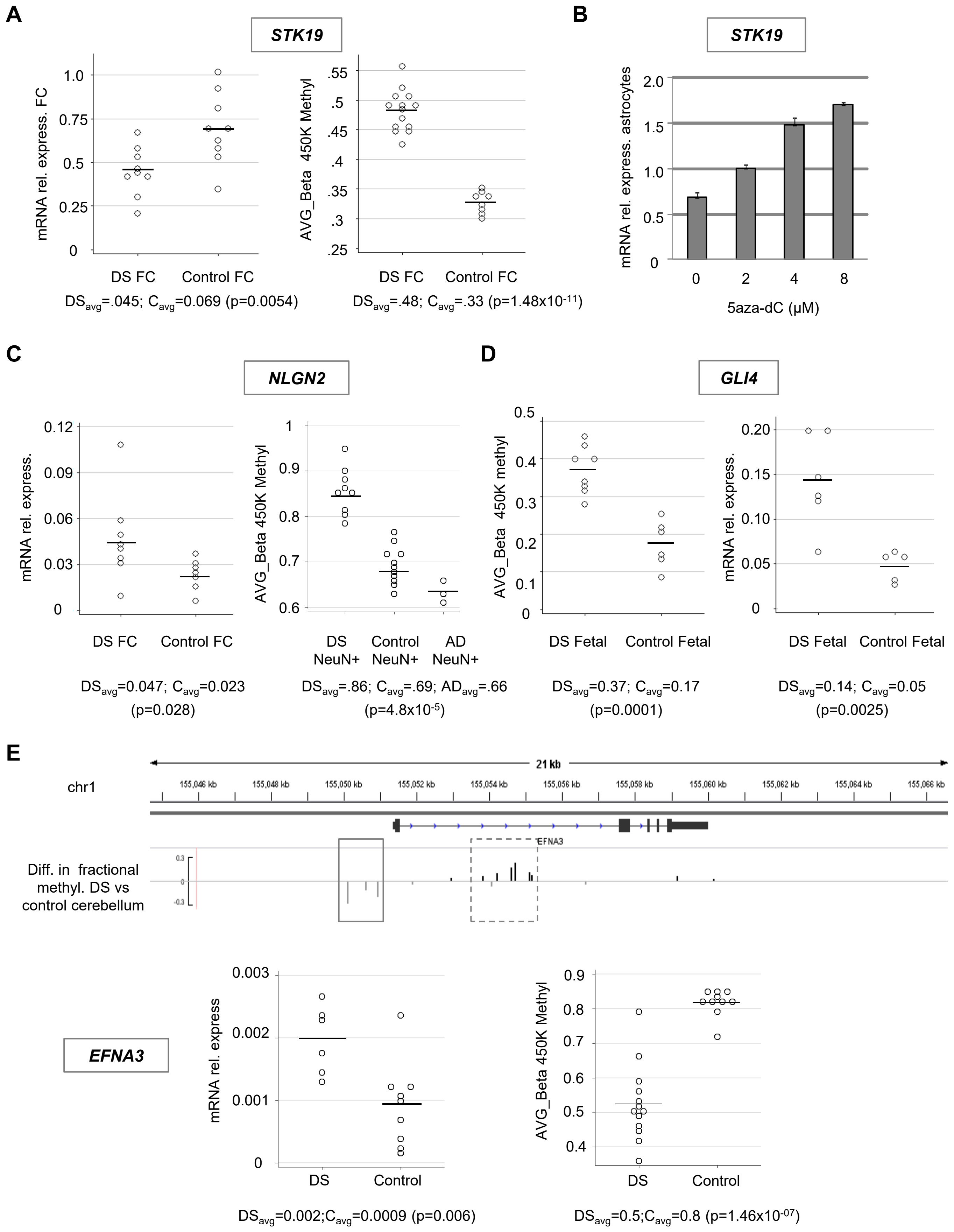

Suppl. Fig. S19

A

*Hypo*methylated genes in *glia vs neurons*
Top 10 GO terms

GO Terms	Fold Enrichment	FDR
modification by symbiont of host morphology or physiology	8.9	2.6E-02
phospholipid-translocating ATPase activity	6.3	2.8E-02
cyclic-nucleotide phosphodiesterase activity	4.5	4.9E-02
regulation of cell shape	3.3	7.4E-03
regulation of JNK cascade	3	3.6E-02
positive regulation of defense response	2.9	2.2E-02
postsynaptic density	2.9	2.4E-02
tight junction	2.8	3.0E-02
endocytosis	2.6	4.5E-06
phosphoinositide binding	2.5	2.4E-02

*Hypo*methylated genes in *neurons vs glia*
Top 10 GO terms

GO Terms	Fold Enrichment	FDR
regulation of ryanodine-sensitive calcium-release channel activity	14.9	3.1E-02
expression in frontal cortex	10.6	2.5E-06
calcium channel regulator activity	8.6	3.2E-02
regulation of ion transmembrane transporter activity	7.5	2.0E-02
neurotransmitter secretion	7.0	1.9E-03
phospholipid transporter activity	6.0	4.0E-02
postsynaptic density	5.9	2.1E-06
memory	5.3	2.9E-02
postsynaptic membrane	5.2	7.8E-10
Rho guanyl-nucleotide exchange factor activity	4.8	5.7E-04

B

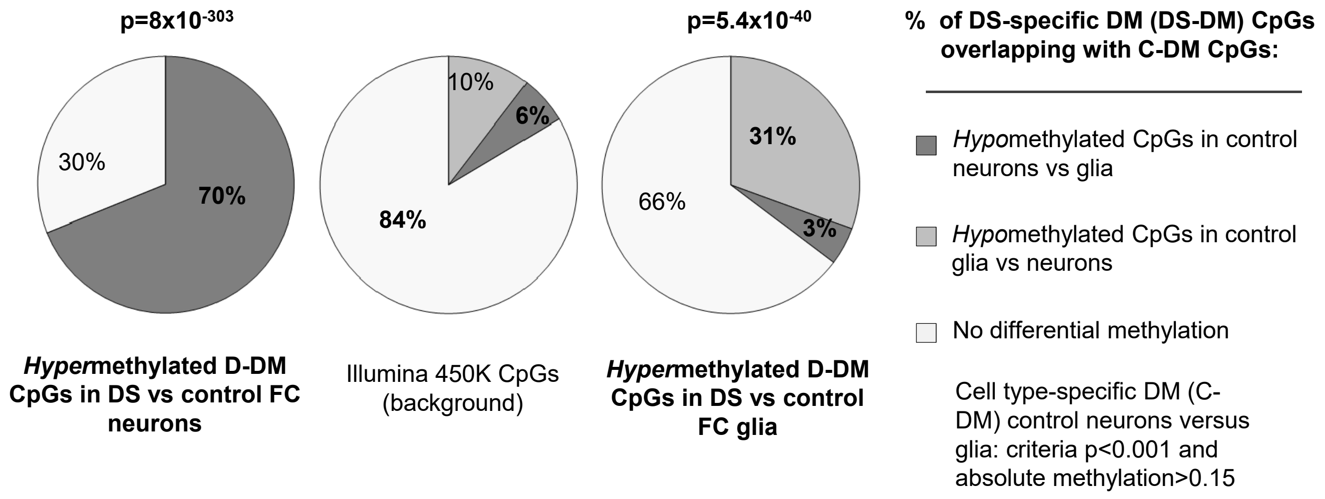

Suppl. Fig. S20

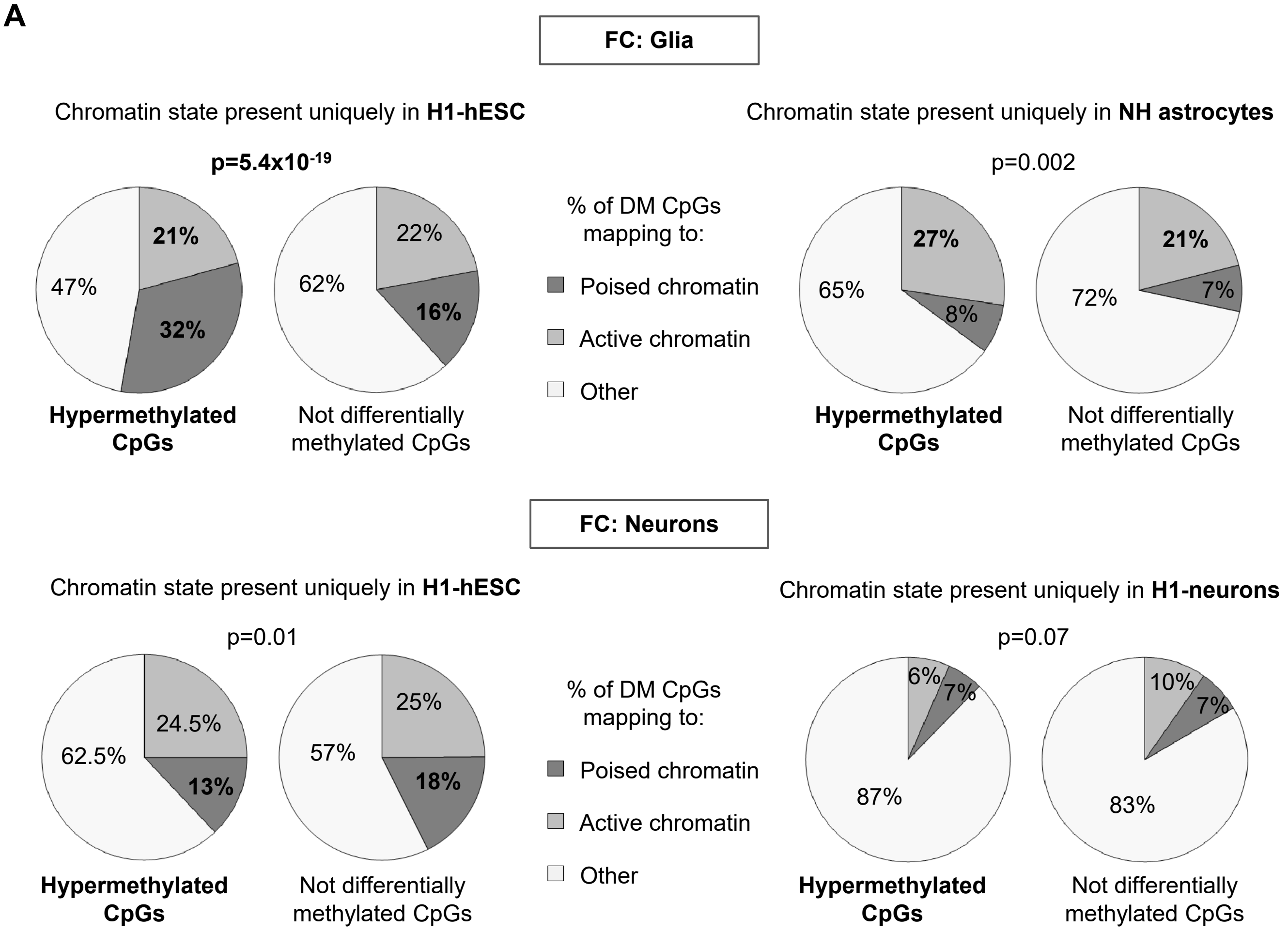

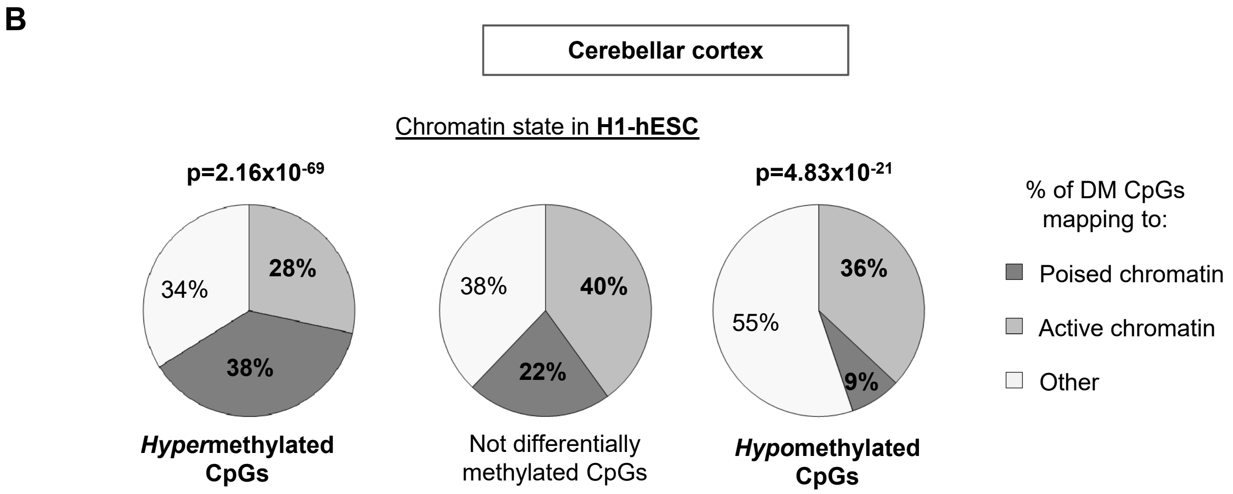

Suppl. Fig. S21

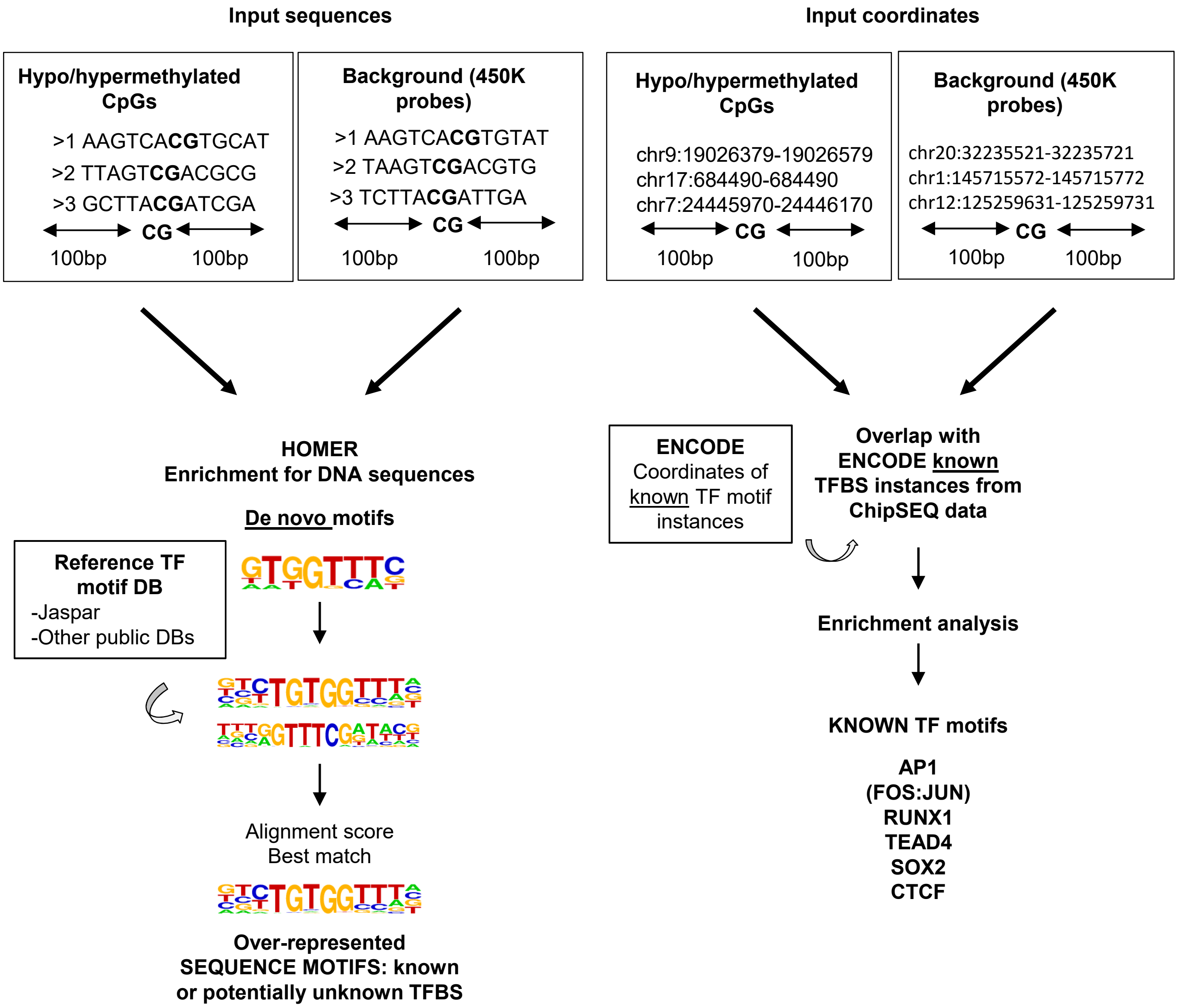

Suppl. Fig. S22

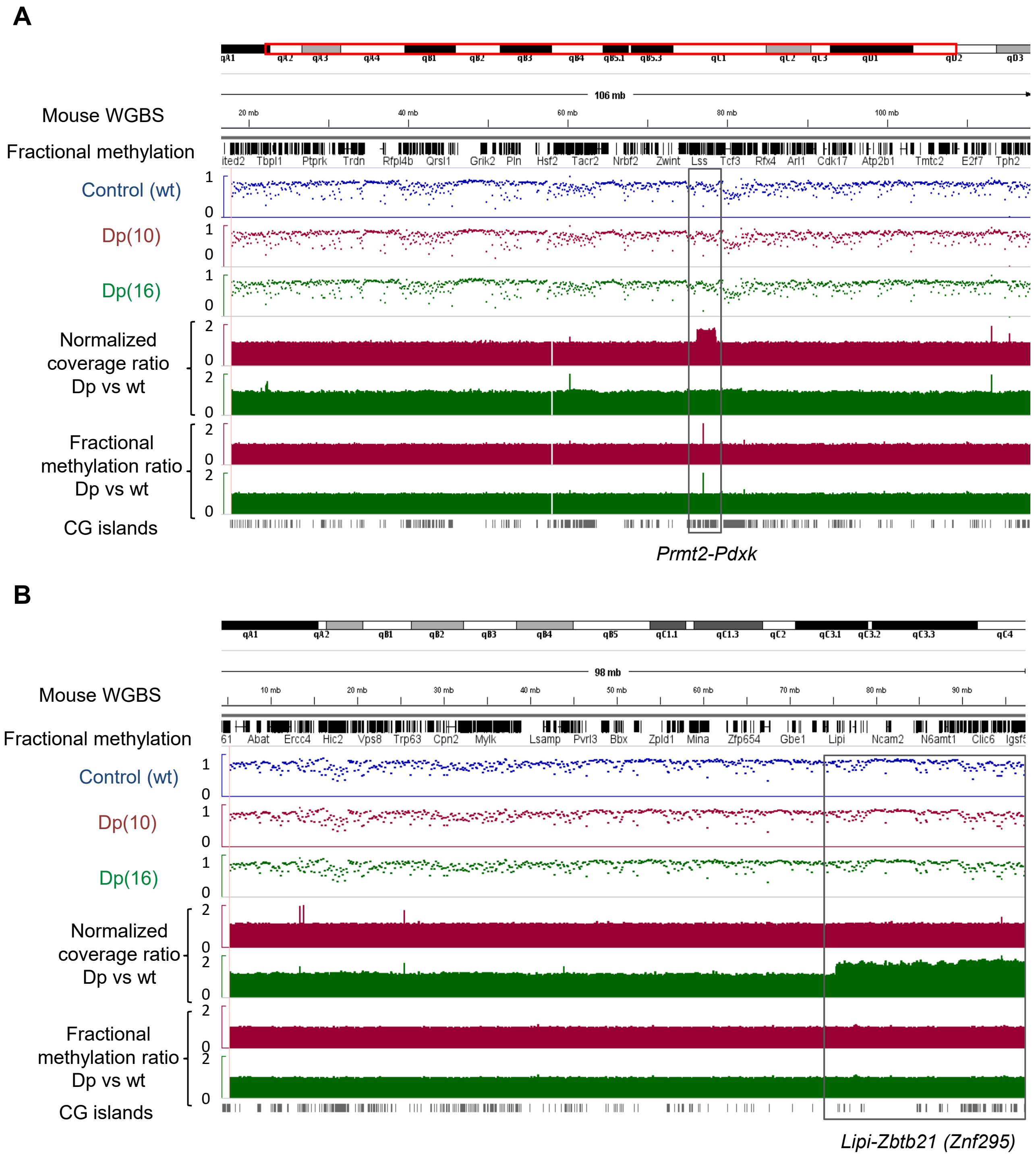

Suppl. Fig. S23

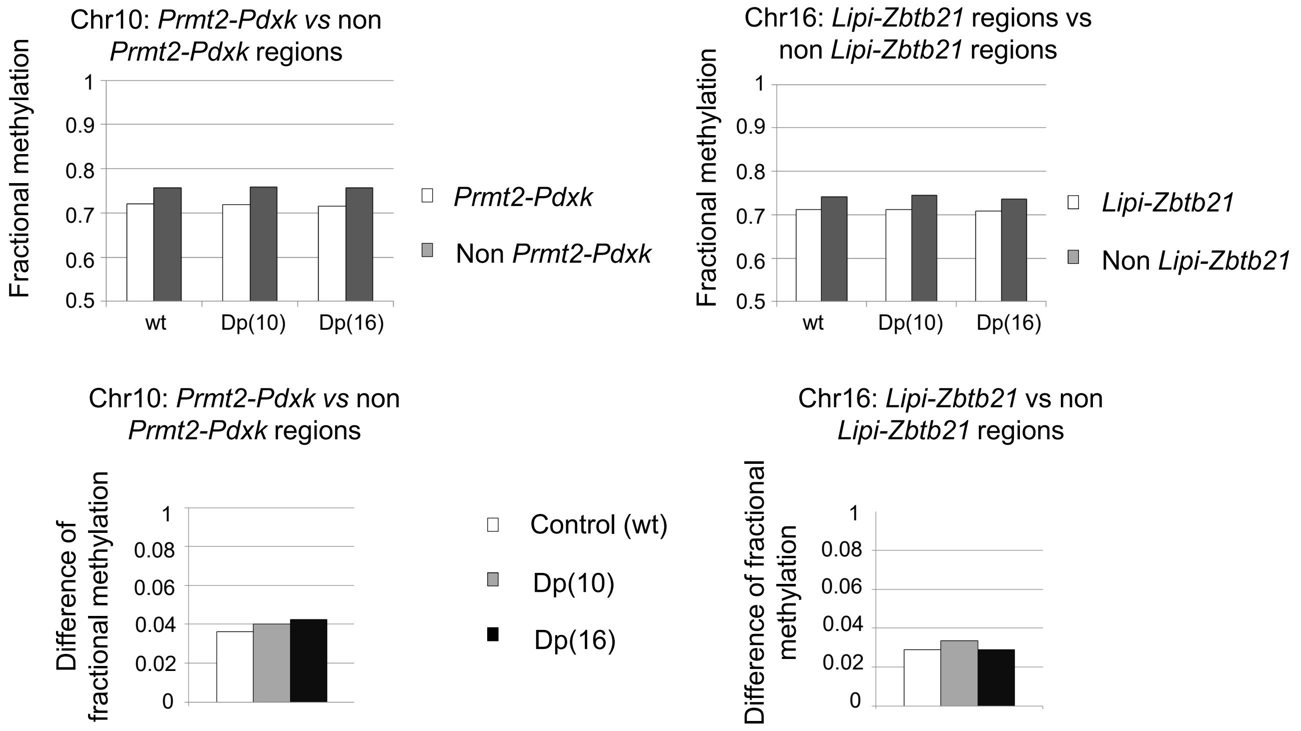

Suppl. Fig. S24

A

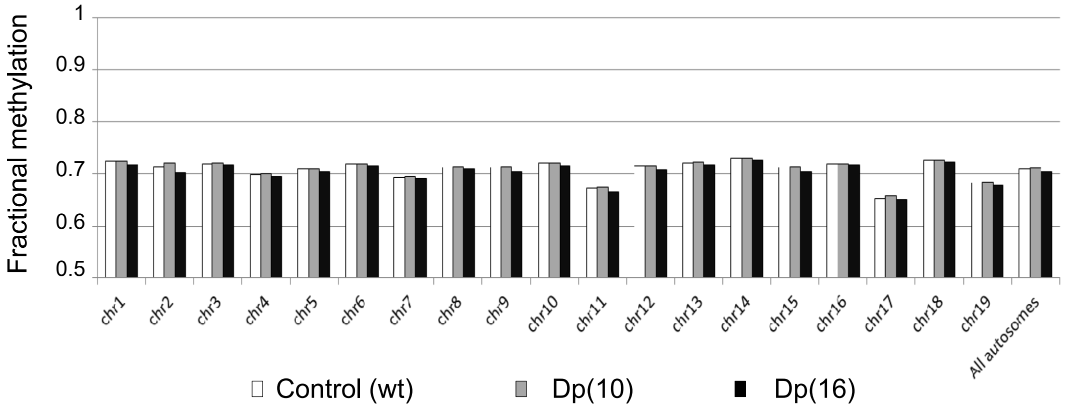

Average fractional methylation by chromosome in control, Dp(10) and Dp(16) mice (WGBS)

B

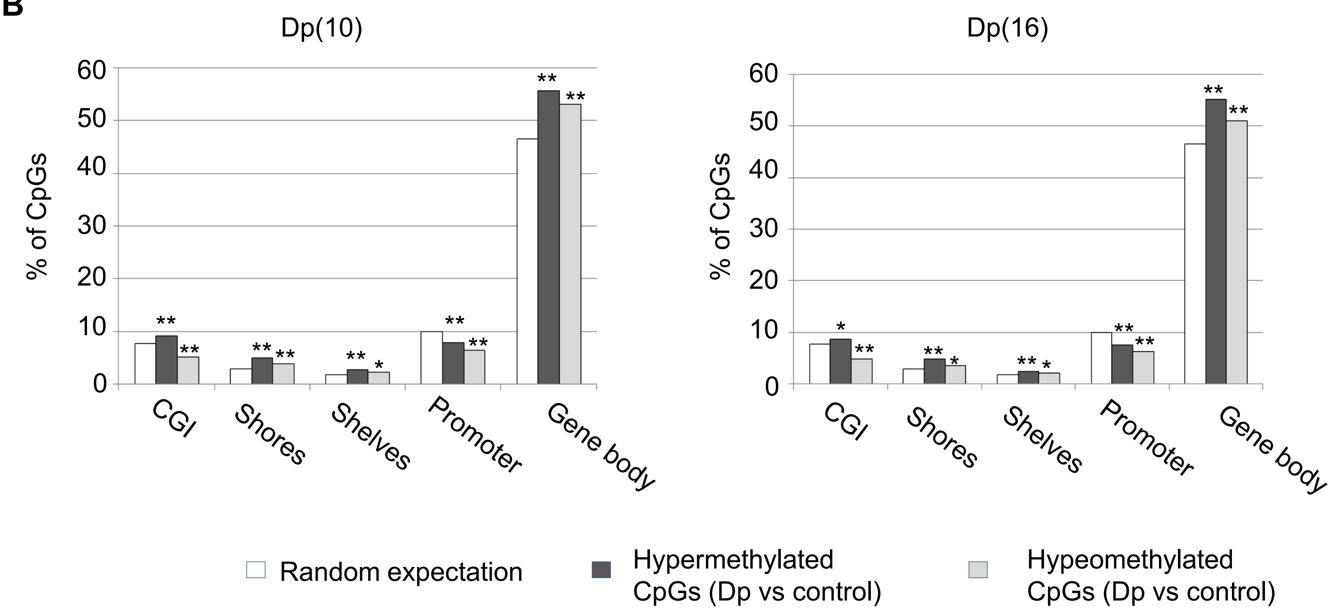

Suppl. Fig. S25

A

Enrichment for human *hyper*methylated DS-DM gene orthologues among Dp(10)1Yey and Dp(16)1Yey mouse DM genes

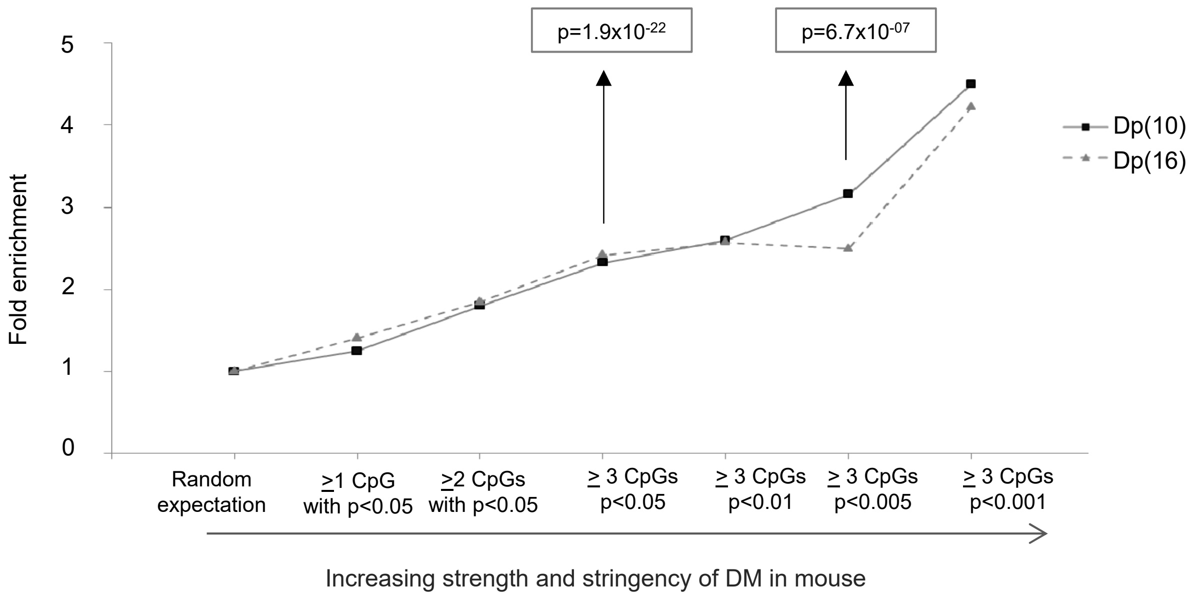

B

Inclusion of human *hyper*methylated human DS-DM genes among Dp(10) and Dp(16) mouse DM genes

Human DS-DM genes; p<0.005; Δ methyl.>+ 0.15

Human DS-DM genes; p<0.005; Δ methyl.> + 0.15

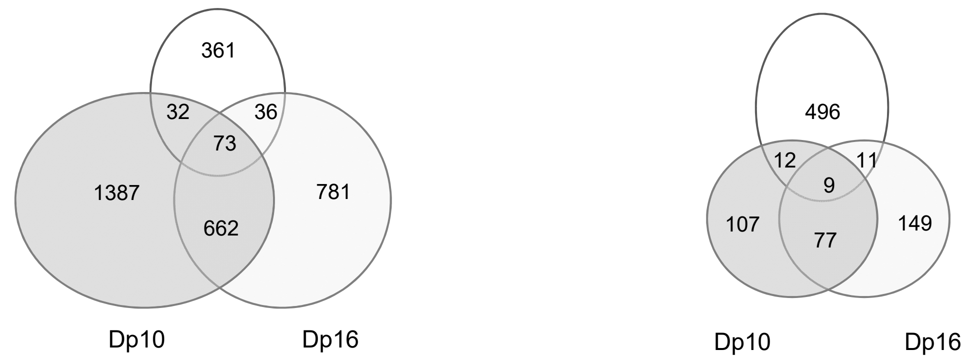

Mouse DM genes;
≥3 DM CpGs in a 1kb window (p-value<0.05); all with Δ methyl.> + 0.15

Mouse DM genes;
≥3 DM CpGs in a 1kb window (p-value<0.005); all with Δ methyl.> + 0.15

Suppl. Fig. S26

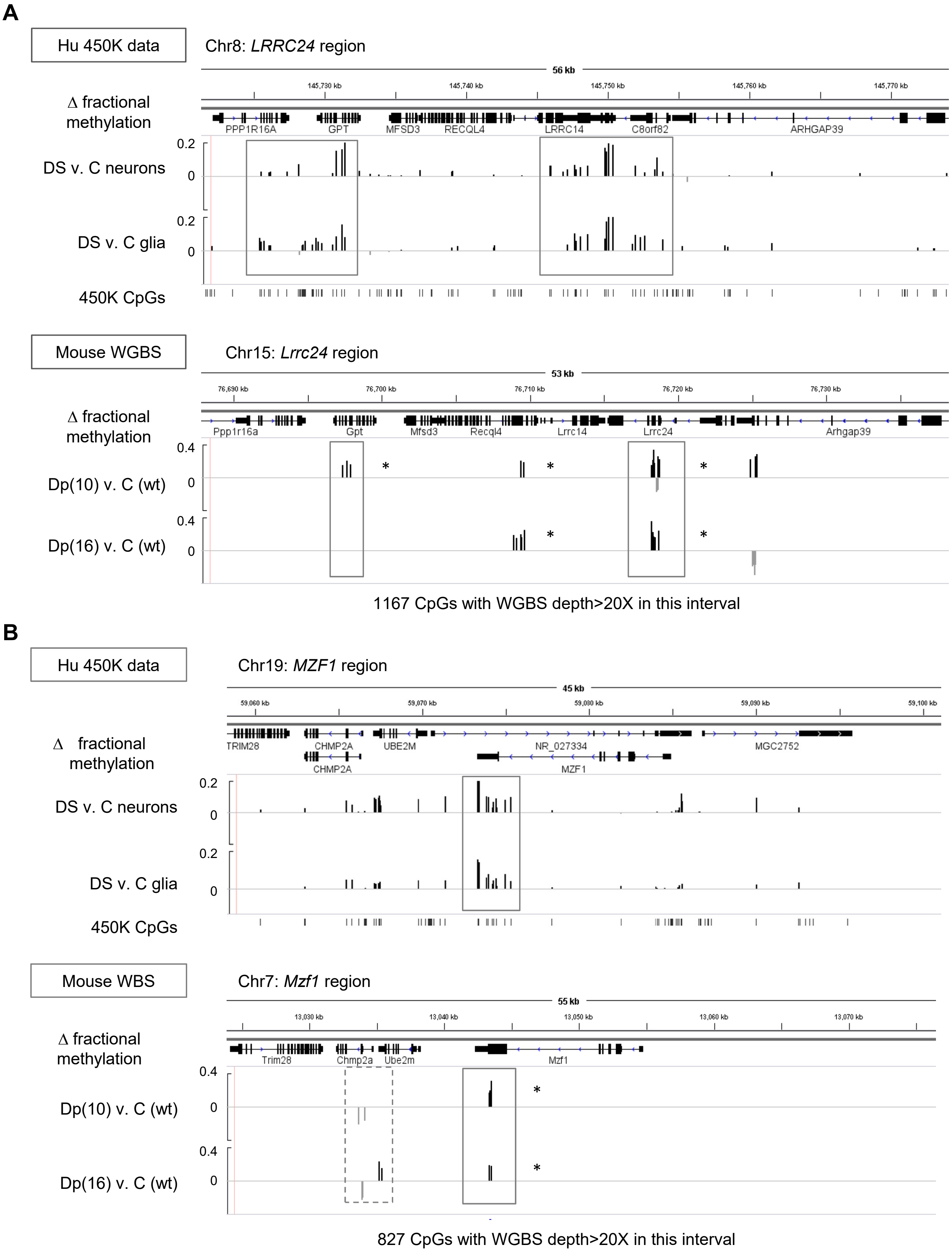

Suppl. Fig. S27

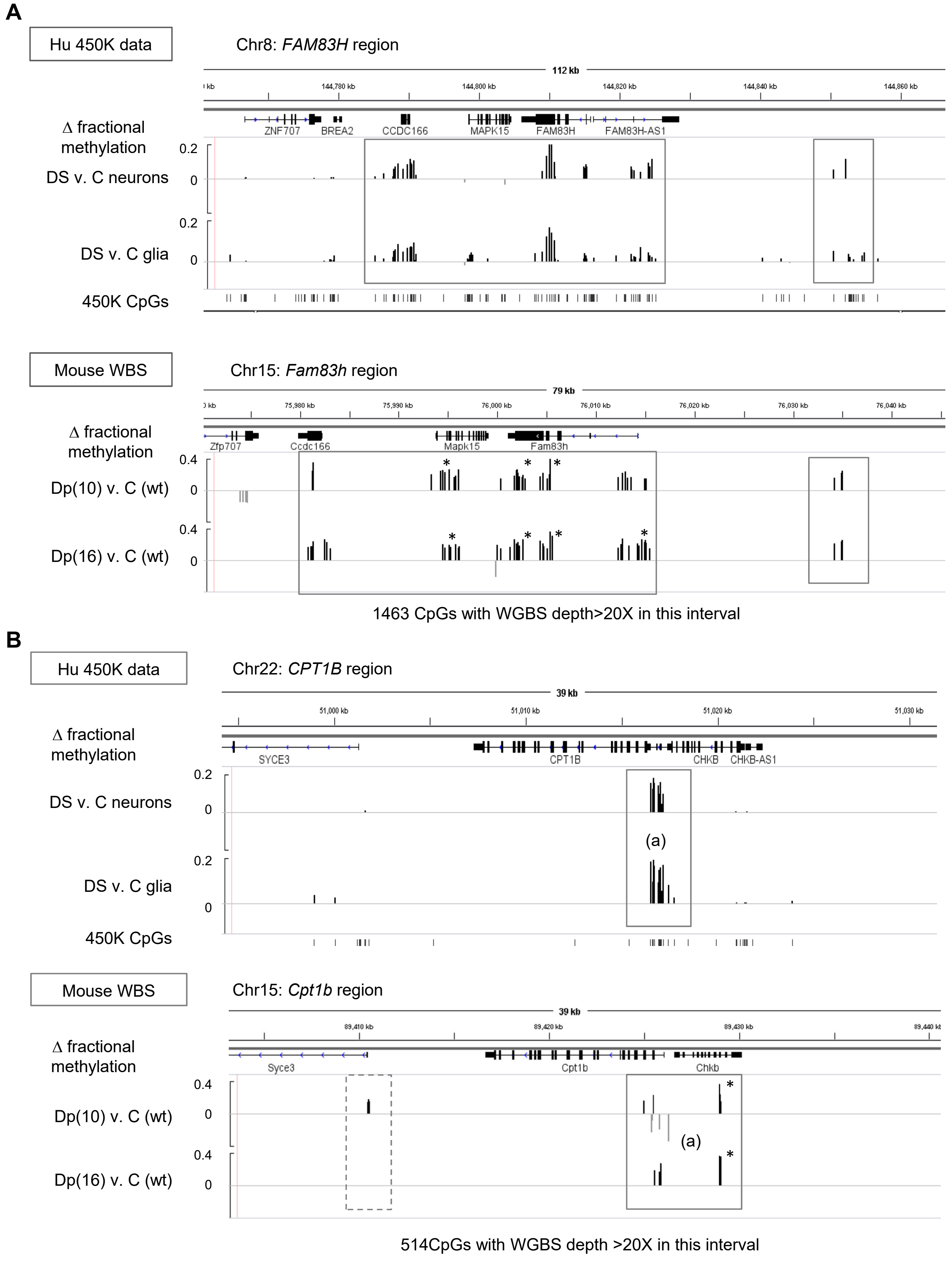

Suppl. Fig. S28

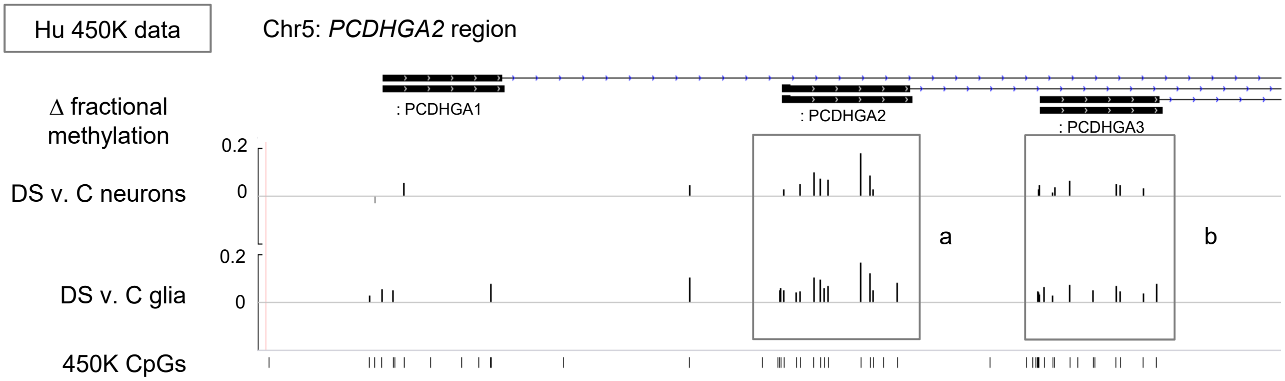

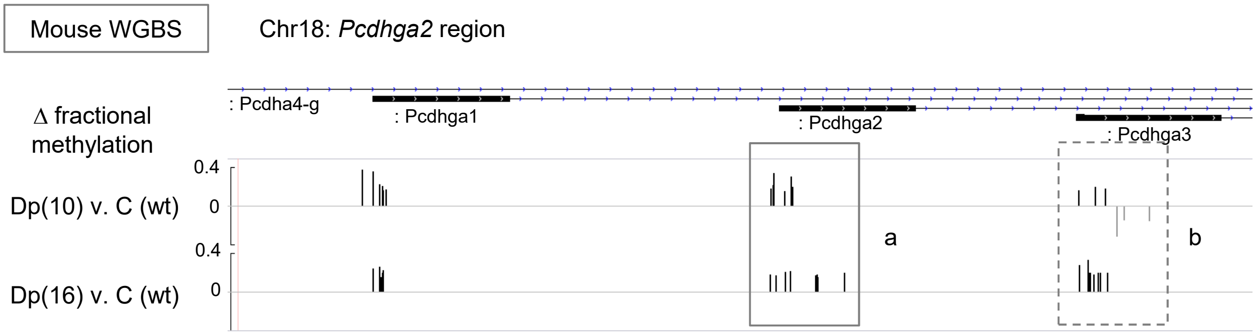

251 CpGs with WGBS depth >20X in this interval

Suppl. Fig. S29

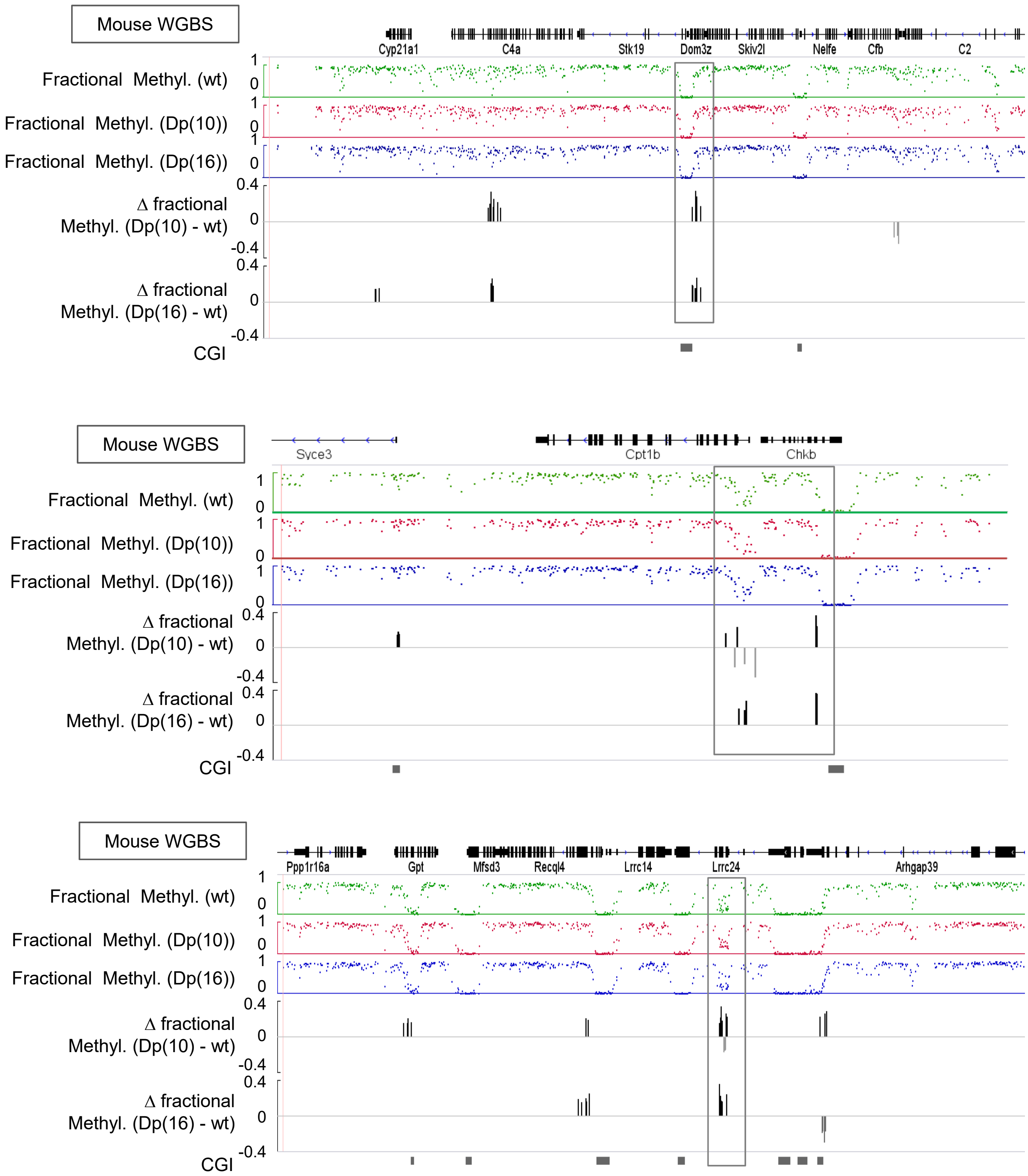

Suppl. Fig. S30

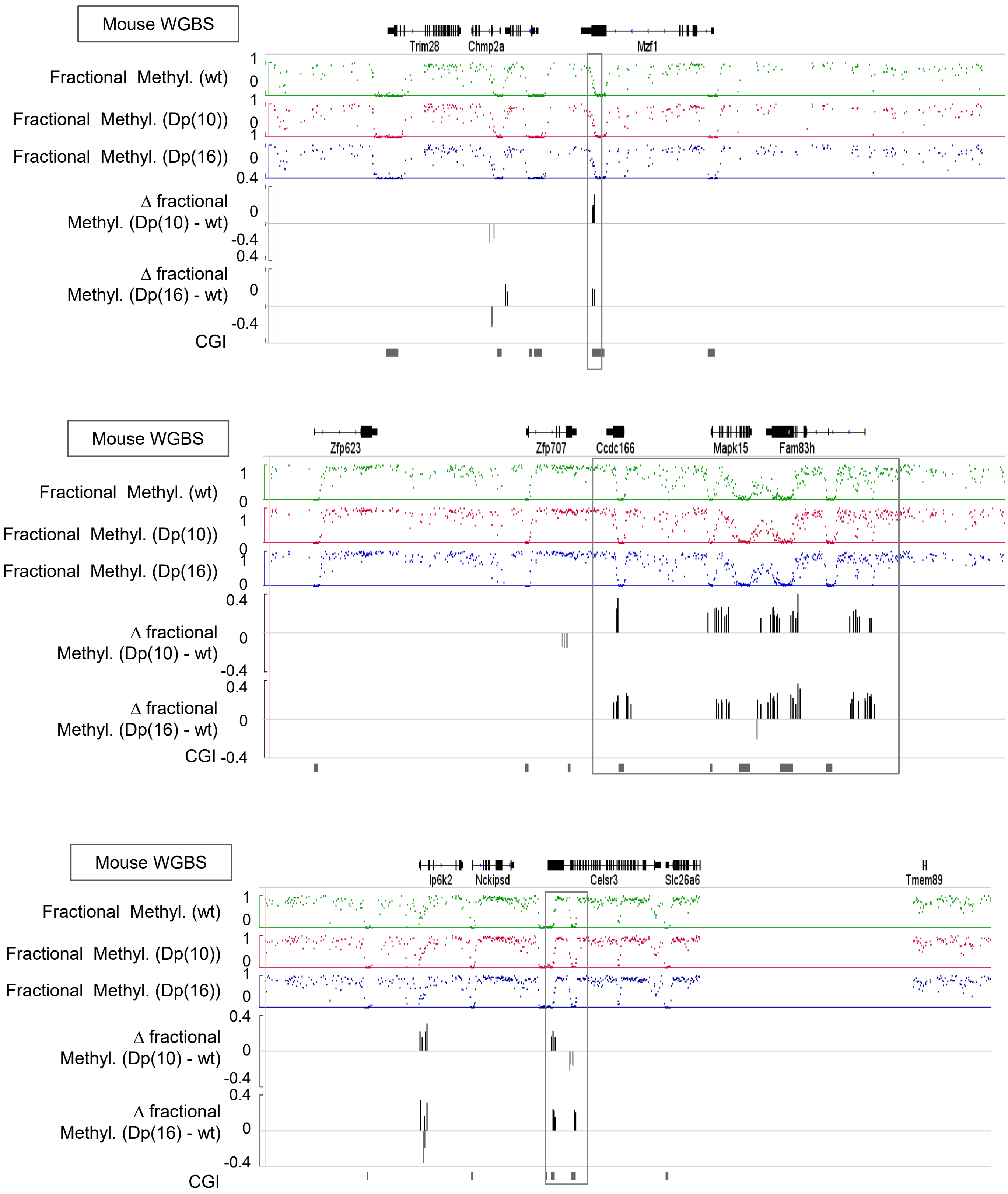

Suppl. Fig. S31

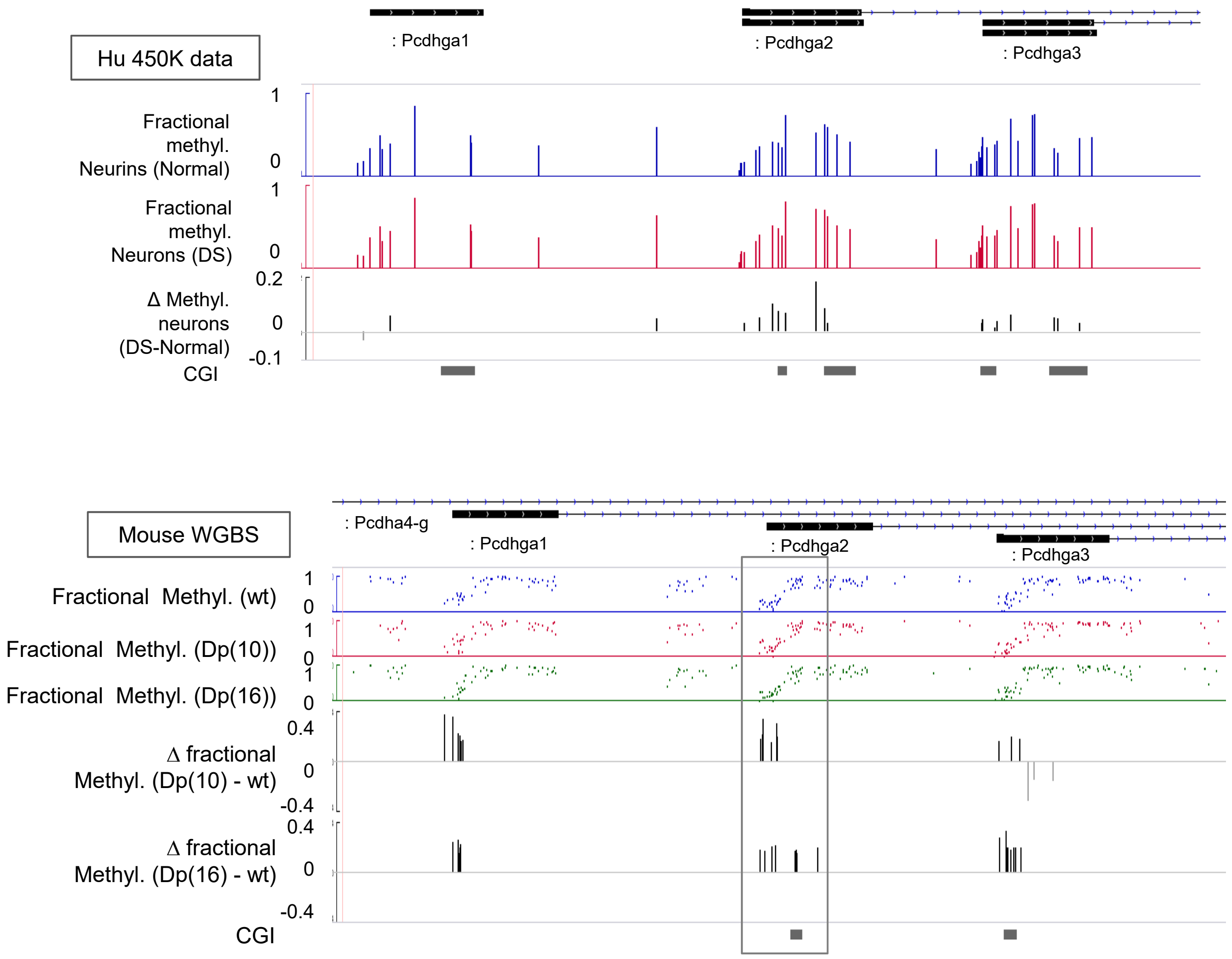

Supplement: Additional file 1: — Supplemental figures. (ZIP 1688 kb) [file 13059_2015_827_MOESM1_ESM.zip › DS Suppl Figures GB4.pdf]
