# Supplementary material for: Trans effects of chromosome aneuploidies on DNA methylation patterns in human Down syndrome and mouse models
Source: Genome Biol. 2015 Nov 25;16:263. doi: 10.1186/s13059-015-0827-6 (PMC4659173; doi:10.1186/s13059-015-0827-6)
Supplement: Additional file 1: — Supplemental figures. (ZIP 1688 kb) [file 13059_2015_827_MOESM1_ESM.zip › Fig .pdf]

Supplemental Figures

Figure S1 (A, B). Bioinformatics pipeline utilized in this study.

A, Procedures for 450K methylation data quality control and initial non-supervised analyses. **B,** Procedures for annotating the sets of DS-DM loci and enrichment analyses to identify pathways and test mechanistic hypotheses.

Figure S2. Confirmation of trisomy 21 in DS brains by DNA copy number analysis, separation of NeuN-positive neuronal and NeuN-negative glial cell nuclei, and analysis of cell type-specific DNA methylation.

A, Chromosome 21 and X chromosome copy number analysis for adult and fetal DS cases and age-matched normal brain samples, confirming complete 21 trisomy in the DS cases and concordance of X chromosome copy number with the sex of each study subject (F, Female; M, Male). The copy number analysis was carried out using intensity data from the 450K Infinium arrays, after normalization and model-based expression in dChip. These cases are representative; all DS cases showed complete trisomy 21. **B,** Cell nuclei from frontal cortex grey matter were separated by FACS: NeuN-positive neuronal nuclei and NeuN-negative non-neuronal nuclei (mostly glia) are identified by anti-NeuN-Alexa488 (green) and total nuclear DNA is counterstained using To-Pro3 (red). **C,** Cell type specificity of DNA methylation patterns highlighted by displaying the 450K methylation data as a correlation heat map. The pattern of methylation in whole FC is positively correlated, as expected, with the purified neuronal and glial cell fractions, but is not positively correlated with that in fetal cerebrum, adult cerebellum or T cells. **D,** Principle component analysis (PCA) of the 450K methylation data showing clustering of the samples according to cell type and stage. In this non-supervised analysis in a given cell type and stage there is no obvious separation by DS vs. control status, with the exception of the cerebellar cortex, in which the DS samples are clearly separated from the controls, even by this non-supervised approach.

Figure S3. Relationship of T-test p-values to False Discovery Rates (FDR) in each tissue and cell type.

A, The bars represent the number of DS-DM CpGs in each dataset that passed decreasing t-test p-value cutoffs. For each uncorrected p-value cutoff, the corresponding FDR is indicated. The FDR depends on the number of significant CpGs (based on the uncorrected p-value). While a FDR<0.05 corresponds to an uncorrected p-value<$5x10^{-3}$ in glia and cerebellum, in fetal brain it corresponds to a p-value<$5x10^{-6}$. In the fetal brains, higher inter-individual variability is seen, presumably reflecting both rapid methylation

changes during each week of fetal brain development, and additional inherent variability in the DS cases. Thus, for some purposes, a significance cutoff controlling FDR at the 5% level is too conservative for analyzing the fetal brain data. **B**, Graph showing the study power to detect a difference in fractional methylation >0.15 with an uncorrected p-value=0.005, for each comparison set. The power is calculated for the 50th to 99th percentile of methylation. In this graph the STDEV corresponds to the STDEV value for which 50% of the loci have a STDEV smaller than this value. In neurons and glia, the study power is >80% for ~95% of the loci (up to the 95th percentile of the methylation STDEV). For the fetal brain data, ~20% of Illumina 450K BeadChip array was assessed with a power<80%, as compared to 5% for the glia and neuron data.

Figure S4. Bis-seq confirming DS-DM in the *PDE11A*, *HOXA2*, *CPT1B* and *LRRC14* genes and showing that it affects multiple contiguous CpGs around the index CpGs queried by the 450K arrays.

Bisulfite sequencing confirms and extends the array data for *PDE11A* (**A**), *HOXA2* (**B**), *CPT1B* (**C**) and *LRRC14* (**D**). Maps of the differentially methylated loci are shown at the bottom of each panel. The bis-seq amplicons are represented by grey bars and the CGIs by white bars.

Figure S5. Validation of DS-DM in the promoter region of the *C21of56* gene in DS FC, neurons and glia.

A, Bis-seq validation and extension of the 450K methylation data for DM at the 5' end of the gene *C21orf56* in brain, neurons and glia from DS and control adult FC. The map of the *C21orf56* locus shows the alternative mRNA transcripts, bis-seq amplicon, 5' and 3' CGIs. The bis-seq amplicon (grey bar) is located at the 5'end of the gene, upstream two CGIs (white bars). The differential methylation pattern at the 5' end of *C21orf56* in both neurons and glia parallels the methylation patterns observed in the whole FC. **B,** AVG_Beta values from the BeadChip data for the highest scoring DM probe (cg07747299) showing an increase in average fractional methylation in DS compared to control whole FC, neurons and glia.

Figure S6. Bis-seq validations of weak but definite DS-DM in the *ESR1* and *RUNX1* genes in brain tissues.

A, Map of the *ESR1* gene and bisulfite validation of the 450K BeadChip data showing a gain of methylation at the 5' end of the gene in DS versus control glia and whole FC grey matter. The bis-seq amplicon (asterisk) is slightly downstream of a CGI (white bar) that overlaps the alternative 1st exon of the gene. **B,** AVG_Beta values from the BeadChip data averaged for two probes in *ESR1* (cg04063345; cg15626350) in glia, showing a 0.17 increase in fractional methylation in DS compared to control, with no overlap between cases and controls. **C,** Map of the *RUNX1* gene and bisulfite validation of the 450K

BeadChip data showing a gain of methylation at the 5’ end of the gene in DS versus control samples in glia and whole FC grey matter. The bis-seq amplicon (asterisk) spans a CGI (white bar) in 1st exon of the gene. **D,** AVG_Beta values from the BeadChip data averaged for two probes in *RUNX1* (cg00994804; cg06758350) in whole FC grey matter, showing an increase in average fractional methylation in DS compared to control (Y-axis is expanded to show that the difference is small in magnitude but with little overlap between DS cases and controls). The horizontal lines show the mean methylation values. Histone ChIP-seq data were downloaded from histone tracks (ENCODE for NH-A and H1-ESC, and Zhu et al, Cell 2013 for histone marks in H9 derived neurons; GSE17312 and GSM733758, repectively).

Figure S7. Bis-seq validations of strong DS-DM in the *ZDHHC14*, *LRRN3*, and *RUNX1* genes in DS vs. control T-lymphocytes.

A, Intragenic CG-rich region in the *ZDHHC14* gene. **B,** Promoter/exon 1 region of the *LRRN3* gene. **C,** Promoter/exon 1 region of the *RUNX1* gene. Maps of each gene are shown below the bis-seq data, with the bis-seq amplicons indicated by the grey rectangles and CGIs indicated by the white rectangles. **D,** Scatter plots of the AVG_beta (fractional methylation) values for these three loci. Fractional methylation was averaged across all significant CpGs within the gene for *ZDHHC14* and *RUNX1* (absolute difference >0.15 and p-value<0.001).

Figure S8. Methylation values (450K) for all queried CpGs in the *NLGN2*, *MZF1*, and *STK19* DS-DM regions

Fractional methylation from human 450K data in control and DS neurons for *NLGN2* region and frontal cortex for *MZF1* and *STK19* regions, as well as difference in fractional methylation in DS compared to control ($p<0.05$) in are shown below the map of each region. The hypermethylated region in *NLGN2* (rectangle) is coincident with a high methylated CGI, while the hypermethylated region in *MZF1* overlaps a low methylated CGI. Hypermethylation in *STK19* is coincident with a high methylated CGI shore.

Figure S9. Methylation values (450K) for all queried CpGs in the *AMH*, *ZMAT3*, and *EFNA3* DS-DM regions

Fractional methylation from human 450K data in control and DS cerebellum shows that hypermethylation in *AMH* region occurs in an intermediate methylated CGI. The hypomethylated CpG in *ZMAT3* is located in a low methylated CGI. The two DS-DM regions in *EFNA3* are flanking a low methylated CGI.

Figure S10. Methylation values (450K) for all queried CpGs in the *ESR1*, *GLI4*, and *RUNX1* DS-DM regions

The hypermethylation in *ESR1* is coincident with a high methylated CGI in frontal cortex while hypermethylation in the *GLI4* region overlaps a low methylated CGI in fetal brain. Hypermethylation in *RUNX1* is coincident with a CGI that is low methylated in frontal cortex and intermediate methylated in T cells.

Figure S11. Modified bis-seq showing the relative contributions of 5mC and 5hmC to DS-DM in the *ZMAT3* and *GLI2* genes in DS vs. control adult cerebellar cortex.

A, Map of the *ZMAT* gene and results of modified bis-seq in DS and control cerebellar cortex. The contribution of 5hmC to the DS-DM is inferred from the difference between the percent methylation at each CpG in the 5mC+5hmC reactions compared to the 5mC-only reactions (see Figure 5 in main text). For this gene, most of the DS-DM is due to a reduction in 5hmC in DS. The amplicon is indicated by the grey bar. **B,** Map of the *GLI2* gene and results of modified bis-seq in DS and control cerebellar cortex. For this gene, the DS-DM is due partly to a gain of 5mC and partly to a gain of 5hmC in DS (see also **Suppl. Fig. S8**). The amplicon is indicated by the grey bar.

Figure S12. The modified bases 5mC and 5hmC contribute additively to DM in DS vs. control FC and DS vs. control cerebellar cortex.

A, Results of modified bis-seq for separately scoring 5mC and 5hmC (see Methods) for the *STK19* promoter region in DS compared to control adult FC. The bis-seq amplicon (grey bar) is immediately upstream of a CGI (white bar). The bar graph indicates the percent methylation of CpGs in the 5' third, middle and 3' third of the amplicon, separately for 5hmC (grey bars) and 5mC (black bars). In the 5' third of the amplicon the DM reflects a gain of 5mC in DS, while in the middle and 3' thirds the DM reflects gains of both 5mC and 5hmC in DS. **B,** Results of modified bis-seq for the *MZF1* 3' region in DS compared to control adult FC. The bis-seq amplicon (grey bar) overlaps the last exon of MZF1 and is located between a pair of CGIs (white bars). In the 5' third of the amplicon the DM reflects mostly a gain of 5mC in DS; in the middle of the amplicon it is a gain of 5hmC but not 5mC, and in the 3' third of the amplicon it reflects gains of both 5mC and 5hmC in DS. **C,** Results of modified bis-seq for the *ZMAT3* promoter/upstream enhancer region in DS compared to control adult cerebellar cortex. The bis-seq amplicon (grey bar) overlaps a regulatory region upstream of the promoter CGI (white bars). As shown in the map, it includes a CpG-containing binding site for the GABPA TF, which is known to have methylation-sensitive binding. In the 5' third of the amplicon the DM reflects loss of 5hmC in DS while in

the middle and 3' thirds of the amplicon it reflects losses of both 5hmC and 5mC. **D,** Results of modified bis-seq for a putative regulatory region near exon 2 of the *GLI2* gene in DS compared to control adult cerebellar cortex. The bis-seq amplicon (grey bar) overlaps a CG-rich sequence that does not meet criteria for a CGI. In the 5' third and 3' third of the amplicon the DM reflects gain of both 5mC and 5hmC in DS, while in the middle third there is a gain only of 5hmC. **E,** Methylation heatmap from the BS/OXBS protocol applied to cerebellar cortex DNAs, followed by analysis on 450K BeadChips. This protocol, which we carried out comparing DNA from 3 DS and 3 control cerebellar cortex samples, gave fewer reliable calls in the BeadChip data compared to the fully optimized standard Illumina protocol, but 380K CpGs still passed stringent quality control (p-value for detection$\leq$.005). Probes represented are the DS vs. control cerebellum DS-DM CpGs from the original larger series (FDR<.05). We further required that the probes pass QC pval<.005 in the BS/OXBS 450K data, and that in this smaller set of 3 vs. 3 cerebellar cortex samples the probes pass p<.05 uncorrected, for the BS AVG_Beta values (5mC+5hmC), in DS vs. control. The list of DS-DM CpGs passing these criteria is in **Supplemental Table S8a**. Both 5mC and 5hmC contribute to the net DM, and the direction of the difference in fractional methylation in DS vs. controls is the same for 5mC and 5hmC for a large majority (>90 percent) of the DS-DM CpGs. Clades "a" and "b" contain CpGs for which the difference in 5hmC makes a strong contribution to the DS-DM; for all other CpGs in this set the strongest contribution to the DS-DM is from differences in 5mC. The lists of DS-DM CpGs analyzed by applying T-tests and absolute change criteria directly to the BS/OXBS data from this 3 vs. 3 case-control experiment separately for 5mC (BS-OXBS) and 5hmC (OXBS), are in **Supplemental Table S8b, c**.

Figure S13. Gene specific gains of methylation in DS fetal brains.

A, Supervised hierarchical clustering of the 450K methylation BeadChip data for probes that passed ANOVA at p$\leq$0.001 and absolute difference in fractional methylation >.15 in DS versus control mid-gestation fetal whole cerebrum. Cases and controls are on the x-axis and differentially methylated loci are on the y-axis, with relative *hyper*methylation and *hypo*methylation indicated with red and blue respectively. **B,** Fractional methylation values for a DS-DM CpG in a CGI located at the 3' end of the *GLI4* gene. **C,** Map of the *GLI4* gene and flanking genes with ENCODE tracks for histone modifications aligned to the 450K methylation data (p-value < 0.005), showing that the CpGs with DM in DS versus control fetal cerebrum are tightly clustered in a region overlapping a CGI at the 3' end of the *GLI4* gene, which is marked by H3K27me3 in human ES cells and is adjacent to an intergenic peak of activating histone marks in neurons. Examination of ESTs suggests that this 3' region acts as a promoter giving rise to an

antisense transcript. Histone ChIP-seq data were downloaded from histone tracks (ENCODE for H1-ESC, and Zhu et al, Cell 2013 for histone marks in H9 derived neurons; GSE17312 and GSM733758, repectively). **D,** Graphs of mean fractional methylation of the DS-DM loci (sets defined by p<.005; delta AVG_Beta>.15) by age and disease status shown for fetal cerebrum, This graph shows no major age effects (the DS-DM is present at every age and the slopes for DS and control samples are similar) and indicates that strong fetal DS-DM identified through our case-control analyses is not due to accelerated aging in DS. Analogous results for adult brains, neurons and T cells are in Supplemental Figures S15 – S17 and Supplemental Table S11. **E,** Graphs of the mean fractional methylation of the 40 CpGs with increasing methylation over gestation and for which age and disease effects explained more than 80% of the variance (adjusted age p-value<0.05 and adj. R squared>=0.8). Data from the CpGs with decreasing methylation over gestation (14 CpGs) are shown in Suppl. Figure S12. In both sets of CpGs, the differential aging effect is explained by a smaller and negligible age effect in DS compared to the controls, with the slopes and Y-intercepts being most simply explained as early maturation of methylation patterns in the DS fetal brains. Linear coefficients for DS and control are reported separately. The coefficient reflects the difference in fractional methylation observed per 10 weeks for fetal cerebrum. The adjusted R squared reflects the good fit of the linear model and the p-value for the interaction term between age and disease reflects the differential age effect between DS and control fetal cerebrum.

Figure S14. Over-representation of brain-expressed and brain-repressed genes, compared to genes with pan-tissue expression patterns, in the set of genes with DS-DM in neurons.

Examples of each of the four types of expression patterns that we used to categorize the DM genes. Expression patterns for each gene were downloaded from the Allen Brain Atlas and BioGPS. A Z score (-μ/sd) was calculated for each gene to allow comparison between arrays; as such the overall genome mean expression is zero. The pie charts show the distribution of these patterns among the hypermethylated DS-DM genes in neurons and glia and their background distribution as estimated from the set of non-DM genes (450K BeadChip content) for which expression data could be obtained from both Allen Brain Atlas and BioGPS.

Figure S15. Analysis of CpGs with age-dependent methylation indicates premature maturation of methylation patterns, without accelerated epigenetic aging of these patterns, in DS.

A, Graphs of the mean fractional methylation of the 14 CpGs with decreasing methylation over gestation and for which age and disease effects explained more than 80% of the variance (adjusted age p-

value<0.05 and adj. R squared>=0.8). Data from the CpGs with decreasing methylation over gestation are in the left panel, and the results for CpGs with increasing methylation are in the right panel). In both probe sets, the differential aging effect is explained by a smaller and negligible age effect in DS but not in control, which suggests early maturation of the methylation status in the DS fetal brains. Linear coefficients for DS and control are reported separately. The coefficient reflects the difference in fractional methylation observed per 10 weeks for fetal cerebrum and per 10 years in T cell. Adjusted R squared (adj. R^2) reflects the good fit of the linear model and the p-value for the interaction term between age and disease, which reflects the differential age effect between DS versus control fetal cerebrum is reported. **B,** Graphs of the mean fractional methylation of CpGs with significant differential aging effect between DS and control in fetal cerebrum. The left panel shows positive differential age effect between DS and control and the right panel negative differential age effect. In fetal cerebrum, overall 56 CpGs showed significant differential age effect (adj. R-squared>0.8, adjusted age effect p-value<0.05, age effect>0.1 per 10 weeks, p-value of the interaction term <0.05 and difference of the age effect between DS and normal >0.1 per 10 weeks), including 26 CpGs, also identified as age dependent loci in normal samples. These data (age-dependent *hypo*methylation shown here and age-dependent *hyper*methylation shown in **Figure 4 of the main text**) confirm an early maturation and the absence of accelerating aging but interestingly also reveal loci with age effect in DS but not in control and loci with opposite age effect in DS versus control. **C,** In T cells, 168 CpGs showed significant differential age effect (adj. R-squared>0.8, adjusted age effect p-value<0.05, and p-value of the interaction term <0.05), including only 2 CpGs, also identified as age dependent loci in normal samples. A similar trend as in fetal cerebrum is observed, with the slopes and Y-intercepts showing no evidence of accelerated methylation aging in DS. Rather, the simplest interpretation of the data is that there is early maturation of methylation patterns in the DS T cells, occurring at some point before 20 years of age. The graphs show that after age 20 the methylation patterns take on a progressively younger appearance with advancing age in the adults with DS.

Figure S16. The strong DS-DM identified by case-control analysis in fetal cerebrum, adult FC and T-lymphocytes, is not due to accelerated aging.

A, Graphs of the mean fractional methylation of the CpGs with age effect in control adult FC and T cell.

Fractional methylation was averaged across the 39 CpGs with an age effect in control adult brains after univariate linear regression (R-squared>=0.8, p-value <=0.05). These CpGS showed very small fractional methylation changes over time (average=0.004 per 10 years) in control. No evidence of accelerated

methylation aging or differential aging effect in the adult DS brains is observed. In adult T cell, 1022 loci showed a significant age effect in control after univariate analysis with once again a negligible methylation changes per 10 years (average=0.02 per 10 years, **Suppl. Table 11c**). A modest differential age effect between DS and control is present (differential methylation=0.03 per 10 years, p=0.00007) with a slight decline in methylation levels with age in the controls and no decline in the DS cases. **B**, Graphs of mean fractional methylation of the DS-DM loci (sets defined by p<.005; delta AVG_Beta>.15) by age and disease status, shown for adult FC, and adult T cells. These graphs show no major age effects (the DS-DM is present at every age and the slope in DS and control are similar), and similar results were found for the adult cerebellar cortex samples (**Suppl. Table S11c**). Among the T cell DS-DM loci with hypomethylation in DS, a slight differential age effect between DS and control is observed (differential methylation=0.02 per 10 years, p=0.04) with a slight decline in methylation levels with age in the controls and no decline in the DS cases. Overall, these results indicate that strong DS-DM identified through our case-control analyses is not attributable to accelerated aging in DS. Statistical tests for more complex aging effects are described in the main text.

Figure S17. The strong DS-DM identified by case-control analysis in adult FC neurons is not due to accelerated aging.

Graphs of mean fractional methylation of the DS-DM loci (sets defined by p<.005; delta AVG_Beta>.15) by age and disease status, shown for adult FC neuron. These graphs show no major age effects: the DS-DM is present at every age and the slope in DS and control are similar.

Figure S18. A subset of the genes with DM show altered mRNA expression in the available adult or fetal tissues.

A, Expression of *STK19* mRNA in adult FC measured by Q-PCR, showing a significant decrease in the DS samples (left panel). CpG methylation of the *STK19* promoter region (cg04149916) in DS and control FC, shows hypermethylation in DS (right panel). The horizontal lines indicate the mean fractional methylation (AVG_Beta) values. **B,** Expression of *STK19* mRNA measured by Q-PCR in normal human astrocytes exposed to the indicated concentrations of 5aza-dC for 3 days, showing a dose-dependent increase in expression in response to the demethylating treatment. **C,** Left panel, expression of *NLGN2* in adult FC measured by Q-PCR, showing a significant increase in the DS samples. Right panel, CpG methylation of *NLGN2* promoter region (cg09568217) showing hypermethylation in DS neurons, which are the cell type that is known to express this gene. **D,** Left panel, expression of *GLI4* mRNA in fetal cerebrum measured by Q-PCR showing a significant increase in the DS samples. Right panel, methylation

values from the 450K data (cg24510518) showing hypermethylation of the 3' regulatory element in the DS samples. The "paradoxical" correlation of hypermethylation with increased *GLI4* expression may be explained by the fact that the 3' element acts as the promoter for an antisense RNA transcript (see Figure 6 in the main text). **E,** Map of the *EFNA3* gene and DS-DM and mRNA expression of *EFNA3* in DS and control adult cerebellar cortex. Hypomethylation in the promoter region (solid rectangle) and gains of methylation in the gene body (dashed rectangle) are associated with relative over-expression in DS compared to control cerebellum.

Figure S19. Gene ontology analysis and enrichment analysis for C-DM CpGs in the DS-DM gene sets.

A, Results from GO analysis of C-DM genes (cell type-specific DNA methylation) in normal neurons versus normal glia (with an absolute difference in methylation $\geq$ 0.5), showing significant enrichment of glia-specific genes in those *hypo*methylated in glia versus neurons, and significant enrichment of neuron-specific genes in the those *hypo*methylated in neurons. **B,** Pie charts showing the % of Down syndrome-specific DM (DS-DM) CpGs that overlap with cell type-specific (C-DM) CpGs in FC neurons and glia. There is very strong enrichment of C-DM CpGs in the sets of DS-DM CpGs identified in the DS vs. normal neuron and glia comparisons.

Figure S20. Gains of CpG methylation in DS FC glia and whole cerebellar cortex occur preferentially in sequences that reside in poised chromatin in human ES cells.

A, Pie charts showing enrichment of ChIP-seq peaks for specific histone marks in the sets of hypermethylated DS-DM loci in DS versus normal glia and neurons. Here we define poised chromatin as loci overlapping both H3K27me3 and H3K4me3 or H3K4me1 peaks and active chromatin as loci overlapping only H3K4me3 peaks and/or H3K4me1, without H3K27me3. The analysis was performed with ChIP-seq data from H1-hESC and N-HA (normal human astrocyte) cell lines, and H9-hESC-derived human neurons. In the set of loci hypermethylated in DS glia, there is significant enrichment of poised chromatin unique to H1-hESC, but not those unique to N-HA. Conversely, there is significant enrichment of active chromatin regions unique to N-HA, but not those unique to H1-hESC. These findings suggest that hypermethylation component of DS-DM (the majority of DS-DM CpGs) in glia occurs preferentially at genes that are silenced in stem cells and then actively transcribed upon cell differentiation. The results for the set of DS-DM loci that are hypermethylated in DS neurons are qualitatively and quantitatively different: we observed no enrichment for chromatin marks unique to H1-hESC or H9-hESC derived neurons, and in fact found a slight trend toward underrepresentation of poised chromatin unique to H1-hESC. **B,** Pie charts showing enrichment of ChIP-seq peaks for specific histone marks in the

sets of DS-DM loci in DS versus normal whole cerebellar cortex. The findings, with the *hyper*methylated DS-DM CpGs strongly enriched at loci that have a poised chromatin state in ES cells, parallel those for the hypermethylated DS-DM CpGs in the FC glia. The *hypo*methylated DS-DM CpGs show the opposite result, with a strong relative exclusion from such loci.

Figure S21. Two complementary and non-redundant approaches for TFBS enrichment analysis.

Left, the *de novo* motif search using HOMER tools is summarized. This approach allows identifying both known motifs and unknown potential DNA regulatory sequences. **Right**, enrichment analysis for known TF motif instances using ENCODE project ChIP-Seq data.

Figure S22. Mouse models: copy number analysis and lack of a dosage compensation-like effect in the duplicated chromosomal regions

Confirmation of the expected partial trisomies in Dp(10)1Yey and Dp(16)1Yey mouse brain DNA samples by copy number analysis of relative representation of reads in the whole genome bis-seq (WGBS) data. **A,** Coverage normalized by the total number of reads mapping to chromosome 10 in the indicated chromosomal region. The ratio of the normalized coverage in Dp(10) to the coverage in wt littermate mice is shown. In the *Prmt2-Pdxk* region a 1.5 fold increase of the coverage is observed only for Dp(10), confirming the expected segmental trisomy in this DNA sample. **B,** Coverage normalized by the total number of reads mapping chromosome 16 in the indicated chromosomal region. As expected, a 1.5 fold increase is observed in the region spanning *Lipi-Zfp295* in the Dp(16) DNA sample. While the coverage ratios show a 1.5 fold change reflecting the extra chromosomal domain, the methylation tracks show no obvious differences in fractional methylation across the duplicated genomic regions, indicating the absence of a dosage compensation-like effect.

Figure S23. Mouse models: Differential DNA methylation is not concentrated in the duplicated chromosomal regions

The modest hypermethylation in the Prmt2-Pdxk region compared to the rest of chromosome 10 is only slightly higher in both Dp(10) and Dp(16) than in control. Hypermethylation in the Lipi-Zbtb21 region compared to the rest of chromosome 16 is similar in Dp(16) and wt and slightly higher in Dp(10). These results confirm the absence of a dosage compensation-like effect.

Figure S24. Mouse models: Mild global changes in methylation are evenly distributed across the chromosomes, and strong DM is focal but not concentrated in promoter regions

A, Average fractional methylation by chromosome in control, Dp(10) and Dp(16) mice (WGBS). There is very mild relative *hyper*methylation in Dp(10), and slight relative *hypo*methylation for Dp(16) across all of the autosomes. Overall, there are mild global methylation differences between control and DS mouse models. The Dp(10) mouse cerebrum shows a mild global hypermethylation compared to the control mouse cerebrum (Δmean fractional methylation=+0.0017), while Dp(16) shows a mild global hypomethylation (Δmean fractional methylation=-0.01). This result is also seen at the level of the individual chromosomes. These data indicate that global methylation changes are present, but are quite mild compared to the locus-specific impact of the duplication of these Hsa21 syntenic regions. The increase in methylation in Dp10, but not in Dp16, may be due to the group of methylation pathway genes in the Dp10 duplication, including Dnmt3l and several important folate/methyl-donor pathway genes, which we discuss in the main text. **B,** At High-stringency criteria (>3 DM CpGs with $p<0.05$), hypermethylated CpGs in Dp(10) and Dp(16) compared to control mouse were slightly enriched in CGI, CGI shores and gene bodies but relatively under-represented in promoter regions. Hypomethylated CpGs in Dp(10) and Dp(16) were modestly enriched in CGI shores and gene bodies but under-represented in CGI and promoter regions. Percentage of DM CpGs overlapping CGIs, CGI shores (<2kb from CGI), CGI shelves (<4kb), promoter regions (+/-1kb from gene transcription starting sites) and gene bodies in Dp10 and Dp16. The random expectation was calculated from all CpGs with at least 20X coverage. $*10^{-100}<p<10^{-20}$, $**<10^{-100}$

Figure S25. Partial but significant overlap between hypermethylated DS-DM in humans and DM in the chromosome-engineered mouse models of DS.

A, In the mouse WGBS data we ranked each 1 kb genomic window for the strength of hypermethylated DM. Using the sets of ranked loci, we asked whether the human hypermethylated DS-DM genes identified in our comparison between DS vs. control human brain cells and tissues (adult FC, neurons, glia and fetal cerebrum) were statistically enriched among the mouse DM genes. There is progressive enrichment for relative overlap with human DS-DM genes as the stringency requirements for DM in the mouse data are increased. The p-values for combined Dp(10) and Dp(16) are shown, but each line by itself also showed highly significant enrichment p-values. **B,** The Venn diagram on the left shows that using a Fisher Exact test cutoff of $p<.05$ for DM of individual CpGs in the mouse WGBS data, 28% of the human hypermethylated DS-DM genes identified in neurons or glia are differentially methylated in the

same direction in whole newborn cerebrum from one or both of the segmental trisomy mice. The substantial but partial overlap between Dp(16) and Dp(10) DM genes reflects different contributions to DM from duplications of different chromosomal regions.

Figure S26. Mouse models: co-mapping of DM in the GPT-*LRRC24/Gpt-Lrrc24 and MZF1/Mzf1 loci*

A, Maps of the *LRRC24/Lrrc24* genes and their flanking genes in human and mouse. A cluster of CpGs in the gene body *LRRC24/Lrrc24* genes is concordantly hypermethylated in human brain cells from both Dp(16) and Dp(10) (rectangles). A other cluster in the gene body *GPT/Gpt* genes is concordantly hypermethylated in human brain cells from Dp(10) but not Dp(16). Conversely, additional hypermethylaled and hypomethylated CpGs are observed in Dp(16) and Dp(10) mice but not in human brain cells. Only CpGs in 1 kb windows containing orthologous CpGs queried by the Illumina 450K BeadChips are considered. **B,** Maps of the *MZF1/Mzf1* genes and their flanking genes in human and mouse. The graphs were made as in main **Figure 5**.

Figure S27. Mouse models: co-mapping of DM in the *FAM83H/Fam83h* and *DNMT3L-AIRE/Dnmt3l-Aire* loci

A, Maps of the *FAM83H/Fam83h* genes and their flanking genes in human and mouse. A cluster of CpGs in the gene body *LRRC24/Lrrc24* genes and another cluster spanning *CCDC166*/*Ccdc166* genes are concordantly hypermethylated in human brain cells from both Dp(16) and Dp(10) (rectangles). Conversely, additional hypermethylaled and hypomethylated CpGs are observed in Dp(16) and Dp(10) mice but not in human btain cells. Only CpGs in 1 kb windows containing orthologous CpGs queried by Illumina 450K BeadChips are considered. **B,** Maps of the *CPT1B/Cpt1b* genes and their flanking genes in human and mouse. A cluster of CpGs between *CPT1B* and *CHKB* is concordantly hypermethylated in human brain cells from Dp(16)1Yey. In Dp(10)1Yey, both hypermethylation and hypomethylation are observed. Additionally, both mice showed hypermethylation in the body of Chkb gene. The mouse maps span 514 CpGs with WGBS coverage>20X in this chromosome region; of these 22 showed DM.

Figure S28. Mouse models: co-mapping of DM in the *PCDHGA2*/*Pcdhga2* loci

Maps of the *PCDHGA2*/ *Pcdhga2* genes in human and mouse. A cluster of CpGs in the *PCDHGA2* promoter is concordantly hypermethylated in human brain cells and both Dp(10)1Yey and Dp(16)1Yey (solid rectangles). At other locations some differences in methylation patterns are seen between Dp(10)1Yey and Dp(16)1Yey (dashed rectangle). In the mouse models, hypermethylated regions are also present in *Pcdhga1* and *Pcdhga3*, but these regions only show subthreshold hypermethylation in

human brain cells. The mouse maps span 251 CpGs with WGBS depth >20X in this chromosome region; of these 39 showed DM by the above criteria.

Figure S29. Methylation values (WGBS) in the *Stk19*, *Cpt1b*, and *Lrrc24* DM regions in wt, Dp(10) and Dp(16) mouse brains

Hypermethylation in *Stk19* region is coincident with an intermediate methylated CG island shore in wt mouse. DM in *Cpt1b* promoter, in *Chkb* overlap an intermediate methylated region and CpG shore, respectively. Hypermethylation in *Lrrc24* is coincident with an intermediate methylated region.

Figure S30. Methylation values (WGBS) in the *Mzf1*, *Fam83h*, and *Celsr3* DM regions in wt, Dp(10) and Dp(16) mouse brains

The multiple hypermethylated regions in Mzf1 and Fam83h overlap with intermediate or high methylated CpG shores and regions. DM clusters in Celsr3 are coincident with low methylated CGIs.

Figure S31. Methylation values (WGBS) in the *Pcdhga*2 DM region in wt, Dp(10) and Dp(16) mouse brains

Hypermethylation in the *Pcdhga*2 promoter is coincident with a low methylated CGI in wt mouse.
