# Supplementary material for: Trans effects of chromosome aneuploidies on DNA methylation patterns in human Down syndrome and mouse models
Source: Genome Biol. 2015 Nov 25;16:263. doi: 10.1186/s13059-015-0827-6 (PMC4659173; doi:10.1186/s13059-015-0827-6)
Supplement: Additional file 2: — Supplemental tables. (ZIP 4474 kb) [file 13059_2015_827_MOESM2_ESM.zip › Table .pdf]

Supplemental Tables

Table S1. Brain samples analyzed for DNA methylation.

Samples were triaged for the several assays based on the amount of tissue available. BT ID numbers are for the Tycko laboratory tissue bank; collaborator numbers are for the brain bank at the NYSIBR and the fetal tissue bank at UCL. Some adult brain samples have two or three ID numbers, representing two separate nuclear FACS preparations from the same brain slice. Five additional control adult brain samples in this same age range were used to supplement the Q-PCR analyses. *, Only Bis-seq data.

Table S2. Mean fractional methylation in DS and control samples of each tissue and cell type.

All probes were considered in calculating array-wide methylation, with the exception of probes overlapping known SNPs or mapping to the X or Y chromosome. AVG_Beta values have been adjusted for batch effects (see Methods). The T-test p-values for DS vs. controls were generated using the mean methylation value for each sample. There is a small but significant increase in array wide methylation in DS compared to normal brain cells, but not in DS compared to normal T cells, where the direction of the effect is toward hypo- rather than hyper-methylation.

Table S3. Loci with DS-associated differential methylation (DS-DM) in whole adult frontal cortex grey matter analyzed on the 450K methylation arrays.

This is a complete list of DS-DM loci. Lenient criteria for differential methylation between DS cases and controls (DS-DM), applied to the batch-corrected AVG_Beta values (see Methods), with T-test p values<.05; delta AVG_Beta>0.15. See the main text for analyses using more stringent p-value cut-offs. Multivariate regression was used to calculate disease status p-value adjusted for sex and sex p-value adjusted for disease status. Most of the DS-DM CpGs (93%) remain significant after adjustment for sex.

Table S4. DS-DM loci in whole adult cerebellar folial cortex analyzed on the 450K methylation arrays.

Criteria for DS-DM, applied to the AVG_Beta values, were T-test p values<.05; delta AVG_Beta>0.15. These samples were run in a single batch. See the main text for analyses using more stringent p-value cut-offs. Multivariate regression was used to calculate disease status p-value adjusted for sex and sex p-value adjusted for disease status. Most of the DS-DM CpGs (98%) remain significant after adjustment for sex.

Table S5. DS-DM loci in the glial cell fraction (NeuN-negative) analyzed on the 450K methylation arrays.

Criteria for DS-DM, applied to batch-corrected AVG_Beta values, were: T-test p-values<.05; delta AVG_Beta>.15. See the main text for analyses using more stringent p-value cut-offs. Multivariate regression was used to calculate disease status p-value adjusted for sex and sex p-value adjusted for disease status. Most of the DS-DM CpGs (94%) remain significant after adjustment for sex.

Table S6. DS-DM loci in neurons (NeuN-positive) analyzed on the 450K methylation arrays.

Criteria for DS-DM, applied to batch-corrected AVG_Beta values, were: T-test p-values<.05; delta AVG_Beta>.15. See the main text for analyses using more stringent p-value cut-offs. Multivariate regression was used to calculate disease status p-value adjusted for sex and sex p-value adjusted for disease status. Most of the DS-DM CpGs (98%) remain significant after adjustment for sex.

Table S7. DS-DM loci in T-lymphocytes analyzed on the 450K methylation arrays.

Criteria for DS-DM, applied to the AVG_Beta values, were: T-test p-values<.05; delta AVG_Beta>.15. Multivariate regression was used to calculate disease status p-value adjusted for sex and sex p-value adjusted for disease status. Most of the DS-DM CpGs (99.5%) remain significant after adjustment for sex.

Table S8. Results of BS/OXBS analysis of DS versus control cerebellar cortex on 450K methylation arrays.

A, Analysis of the results in this experiment using the original DS-DM probes (CpGs) from the larger case-control series. We further required that the probes pass QC pval<.005 in the BS-OXBS BeadChip data, and that in this smaller set of 3 vs. 3 cerebellar cortex samples the CpGs pass p<.05 uncorrected, for the BS AVG_Beta values (5mC+5hmC), in DS vs. control. The main findings are that both 5mC and 5hmC contribute to the net DS-DM, and the direction of the DS-DM is the same for 5mC and 5hmC for a large majority of the differentially methylated CpGs. **B,** BS/OXBS analysis of DS versus control cerebellum on 450K methylation arrays, with T-test and absolute difference cutoffs applied directly to the BS/OXBS data from this 3 vs. 3 series, to identify a set of DS-DM CpGs. This table shows the mean levels of 5hmC at each significant CpG in DS and control samples, as indicated by the mean OXBS AVG_Beta values. There is substantial overlap of this probe set with the set of DS-DM loci from the larger case-control series analyzed by the conventional 450K procedure. **C,** BS/OXBS analysis of DS versus control cerebellum on 450K methylation arrays, with T-test and absolute difference cutoffs applied directly to the BS/OXBS data from this 3 vs. 3 series to identify a set of DS-DM CpGs. This table shows the mean levels of 5mC at each significant CpG in DS and control samples, as indicated by the mean AVG_Beta values for BS minus the mean AVG_Beta values for OXBS. There is substantial overlap of this probe set with the set of DS-DM loci from the larger series.

Table S9. DS-DM loci in DS versus control mid-gestation fetal cerebrum analyzed on the 450K methylation arrays.

Criteria for DS-DM, applied to the batch-corrected AVG_Beta values, were: T-test p-values<.05; delta AVG_Beta>.15. See the main text for analyses using more stringent p-value cut-offs. Multivariate regression was used to calculate disease status p-value adjusted for sex and sex p-value adjusted for disease status. Most of the DS-DM CpGs (90%) remain significant after adjustment for sex.

Table S10. DS-DM genes analyzed for overlap with genes showing developmentally regulated mRNA expression in the human brain.

The DS-DM gene sets were generated using cut-offs of p<0.001; delta AVG_Beta>.15; probes with missing values excluded. Gene expression data were from the GDS3113 dataset downloaded from NCBI_GEO.

Table S11. Analysis of developmental stage-dependent and age-dependent CpG methylation.

A, CpGs with methylation changes across developmental stages in control fetal brains were first identified using univariate linear regression (p-value<0.05, methylation changes>0.1 per 10 weeks, adjusted R-squared>0.8). Multivariate regression was also performed on these 5657 significant loci. The coefficient for age, and the interaction term are reported per week. Among them, 4230 showed significant methylation changes within developmental stage after adjustment for disease status (p-value<0.05, methylation changes>0.1 per 10 weeks in fetal), including 54 with an adjusted R-squared>0.8 and 26 loci with significant differential aging effect in DS (significant interaction term).

Multivariate analysis also identified 30 additional CpGs with significant differential aging effect (adj. R-squared>0.8, adjusted age effect p-value<0.05, p-value of the interaction term <0.05 and difference of the age effect between DS and normal >0.1 per 10 weeks) but that were not detected after univariate analysis in control samples. Overall, these data suggest an early maturation and the absence of accelerating aging (also refer to **Suppl. Figs. S11 and S12**) but interestingly also reveal loci with age effects in DS but not in controls and loci with opposite age effects in DS versus controls. In addition, none of the age dependent CpG overlaps with the strong fetal DS-DM CpGs (p<0.001 and absolute difference >0.15) and only 2 CpGs with differential aging effects overlap with the strong fetal DS-DM CpGs. **B**, CpGs with age-dependent methylation in control adult FC were identified using univariate. The coefficient for age (methylation change) is reported per year. Although 39 CpGs passed our p-value and R-squared criteria, the methylation changes per 10 years were negligible (maximum=0.02), with no evidence of accelerated aging in the DS brains. Multivariate analysis only identified 3 CpGs with a significant differential age effect between DS and control FC (adj. R-squared>0.8, adjusted age effect p-value<0.05, p-value of the interaction term <0.05). None of these CpGs overlaps with the strong FC DS-DM loci. **C**, Using the same approach and criteria as used in adult FC, 348 CpGs with modest age-dependent methylation (maximum methylation changes per 10 years =0.05) in control adult cerebellum were identified using univariate analysis. The coefficient for age (methylation change) is reported per year. Multivariate analysis only identified 46 CpGs with significant differential aging effects between DS and control cerebellum (adj. R-squared>0.8, adjusted age effect p-value<0.05, p-value of the interaction term <0.05) which correspond mostly to loci with aging effects in controls but not in DS. Thus, no evidence of accelerated aging in the DS cerebellum is observed. In addition, no overlap between DS-DM and age-dependent methylation is observed and only 6 CpGs with differential aging effects overlap with cerebellar DS-DM CpGs. C, In T cells, 1022 CpGs with age-dependent methylation was identified by

univariate regression in control T cell and 242 CpGs with a significant differential aging effect between DS and control T cells, which again correspond mostly to loci with age effects in normal but not in DS. No evidence of accelerated aging in the DS T cells is observed (see also **Suppl. Figs. S11 and S12).** In addition, no overlap between DS-DM and age-dependent methylation is observed and only 41 CpGs with differential aging effects overlap with the much larger number of DS-DM CpGs in this cell type.

Table S12. Top enriched gene sets from GSEA analysis of genes with DS-DM.

All gene sets passed FDR<0.0001**.**

[1]UP upregulated genes; [2]DN. downregulated genes; bold font indicates involvement of the PRC2 polycomb repressive complex.

Table S13. Enrichment for specific TFBS in the DS-DM gene sets.

A, De novo predicted motifs (HOMER) over-represented in the sets of fetal cerebrum, adult FC brain cells, cerebellum and T cell DS-DM loci. Since the large majority of the DS-DM is hypermethylation, we did not perform a separate analysis for the few hypomethylated loci in fetal cerebrum, neuron and glia. Only significant ($p<10^{-12}$) enriched de novo predicted motifs matching known TF motifs with an alignment score>0.65 and present in a 200bp window of at least 20% of the DS-DM CpGs are shown. Among each enriched *de novo* motif instance, the strongest enriched TF peak among each enriched *de novo* motif instances is indicated (ENCODE ChIPSeq data). Statistical cutoffs defining each set of DS-DM loci were: Difference >0.15 and p-value<0.001. **B**, Known TFBS (ENCODE) enrichment in the sets of fetal cerebrum, adult FC brain cells, cerebellum, T cell DS-DM loci and in the set of DS-DM loci shared across multiple tissues. TF motif instances coordinates were downloaded from the ENCODE project accessible via the UCSC Genome Browser.

Table S14. Genes with DS-DM overlapping across multiple tissues and cell types.

Table S15. Overlap of human DS-DM from 450K Beadchip data with DM in the segmental trisomy mouse models from WGBS data.

Table S16. Bisulfite PCR primers.

Table S17. Q-PCR primers.

Reference

Chen, C., K. Grennan, et al. (2011). "Removing batch effects in analysis of expression microarray data: an evaluation of six batch adjustment methods." PloS one **6**(2): e17238.
