# Supplementary material for: Trans effects of chromosome aneuploidies on DNA methylation patterns in human Down syndrome and mouse models
Source: Genome Biol. 2015 Nov 25;16:263. doi: 10.1186/s13059-015-0827-6 (PMC4659173; doi:10.1186/s13059-015-0827-6)
Supplement: Additional file 3: — Supplemental bioinformatics. (PDF 155 kb) [file 13059_2015_827_MOESM3_ESM.pdf]

## Supplemental Bioinformatic Results

NOTE: The sections below contain some repetition from the Results in the main text, but also contain additional details concerning the several types of bioinformatic enrichment analyses.

### Analysis of cell-type-specific DM among the DS-DM genes

To ask whether the sets of loci with cell-type-specific DM (C-DM), identified in our comparisons of *normal* neurons versus glia, might be enriched for genes involved in neuronal and glial function we performed gene ontology annotation using DAVID on the set of 1773 genes showing a fractional methylation difference > 0.5 and p<0.001 (FDR<0.002, ~top 10% gene with highest methylation change) in neurons versus glia and found that the GO terms most strongly enriched in the set of genes *hypo*methylated in neurons included post-synaptic density, neurotransmitter secretion and calcium ion transport, while the groups enriched in the set of genes *hypo*methylated in glia included tight junction adhesion and endocytosis (**Suppl. Fig. S19**). We found a 4-fold and 2.5-fold enrichment of C-DM CpGs in the sets of DS-DM CpGs identified in the DS versus normal neuron and glia comparisons, respectively (70% vs 16%, p-value= $3 \times 10^{-159}$ and 34% to 16%, p-value= $1.5 \times 10^{-23}$ ). In our DS-DM gene set from cerebellum, both hyper- and hypo-methylated loci were likewise enriched in C-DM CpGs (3-fold; 54% vs 16%, p< $1 \times 10^{-999}$ and 41% vs 16%, p= $1.5 \times 10^{-49}$, respectively). We found similar fold enrichments when we restricted our set of C-DM loci to absolute methylation differences > 0.35 and 0.5. Thus, the sets of loci with DS-DM in adult DS vs. control FC neurons and glia, and cerebellar cortex, are all highly enriched in genes whose methylation is dynamically regulated during cell type specification in normal brain development.

**Analysis of stage-specific DM among the DS-DM genes**

We next generated a gene set of loci with strong stage-specific DM (S-DM; hyper- or hypomethylation) in the control adult compared to control fetal cerebral cortex samples (top 1000 probes with p-values<=0.001 (FDR<0.002), ranked by absolute methylation change; corresponding to 457 genes with an absolute change>=0.56) and repeated our enrichment analysis of the genes with DS-DM (hypermethylation) in DS brain cells against this gene list, using the 450K total probe content as the denominator. This procedure revealed a 4.6-fold enrichment for genes with developmentally regulated CpG methylation levels in the DS neuron hypermethylated gene set (10.16% in hypermethylated genes versus 2.2% in non DM genes, p= $2 \times 10^{-5}$) and enrichment in the DS adult glia and fetal FC hypermethylated gene sets (3.6 fold, p= $6.482 \times 10^{-7}$ and 3.3 fold, p= 0.032, respectively). In the cerebellum, both hyper- and hypo-methylated gene sets were enriched for developmentally regulated CpG methylation (3.2 fold, p= $4.845 \times 10^{-13}$ and 3.6 fold, p= $8.209 \times 10^{-7}$, respectively), and these strong enrichments were reproduced at the probe level (individual CpGs). Results were similarly positive in the all DS-DM (*hypo*- and *hyper*methylated loci) gene sets when assessing enrichment using the top 4000 probes (1606 genes) that showed S-DM in the normal brains. Thus, methylation differences in DS compared to control brain cells preferentially affect genes whose CpG methylation increases between the fetal and adult stages of normal brain development.

**Analysis of chromatin features among the DS-DM genes**

To further test this consistent theme of altered methylation in DS brain cells occurring in a non-random manner at developmentally dynamic loci, we used data from ENCODE and related

projects, accessed via the UCSC Genome Browser, as well as from Zhu and colleagues [1], to assess the enrichment of specific histone modifications in the set of DS-DM loci from our comparison of DS versus control glia and neurons ($p<0.001$, probes with missing values removed). Probes from the 450K arrays that overlapped with H3K4me1, H3K4me3, H3K27me3, and H3k27ac ChIP-seq peaks in human embryonic stem cells (H1-hESC) and not in normal human astrocytes (NH-A), as well as those detected in H1-hESC and not NH-A, were identified. In this analysis we deliberately excluded peaks that were shared between cells types, as they were seen as unlikely to be related to glial cell differentiation. Similar probe sets were generated separately with ChIP-seq data from H9-hESC derived neurons [1]. We further categorized peaks as marking active promoters (H3K4me3 alone), poised or "bivalent" promoters (H3K4me3/H3K27me3), active enhancers (H3K4me1/H3K27ac), intermediate enhancers (H3K4me1 alone), and poised enhancers (H3K4me1/H3K27me3) as described by Bernstein et al. and by Zentner et al. [2, 3]. We found that loci hypermethylated in DS glia were significantly enriched in active promoter regions unique to NH-A, but not those unique to H1-hESC, and were enriched in poised promoter regions that were uniquely repressed by the presence of the polycomb repressive complex (PRC2) recognition mark H3K27me3 in H1-hESC, but not for those bearing this chromatin mark in differentiated NH-A (**Suppl. Fig. S20**). Similarly, we found that the hypermethylated loci were enriched in poised enhancer regions unique to H1-hESC but not those unique to NH-A (promoters and enhancers combined in **Suppl. Fig. S20**). We performed similar analysis using histone ChIP-Seq data available in HUVEC and found a similar enrichment of regions poised in ES cell but activated in endothelial cells, among glia (non-neuronal) hypermethylated CpGs. Taken together, these results show that a group of

genes that are normally repressed by the PRC2 complex in ES cells and then activated during normal glial differentiation are preferentially subject to DS-DM (specifically, hypermethylation) in DS glia. These enrichments were not observed for the neuron DS-DM CpGs (**Suppl. Fig. S20**).

**Gene set enrichment analysis of DS-DM genes**

GSEA can be useful for addressing mechanisms of epigenomic changes. Here and in the analysis of TFBS that follows we found it informative to analyze in parallel the data from brain tissues and cells and from the heterologous cell type, CD3-positive T cells. Using lenient criteria (uncorrected p<.005 in all 3 datasets) we found ~15% overlap of DS-DM genes between the combined glia and neuron gene lists and the T cell DS-DM genes (**Suppl. Table S12**). The results of GSEA applied to our 5 types of brain DS-DM gene sets and to the T-cell DS-DM data are summarized in **Supplemental Table S13**. From the identities of the top-ranked gene sets in each comparison the overwhelmingly positive finding is enrichment of PRC2 target genes in the GSEA for all DS-DM datasets that include glia. In the GSEA for the neuron and T cell DS-DM gene set there is weaker enrichment for this chromatin mark.

**Comparison of the findings for methylation age effects in the current study versus the study by Horvath et al.**

A recent study by Horvath et al. utilized 450K BeadChips to compare CpG methylation patterns in unfractionated cerebral cortex samples from 4 DS versus 17 control brains and concluded that DS is associated with accelerated epigenetic aging [4]. Our conclusions differ from that study. In that study, “DNAm age” was estimated across 353 “clock” CpGs. This estimate corresponds to a weighted average of fractional methylation of age dependent CpGs identified

across multiple tissues in a training cohort (Genome Biology 2013, 14:R115). While we have no issues with the Genome Biology paper (which did not deal with DS), based on our reanalysis of the data from the more recent paper [4], in fact no accelerated aging of methylation patterns is observed in their DS brain samples. The details are as follows:

In this paper 56 control and 15 DS samples from 6 different brain areas were included. These samples were derived from 17 controls but only 4 DS patients (each sampled in several regions). Since treating multiple samples from the same patient as independent data leads to an artificial increase of the sample size and decrease of inter-individual variability between time points, we focused here only on their data from a single brain area (frontal cortex). While in Horvarth *et al* paper, age acceleration effect for each subject was estimated by the vertical distance to the regression line, here we estimated age acceleration effect by the slope of the linrear regression. Indeed, a higher distance to the control regression line may reflect only a higher methylation level at baseline (intercept) with similar slopes in the two groups, this situation suggesting early maturation rather than accelerated aging. This difference is not trivial – the mechanisms underlying early maturation of methylation patterns (as we have observed in fetal brains and inferred in adult T cells) are likely to be different from those involved in bona fide biological aging. In fact, the actual graph shows two parallel lines, similar to those of our DS-DM CpGs (**our Suppl. Figs. S15-S17**), with no evidence of accelerated aging.

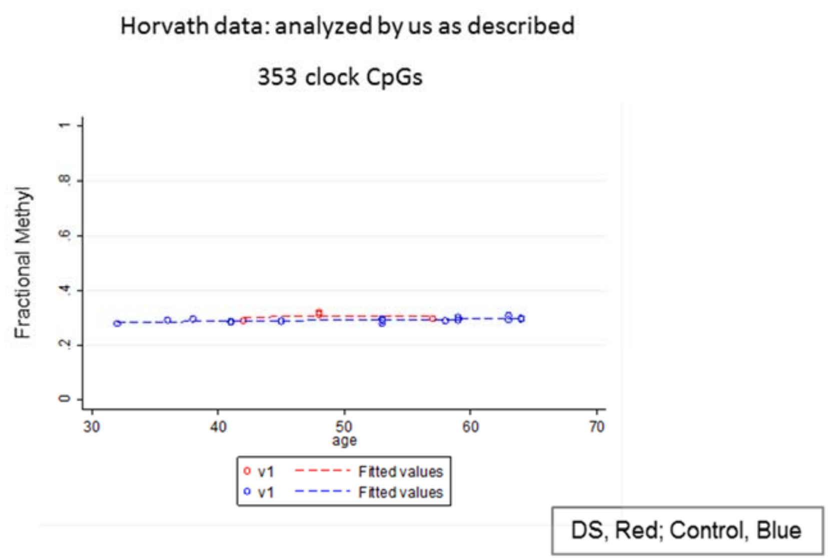


Our data

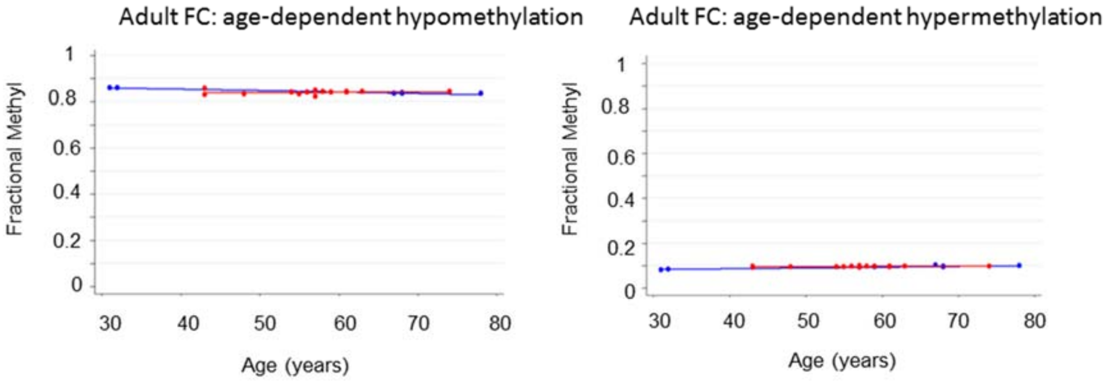


In addition, to estimate the effect size of aging on methylation changes, we used the same approach as for our dataset to search for correlations between age and DNA methylation. We averaged the methylation across the “clock” set without applying any weigh coefficient and carried out linear regression for DS and controls. Using this approach, the conclusions are more similar to ours, with negligible methylation change in controls (0.004 per 10 years, p=0.04). Although the DS sample size in their series (N=4) is too small to draw firm conclusions, we observed the same trend as in our data with a smaller age effect (0.002 per 10 years, p=0.8). Based on these analyses of the Horvath data, plus our own data from fetal and adult brains and

adult T cells described in the main text and shown in the Supplemental Figures, we conclude that there is early maturation of methylation patterns, but no accelerated aging of these patterns, in DS brains and T lymphocytes.

## REFERENCES


1. Zhu J, Adli M, Zou JY, Verstappen G, Coyne M, Zhang X, Durham T, Miri M, Deshpande V, De Jager PL, et al: **Genome-wide chromatin state transitions associated with developmental and environmental cues.** *Cell* 2013, **152:**642-654.
2. Zentner GE, Tesar PJ, Scacheri PC: **Epigenetic signatures distinguish multiple classes of enhancers with distinct cellular functions.** *Genome research* 2011, **21:**1273-1283.
3. Bernstein BE, Mikkelsen TS, Xie X, Kamal M, Huebert DJ, Cuff J, Fry B, Meissner A, Wernig M, Plath K, et al: **A bivalent chromatin structure marks key developmental genes in embryonic stem cells.** *Cell* 2006, **125:**315-326.
4. Horvath S, Garagnani P, Bacalini MG, Pirazzini C, Salvioli S, Gentilini D, Di Blasio AM, Giuliani C, Tung S, Vinters HV, Franceschi C: **Accelerated epigenetic aging in Down syndrome.** *Aging Cell* 2015, **14:**491-495.
